# An activity labelled molecular networking strategy-assisted discovery of antiplatelet aggregation-active components from *Allium chinense* G. Don

**DOI:** 10.3389/fnut.2026.1815132

**Published:** 2026-05-07

**Authors:** Yan Xu, Beibei Zhang, Liangliang He, Qiwen Zhang, Yongjie Yang, Zhihong Yao, Jing Yang, Qi Wang, Zifei Qin

**Affiliations:** 1 Department of Hematology, the First Affiliated Hospital of Zhengzhou University, Zhengzhou, China; 2 Department of Pharmacy, Henan Province Engineering Research Center of Application & Translation of Precision Clinical Pharmacy, The First Affiliated Hospital of Zhengzhou University, Zhengzhou, China; 3 State Key Laboratory of Bioactive Molecules and Druggability Assessment, International Cooperative Laboratory of Traditional Chinese Medicine Modernization and Innovative Drug Development of Ministry of Education (MOE) of China, Guangdong Basic Research Center of Excellence for Natural Bioactive Molecules and Discovery of Innovative Drugs, Guangdong Province Key Laboratory of Pharmacodynamic Constituents of TCM and New Drugs Research, College of Pharmacy, Jinan University, Guangzhou, China

**Keywords:** active steroidal saponins, *Allium chinense* G. Don, network pharmacology, PI3K/Akt pathway, targeted molecular networking

## Abstract

**Introduction:**

*Allium chinense* G. Don (ACGD) is a famous Chinese medicine that shares homology with medicine and food. However, the mechanism of ACGD-related components in antiplatelet aggregation remains unclear.

**Methods:**

This study identified steroidal saponins using a targeted molecular networking (TMN) approach and explored the mechanism of antiplatelet aggregation.

**Results:**

ACGD samples (1 mg/mL) extracted using different solvents (water, 30% methanol, 50% methanol, 70% methanol, and 100% methanol) all exhibited inhibitory effects on platelet aggregation induced by adenosine diphosphate, arachidonic acid, and collagen. The ACGD fraction extracted with 70% methanol (1 mg/mL) displayed the strongest inhibitory effects. Furthermore, a total of 36 steroidal saponins were visualized based on TMN analysis. These components were identified or tentatively characterized according to their retention behaviors, diagnostic ions, and fragmentation patterns. Among them, laxogenin-related furostanol and spirostanol saponins were the most abundant saponins in ACGD. A total of 15 saponins with antiplatelet aggregation activity, high content levels, different structural types, or authentic standards were selected to perform the network pharmacology analysis. The PI3K/Akt pathway is one of the most important signaling pathways in platelet activation. Moreover, based on CCK-8 assays in MEG-01 cells, compounds T3-12, T3-14, T3-15, T3-16, and T3-17 were non-toxic and did not induce proliferation at 5–20 μM. Moreover, compounds T3-15, T3-16, and T3-17 at 20 μM caused downregulation of the *p*-PI3K protein, whereas components T3-14, T3-16, and T3-17 at 20 μM decreased *p*-Akt in MEG-01 cells.

**Discussion:**

The TMN approach, integrated with diagnostic ions, was successfully applied to characterize target steroidal saponins in ACGD. Compounds T3-16 and T3-17 were confirmed to be active components in ACGD in antiplatelet aggregation through the PI3K/Akt pathway.

## Graphical abstract

**Figure:**
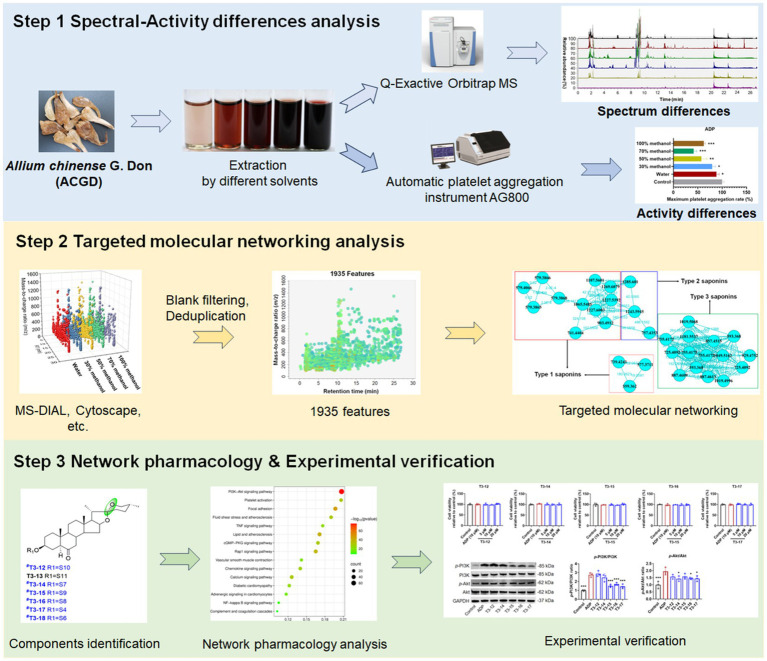

## Introduction

*Allium chinense* G. Don (ACGD) is one source of the herbal medicine “Xiebai” in China. Native to East Asian countries, it is now cultivated in North America and Southeast Asia (1–3). Historically, ACGD has been used in the management of cardiovascular diseases owing to its antiplatelet aggregation, lipid-lowering, and anti-atherosclerotic properties, along with its ability to protect against myocardial injury and block *α* and *β* receptors (4–6). In addition to cardiovascular benefits, ACGD exhibits antidepressant, antitumor, antibacterial, insecticidal, and antioxidant activities (1–3). In clinical practice, it is frequently formulated with Trichosanthes kirilowii to create a classic prescription for treating coronary heart diseases (7–10). Furthermore, ACGD is a popular vegetable with significant nutritional value, commonly consumed in stir-fries or processed into canned goods (1–3). Owing to its nutritional and medicinal values, it is praised as the *Ganoderma lucidum* of vegetables (1–3). In 2025, China’s national production reached approximately 400,000 tons. Because of its rich therapeutic and nutritional values, more studies are focusing on its active chemical compounds and pharmacological activities.

Numerous studies have demonstrated that ACGD is exceptionally rich in small molecules (such as steroidal saponins, sulfur-containing volatile oils, and nitrogenous compounds) and macromolecules (including polysaccharides) (11–14). These compounds exhibit a wide range of biological activities, consistent with the traditional clinical applications of ACGD. For example, macrostemonoside A inhibited adenosine diphosphate (ADP)-induced rabbit platelet aggregation with an IC_50_ value of 0.065 mmol, whereas chinenoside II and III prolonged ADP-induced rabbit blood coagulation *in vitro* (1–3). Furthermore, certain spirostanol saponins displayed significant inhibitory effects against platelet aggregation induced by arachidonic acid (AA), ADP, and collagen in human blood (4). Additionally, the structure–activity relationships of the platelet aggregation–inhibitory activities of spirostanol and furostanol saponins in ACGD have been summarized previously (4). Inspired by the considerable antiplatelet activity of ACGD, this study aimed to identify its active components and elucidate their mechanisms of action, particularly because limited research has been conducted in this area.

Recently, the molecular networking approach has emerged as an efficient compound annotation method for complex liquid chromatography-mass spectrometry (LC–MS) data (15–17). Global Natural Products Social Molecular Networking (GNPS), a prominent platform for molecular networking analysis, has been widely used to process LC–MS data (15–17). Compared to conventional molecular networking methods, our established strategy integrates hand-in-hand alignment with targeted molecular networking (TMN) (18). This approach offers significant advantages for the rapid screening of characteristic compounds within complex samples (18). First, all raw LC–MS data were processed simultaneously through peak detection, deconvolution, background subtraction, and alignment to generate an aligned result file free from matrix noise interference. This pre-processing step is termed “hand-in-hand alignment” (18). Second, based on the MS^2^-similarity adjacency matrix, a TMN method was applied to characterize components of interest (18). In the present study, this strategy was successfully used to profile the characteristic components of ACGD.

In this study, to explore the mechanisms underlying the antiplatelet effects of ACGD, we conducted a systematic series of experiments. First, the ACGD samples were extracted using various solvents, specifically deionized water and 30, 50, 70, and 100% methanol. These extracts were evaluated for their ability to inhibit platelet aggregation and analyzed to determine their LC–MS profiles. Based on the resulting spectrum–activity relationships, the most potent fraction was selected for subsequent targeted molecular networking analysis. Following rigorous data processing steps (such as blank subtraction and deduplication), the target precursor ions were identified or tentatively characterized based on their retention behaviors and fragmentation patterns. Guided by the bioactivity results, representative active components were selected for network pharmacology analysis. A key predicted signaling pathway was subsequently chosen for *in vitro* experimental validation using MEG-01 cells. Taken together, this study successfully elucidated the active components of ACGD and validated the mechanisms driving their antiplatelet aggregation effects.

## Materials and methods

### Materials and chemicals

Fresh *Allium chinense* G. Don bulbs were collected from Xinjiang (Jiangxi, China) and identified by Prof. Zhihong Yao and processed into dried samples of *Allium chinense* G. Don and stored at 2–8 °C in the Plant Specimen Bank of the First Affiliated Hospital of Zhengzhou University (specimen number ACGD-20210201). A total of 18 authentic standards were isolated and purified in our laboratory, and their structures were subsequently identified using ^1^H and ^13^C nuclear magnetic resonance (NMR) spectroscopy (19, 20), with the purity shown in Table S1. The MEG-01 cell line was obtained from Guandao Bioengineering (Shanghai, China). RPMI-1640 culture medium and fetal bovine serum (FBS, #A5256701) were purchased from Gibco Life Sciences (CA, USA). The Cell Counting Kit-8 (CCK-8, #C0037) was purchased from Beyotime Biotechnology (Shanghai, China). Antibodies against phosphatidylinositol-3 kinase (PI3K, #AF6242) and phosphorylated PI3K (*p*-PI3K, #AF3242) were purchased from Affinity Biosciences (Melbourne, Australia). Antibodies against protein kinase B (AKT, #9272S) and phosphorylated AKT (*p*-AKT, #9271S) were obtained from Cell Signaling Technology (MA, USA). Acetonitrile and methanol were of LC–MS grade, whereas all other chemical reagents were of analytical grade.

### Sample preparation

The pulverized *Allium chinense* G. Don samples (1.0 g) were individually extracted with 10 mL of deionized water and 30, 50, 70%, or 100% methanol via ultrasonication for 30 min. The resulting suspensions were centrifuged at 10,000 × g for 5 min to collect the supernatants. The supernatants were dried in a vacuum centrifuge concentrator at 4 °C. The dried samples (1 mg) were weighed and dissolved in phosphate-buffered saline (PBS) solution at 1 mg/mL. The sample solutions were filtered through a microfiltration membrane (0.22 μm) and injected into the LC–MS system.

### Platelet aggregation–inhibitory activity

In this study, human platelet aggregation experiments were performed to evaluate the platelet aggregation–inhibitory activity of different ACGD extracts. The platelet aggregation rate was measured using light transmission aggregometry (4). Briefly, human blood samples were collected in vacuum tubes containing sodium citrate as an anticoagulant. Platelet-rich plasma (PRP) and platelet-poor plasma (PPP) were obtained after differential centrifugation. The aggregation rate was determined using an AG800 automated platelet aggregometer (Shandong, China). The light transmittance of the PPP fraction was calibrated to 100% to establish a baseline. AA, ADP, and collagen were set as positive controls, while ACGD samples extracted using different solvents (1 mg/mL) were added to PRP samples to measure their respective transmittances. The control group was treated with an equal volume of PBS solution, and the other procedures were consistent with the experimental groups. Platelet aggregation inhibitory effects were evaluated based on the maximum aggregation rate (%) and corresponding aggregation curves. This experimental plan was approved by the Ethics Committee of the First Affiliated Hospital of Zhengzhou University (2021-KY-0217), and informed consent was obtained from the subjects.

### Data-dependent acquisition by LC–MS

ACGD samples were analyzed using an ACQUITY H-Class ultra-performance liquid chromatography (UPLC) system (Waters, Milford, MA, USA). A BEH C18 column (2.1 × 50 mm, 1.7 μm) was used to separate the samples at 40 °C. Water (A) and acetonitrile (B), both containing 0.1% formic acid, were used as the mobile phases. The gradient elution program was as follows: 0–2 min, 2–4% B; 2–13 min, 4–10% B; 13–23 min, 10–40% B; 23–24 min, 40–45% B; 24–25 min, 45% B; 25–27 min, 45% B. The flow rate was set at 0.3 mL/min, and the injection volume was 5 μL.

The UPLC system was connected to a Q-Exactive-Orbitrap-MS system using electrospray ionization (ESI). The MS parameters in the positive (ESI+) and negative (ESI-) ion modes were as follows. The spray voltage was 3.5 kV (ESI+) and 2.5 kV (ESI-), respectively. The capillary temperatures were 320 °C (ESI+) and 380 °C (ESI-), respectively. The heater temperatures were 400 °C (ESI+) and 350 °C (ESI-), respectively. The sheath gas pressure and auxiliary gas pressures were 35 and 10 arbs (ESI+) and 40 and 5 arbs (ESI-), respectively. The ranges for the MS^1^ and MS^2^ scans in the positive and negative ion modes were both *m/z* 50–1,500 and 50–1,500 Da. The full MS-DD-MS^2^ scan mode was used to collect the MS data. Under these scan modes, the detected precursor ions (the top five abundant) were selected to collect their high-quality MS^2^ spectra. Xcalibur software was used to process the obtained data.

### Molecular networking analysis

Feature-based molecular networking (FBMN) analysis of ACGD samples using the GNPS platform was performed based on a previous study (15, 16). Briefly, the original LC–MS raw data were converted to a list of different features (mzML format) using the MZmine 3 (https://mzmine.github.io/) platform. These features were detected, integrated, filtered, and visualized to generate an FBMN diagram. The mass tolerance for precursor ions and fragment ions was 0.01 Da. The minimum number of matched peaks was five, and the cosine score was 0.6. Cytoscape 3.9.1 was used to visualize the FBMN.

Structure annotation.

The obtained spectra were matched with the GNPS spectral library. The mass tolerance was set to 0.01 Da, requiring all matched candidates to have a mass error of less than 0.01 Da. The adduct ions contained [M + H-H_2_O]^+^, [M + H]+, [M + Na]+, [M-H-acetyl]-, [M-H]-, and [M-H + HCOOH]-. The molecular formulae of the principal sapogenins in ACGD were C_27_H_44_O_3_, C_27_H_44_O_4_, and C_27_H_42_O_4_. Furthermore, the primary neutral losses observed included C_6_H_10_O_5_ (corresponding to glucose or galactose), C_5_H_8_O_4_ (xylose or arabinose), and C_2_H_2_O (acetyl group).

### Network pharmacology analysis

The SwissTargetPrediction database (http://www.swisstargetprediction.ch/) was used to predict the putative targets of the selected ACGD components (21, 22), with the target species restricted to *Homo sapiens*. Known therapeutic targets associated with coronary heart disease were retrieved from DrugBank (https://www.drugbank.ca/), GeneCards (https://www.genecards.org/), and OMIM (http://omim.org/) databases. The potential therapeutic targets of ACGD-related components for treating coronary heart disease were subsequently determined through intersection analysis. Furthermore, the STRING platform (https://string-db.org/) was employed to construct a protein–protein interaction (PPI) network of these overlapping targets using *Homo sapiens* as the specified organism and applying a high-confidence interaction score threshold of >0.9. Additionally, the Metascape (http://metascape.org) platform was used to generate the Kyoto Encyclopedia of Genes and Genomes (KEGG) pathway enrichment analysis. Finally, Cytoscape 3.9.1 software was used to visualize the constructed pharmacological networks.

### Activity evaluation of ACGD-related components in MEG-01 cells

MEG-01 cells were obtained from Procell Life Science & Technology Co., Ltd. (Wuhan, China). The cells were identified by STR and were found to be free of mycoplasma contamination. MEG-01 cells were used to investigate the effects of saponins on several signaling pathways. MEG-01 cells were cultured in RPMI-1640 medium supplemented with 10% FBS and antibiotics (including 100 U/mL penicillin and 100 μg/mL streptomycin) in an incubator with 5% CO_2_ at 37 °C (23). Cells at a density of 2 × 10^4^ cells/well were plated into 96-well plates. ADP (positive control) and different concentrations of ACGD-related components (5, 10, and 20 μM) were added to these cells and co-incubated for 24 h. CCK-8 reagent (10 μL) was then added to each well and incubated for 4 h. An EnSight microplate reader (PerkinElmer) was used to measure the optical density at 450 nm, and the cell proliferation inhibition rate was calculated.

Cells were lysed using RIPA buffer supplemented with protease inhibitors to obtain total protein extracts. Denatured protein samples (40 μg per lane) were resolved on 10% SDS-PAGE gels. Following electrophoresis, the separated proteins were transferred onto PVDF membranes. Subsequently, the membranes were blocked with 5% non-fat milk in PBS containing 0.1% Tween 20 for 1 h. Following blocking, the membranes were incubated overnight at 4 °C with primary antibodies against PI3K, *p*-PI3K, AKT, and *p*-AKT. The membranes were washed and incubated with the appropriate secondary antibodies for 1 h. Finally, the protein bands were visualized using an enhanced chemiluminescence (ECL) reagent, and relative intensities were quantified using ImageJ software.

### Statistical analysis

Statistical significance was evaluated using one-way analysis of variance (ANOVA) in GraphPad Prism version 5.0. Differences were considered statistically significant at *p* < 0.05.

## Results

### Analytical procedure

First, untargeted LC–MS analyses of ACGD samples extracted with various solvents were performed, along with the evaluation of their platelet aggregation–inhibitory activities. All LC–MS data were acquired in data-dependent acquisition (DDA) mode. Second, a feature-based molecular networking (FBMN) analysis of the 70% methanol extract of ACGD was generated using the MZmine 3 and GNPS platforms. Concurrently, the fragmentation patterns of each type of steroidal saponin in ACGD were summarized based on their respective diagnostic ions. The chemical components in ACGD were subsequently identified or tentatively characterized based on structural annotations from FBMN and specific fragmentation characteristics. Third, based on the previously reported platelet inhibitory activities of ACGD-related components, several representative compounds were selected for network pharmacology analysis. The PI3K/Akt signaling pathway emerged as a core mechanism underlying the inhibition of platelet aggregation. The expression of key proteins in this pathway was evaluated in MEG-01 cells. Taken together, this integrated strategy was successfully established to elucidate the mechanisms by which these active components in ACGD inhibit platelet aggregation through the PI3K/Akt signaling pathway.

### Activity-labeled molecular networking analysis

**Figure 1:**
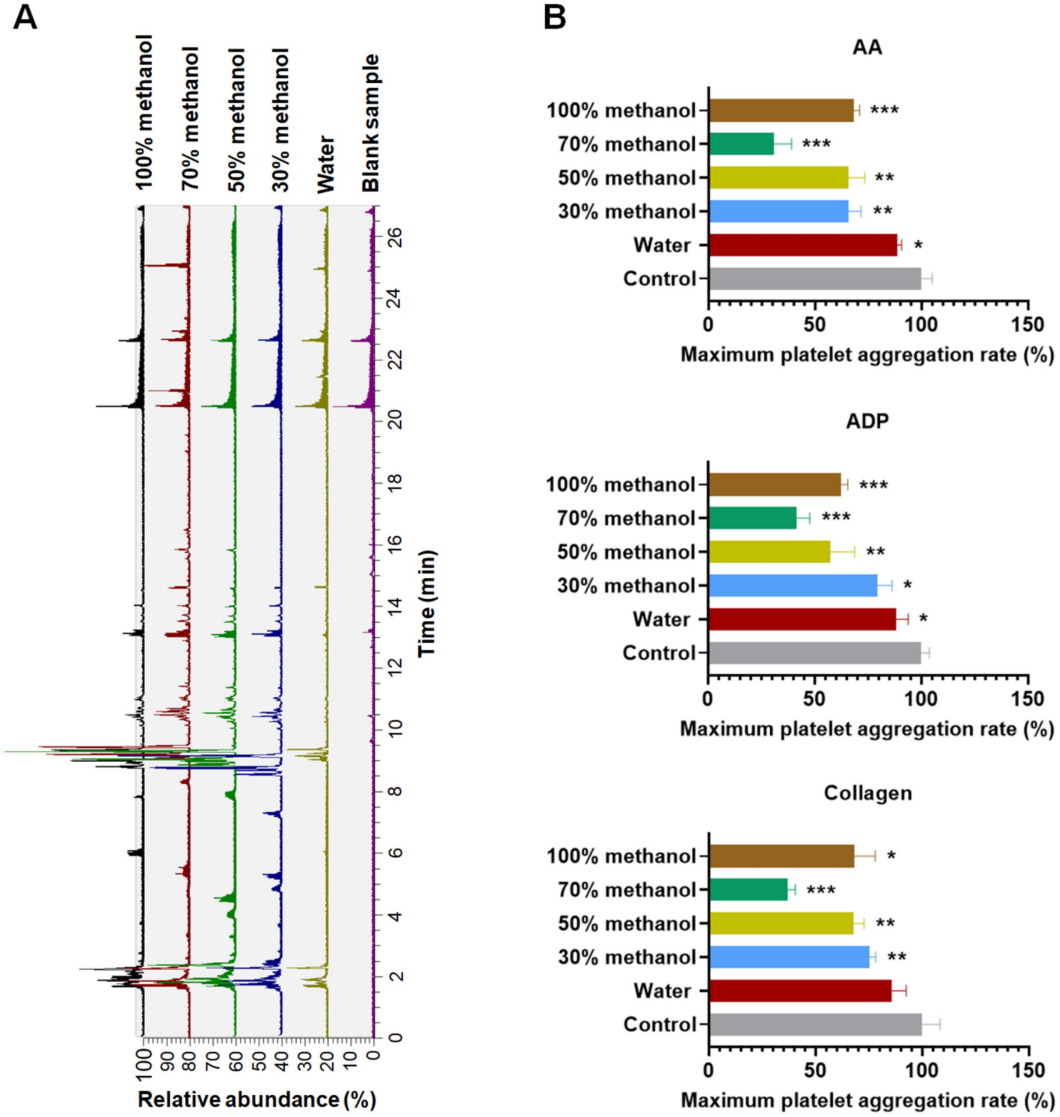
Spectrum–activity difference analysis of the ACGD samples. **(A)** LC–MS profiles of ACGD samples extracted using different solvents, including water, 30% methanol, 50% methanol, 70% methanol, and 100% methanol. **(B)** Platelet aggregation–inhibitory activity of different ACGD extracts (1 mg/mL) against arachidonic acid (AA), adenosine diphosphate (ADP), and collagen-induced platelet aggregation. (*n* = 3; one-way ANOVA; * compared with the control group, *p* < 0.05).

As illustrated in Figure 1A, the total ion chromatograms (TICs) of the ACGD samples extracted with water and 30, 50, 70, and 100% methanol were acquired using an LC-Q-Exactive-Orbitrap-MS system. Only minor differences were observed between the chromatograms. AA, ADP, and collagen were used as positive controls to induce platelet aggregation (Figure 1B). Notably, ACGD samples extracted with different solvents all exhibited significant antiplatelet activity (*p* < 0.05) in AA-, ADP-, and collagen-induced human platelet aggregation (Supplementary Figures S1–S6). Among these, the 70% methanol extract of ACGD demonstrated the most potent antiplatelet aggregation activity (Figure 1B). Consequently, the identification of potential bioactive constituents focused on this highly active fraction.

**Figure 2:**
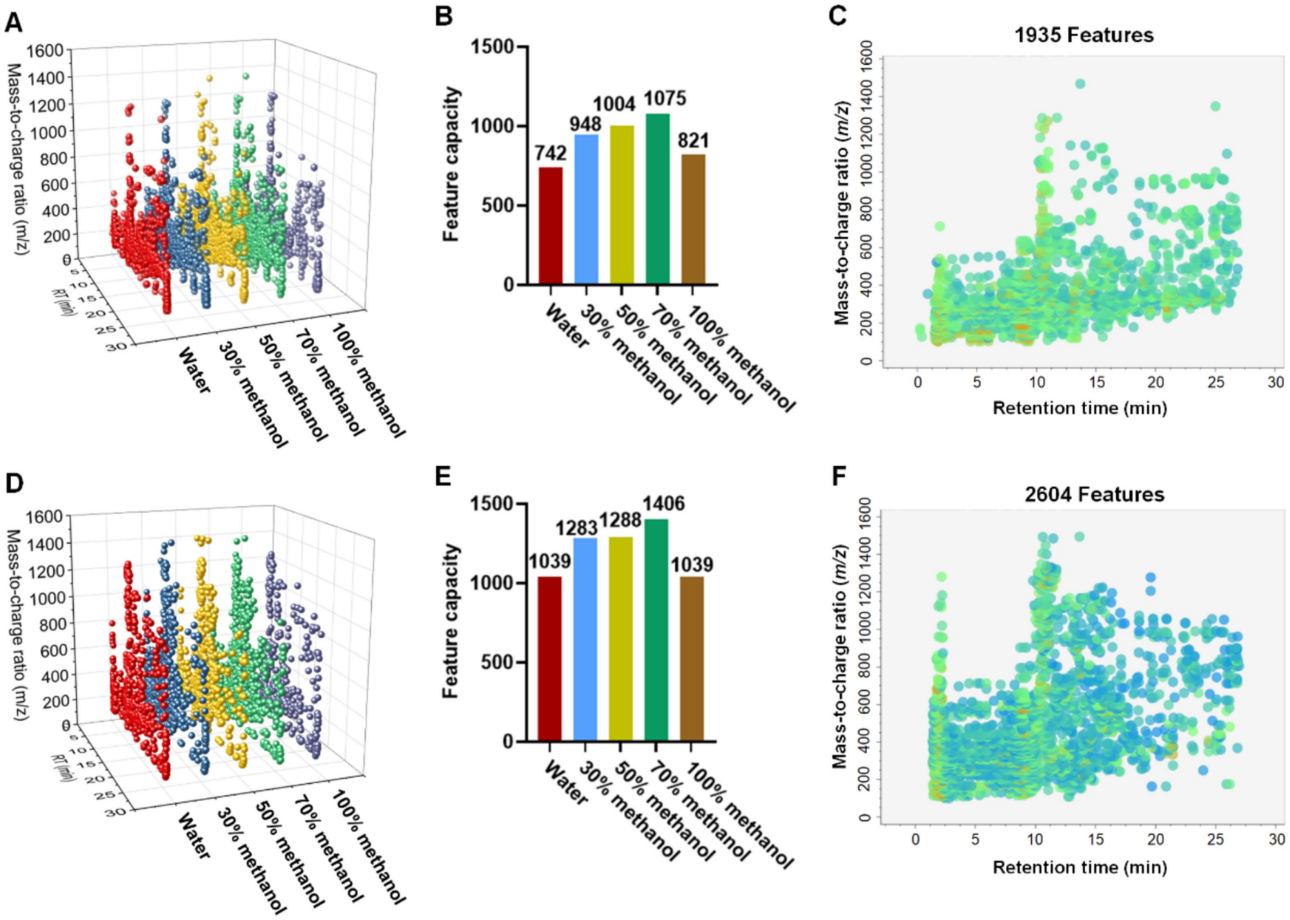
Ion feature analysis of ACGD samples extracted using different solvents. Distribution of ion features (*m/z* vs. retention time) across ACGD samples extracted with different extraction solvents in positive **(A)** and negative **(D)** ion modes. Feature counts after blank subtraction in positive **(B)** and negative **(E)** ion modes. Total ion scatter plots processed by MS-DIAL in positive **(C)** and negative **(F)** ion modes.

Figure 2 illustrates the comprehensive profiling of the chemical features across the five ACGD extraction fractions. The 3D scatter plots (Figures 2A,D) show the distribution of potential precursor ions across different solvent extracts in both the positive and negative ion modes. As shown in the bar graphs (Figures 2B,E), the 70% methanol extract exhibited the highest feature count, with 1,075 and 1,406 features detected in the positive and negative modes, respectively. To refine the dataset, a blank solvent was utilized as a reference to exclude background noise, followed by the alignment and deconvolution of features across all samples. Using a mass tolerance of 0.01 Da and a retention time tolerance of 0.05 min, overlapping components were merged and deduplicated. This process yielded 1,935 (positive mode) and 2,604 (negative mode) unique potential precursor ions, as visualized in the global feature maps (Figures 2C,F). These precursor ions formed the basis for the subsequent FBMN analysis (Supplementary Figure S7). Molecular clusters containing more than three nodes warrant particular attention. However, manual inspection and individual analysis of such a vast number of clustering networks represent a challenge.

**Figure 3:**
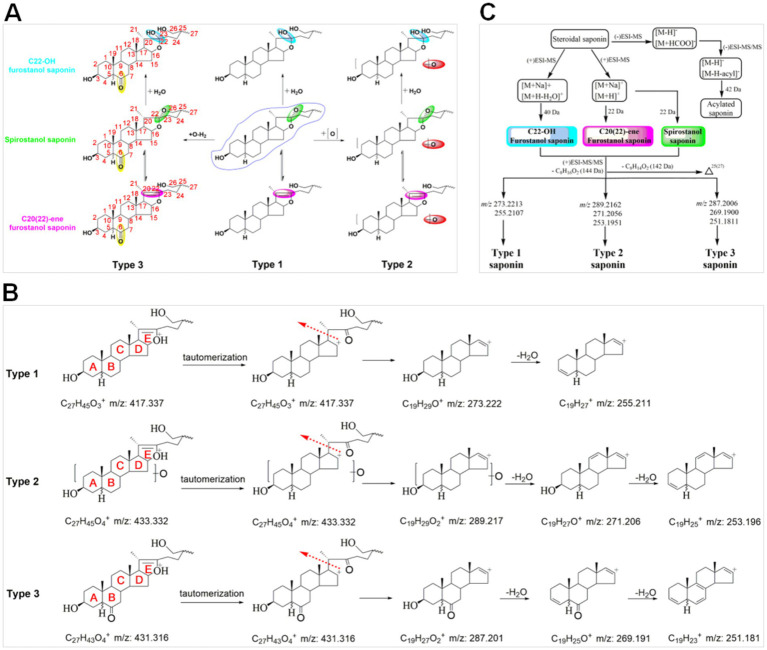
Proposed MS fragmentation patterns of steroidal saponins in ACGD. **(A)** Structural descriptions of different types of steroidal saponins, including C22-OH furostanol saponins, C20(22)-ene furostanol saponins, and spirostanol saponins; **(B)** Diagnostic ions for type 1, type 2, and type 3 saponins in positive ion mode; **(C)** Summary of proposed fragmentation patterns of multiple types of steroidal saponins.

Therefore, a targeted molecular networking strategy was established to efficiently identify the characteristic compounds in ACGD through a data-redundancy filtering method. Three types of steroidal saponins were identified as the primary characteristic components of ACGD. Their sapogenins undergo mutual transformations via various structural modifications, including (+O, +15.9949 Da), hydration (+H_2_O, +18.0106 Da), hydroxylation, and reduction (+O-2 × H, +13.9792 Da) (Figure 3A). The carbohydrate chain mainly consisted of glucose (C_6_H_10_O_5_, 162.0528 Da), galactose (C_6_H_10_O_5_, 162.0528 Da), arabinose (C_5_H_8_O_4_, 132.0423 Da), and xylose (C_5_H_8_O_4_, 132.0423 Da). Other modifications, such as acetylation (C_2_H_2_O, 42.0106 Da), have also been observed in certain steroidal saponins. Second, using the sapogenin core (C_27_H_44_O_3_) as the structural scaffold, potential derivatives were predicted based on substituents such as hydroxyl, hydrate, glycosyl, and acetyl groups. Consequently, 36 precursor ions were successfully targeted for structural annotation.

### Fragmentation patterns and structural annotation of target saponins

In this study, the target steroidal saponins were classified into three distinct types based on different sapogenins (Figure 3A). In negative ion mode, [M-H]^−^, [M-H + HCOOH]^−^, and [M-H-acetyl]^−^ ions were clearly detected in the MS^1^ spectra of the acetylated saponins. For spirostanol and furostanol saponins with a C20–C22 double bond, [M + H]^+^ and [M + Na]^+^ served as the primary precursor ions. [M + Na]^+^ and [M + H-H_2_O]^+^ ions characterized furostanol saponins hydroxylated at the C-22 position. In addition, the different types of sapogenins exhibited many similar fragmentation patterns in the positive MS^2^ spectra (Figure 3B). Neutral losses and E-ring cleavage were the main fragmentation modes of these saponins. For example, neutral losses of monosaccharide units (mainly 132.0423 and 162.0528 Da) and acetyl groups (42.0106 Da) were observed across these saponins. The presence of the diagnostic fragment at *m/z* 144.1150 (C_8_H_16_O_2_) indicated E-ring cleavage across all three types of sapogenins (Figure 3B). Interestingly, the appearance of the fragment at *m/z* 142.0994 (C_8_H_14_O_2_) suggested the presence of a double bond between the C-25 and C-27 positions. Furthermore, diagnostic ions have been proposed to differentiate among the various types of saponins in ACGD. The ions at *m/z* 273.222 and 255.211 were identified as diagnostic fragments of type 1 saponins, whereas those with fragment ions at *m/z* 289.217, 271.206, and 253.196 were identified as type 2 saponins. Type 3 saponins, the most abundant components in ACGD, yielded diagnostic ions at *m/z* 287.201, 269.191, and 251.181 (Figure 3C). Based on these fragmentation patterns, the structural annotation of the 36 precursor ions derived from the targeted molecular networking analysis was performed as follows.

**Figure 4:**
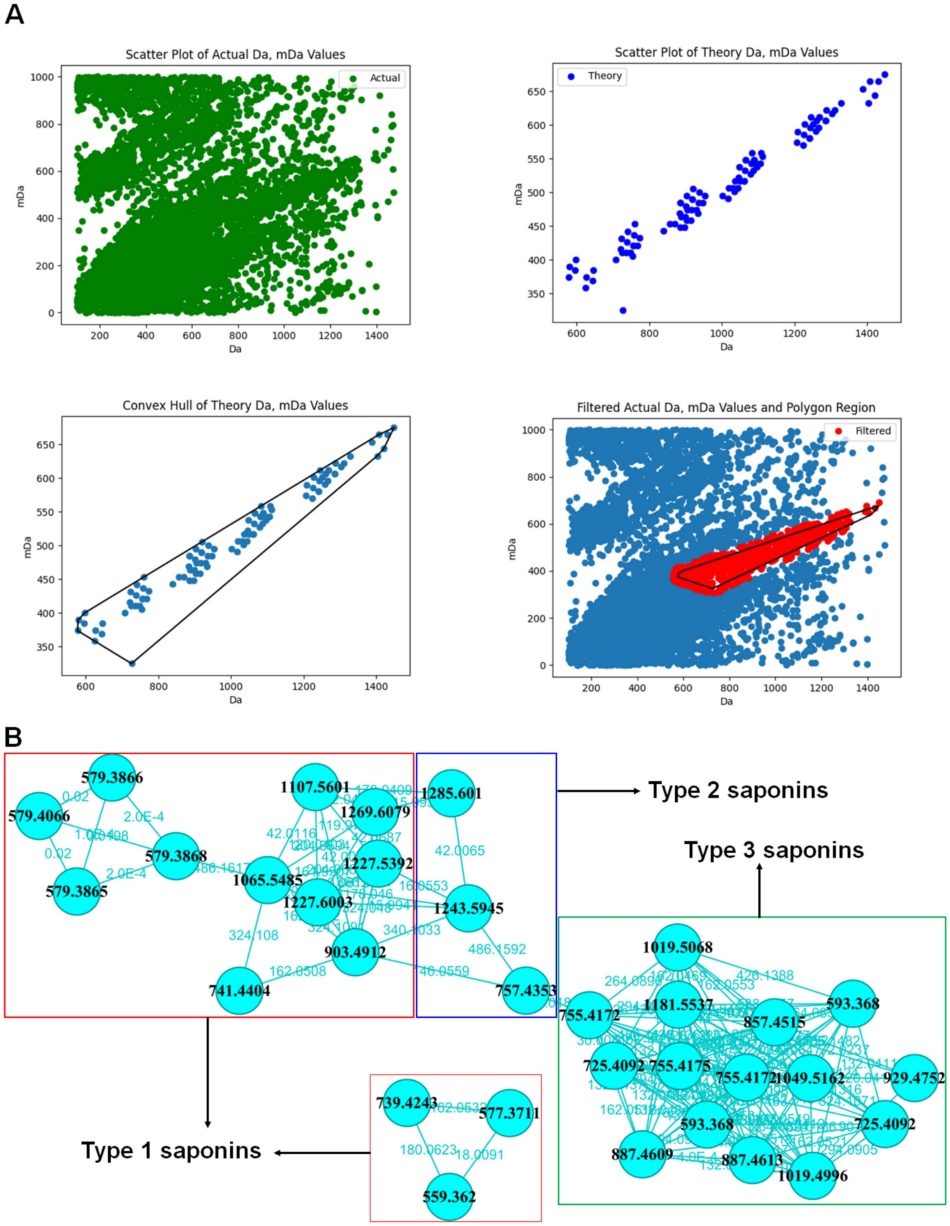
Targeted molecular networking analysis of the ACGD sample extracted with 70% methanol. **(A)** Mass defect filtering (MDF) of steroidal saponins in the ACGD samples. **(B)** Annotation of steroidal saponins *via* targeted molecular networking.

**Figure 5:**
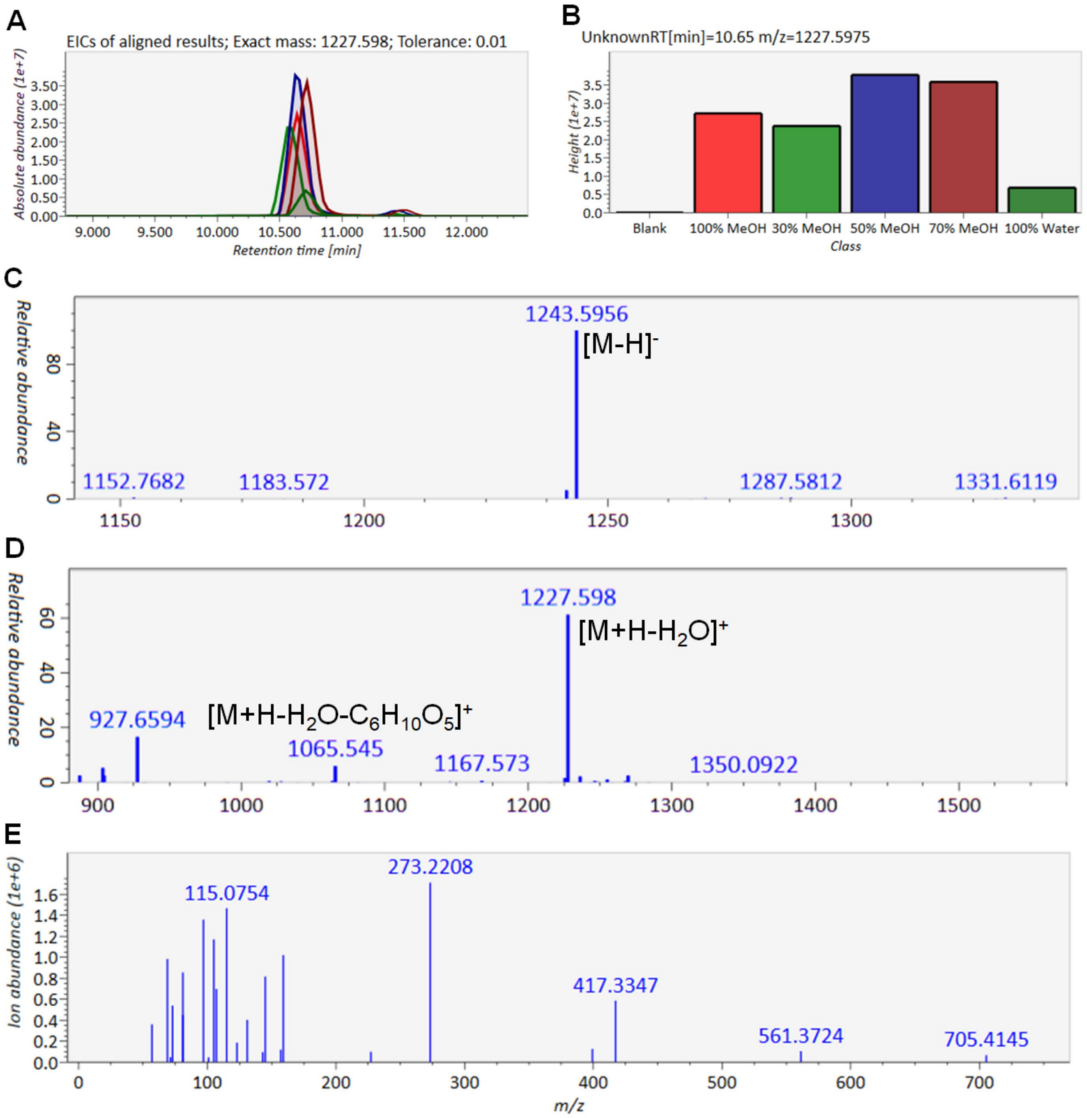
MS and MS^2^ data for T1-1 saponin in the ACGD sample. **(A)** Extracted ion chromatogram of *m/z* 1227.5980 at 10.65 min. **(B)** Comparison of T1-1 peak heights treated with different ACGD extracts. **(C)** MS chromatogram of T1-1 in the negative ion mode. **(D)** The MS chromatogram of T1-1 in the positive ion mode. **(E)** MS^2^ chromatogram of T1-1 in the positive ion mode. Annotation suggests the proposed fragmentation mode.

**Figure 6:**
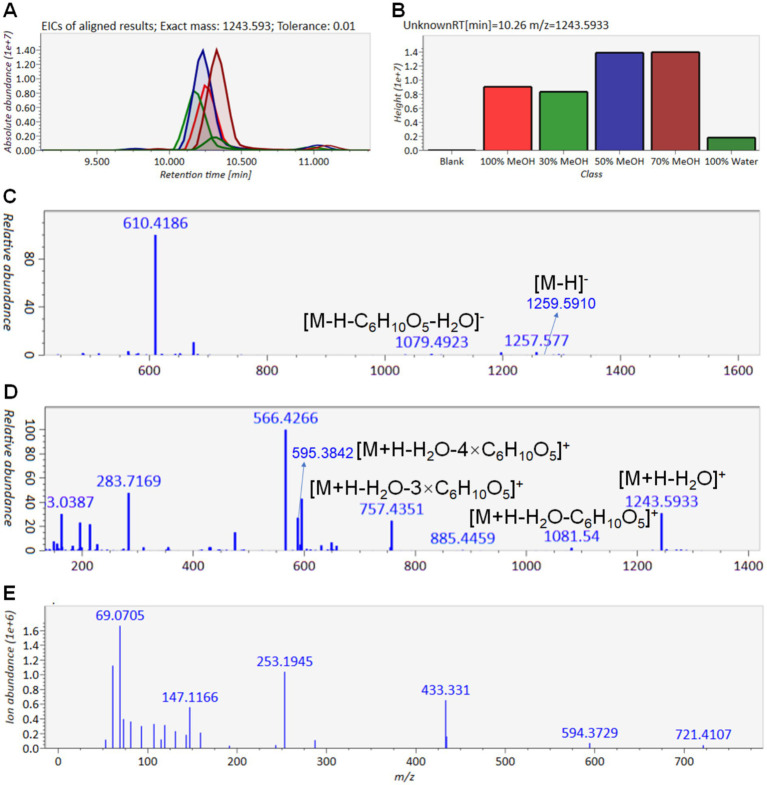
MS and MS^2^ information of T2-1 saponin. **(A)** Extracted ion chromatogram of *m/z* 1243.5930 at 10.26 min. **(B)** Height of T2-1 in different ACGD extracts. **(C)** MS chromatogram of T2-1 in the negative ion mode. **(D)** MS chromatogram of T2-1 in positive ion mode. **(E)** MS^2^ chromatogram of T2-1 in the positive ion mode. Annotation explains the proposed fragmentation forms of T2-1.

Following data redundancy filtering, a refined dataset was obtained, as illustrated in Figure 4A. The corresponding targeted molecular network is shown in Figure 4B. Based on the MS^1^ and MS^2^ spectral data, the 36 precursor ions were categorized into different types of saponins present in ACGD. For compound T1-1, the extracted ion chromatograms (EICs) at *m/z* 1227.598 across ACGD samples extracted with various solvents are displayed in Figure 5A, and the corresponding peak heights are shown in Figure 5B. In the negative ion mode, the ion at *m/z* 1243.5956 was detected as the [M-H]^−^ ion (Figure 5C). In the positive ion mode, [M + Na]^+^ and [M + H-H_2_O]^+^ ions were observed at *m/z* 1267.5940 and 1227.5980, respectively (Figure 5D). The fragment ions at *m/z* 1065.5450 and 903.4937 likely originated from the in-source neutral losses of the precursor ion at *m/z* 1227.5980 (Figure 5D). In Figure 5E, the diagnostic ion at *m/z* 273.2208 is clearly observed, indicating that an E-ring cleavage occurred from the related ion at *m/z* 417.3347. This indicates that compound T1-1 is a type 1 saponin. Based on its retention behavior and mass spectral data compared with an authentic standard (Supplementary Figure S8), T1-1 was unambiguously identified as Macrostemonoside B. Similarly, the visualized EIC images of T2-1 are shown in Figures 6A,B. The precursor ions at 1259.5910 (Figure 6C), 1283.5830, and 1243.5930 (Figure 6D) were defined as the [M-H]^−^, [M + Na]^+,^ and [M + H-H_2_O]^+^ ions of T2-1 saponin, respectively. The series of ions mainly originated from neutral losses of the [M + H-H_2_O]^+^ ion. The ions at *m/z* 1081.5419, 757.4351, and 595.3842 were considered [M + H-H_2_O-C_6_H_10_O_5_]^+^, [M + H-H_2_O-3 × C_6_H_10_O_5_]^+^, and [M + H-H_2_O-4 × C_6_H_10_O_5_]^+^ ions, respectively. In Figure 6E, the diagnostic ions at *m/z* 289.2136 and 253.1945 were attributed to the E-ring cleavage of the ion at *m/z* 433.3310, suggesting that compound T2-1 was a type 2 saponin. Therefore, it was identified as Macrostemonoside R through comparison with a reference standard.

Type 3 saponins were the most abundant in ACGD. The extracted ion chromatogram (EIC) and peak heights for the ion at *m/z* 1019.5030 are shown in Figures 7A,B. Furthermore, the negative ESI-MS spectra yielded the [M-H]^−^ and [M-H + HCOOH]^−^ ions at *m/z* 1035.5020 and 1081.5068, respectively (Figure 7C). Conversely, in the positive ion mode, the MS^1^ spectra exhibited the [M + H-H_2_O]^+^ ion at *m/z* 1019.5030, alongside two neutral loss fragment ions at *m/z* 887.4611 ([M + H-H_2_O-C_5_H_8_O_4_]^+^) and 593.3669 ([M + H-H_2_O-2 × C_5_H_8_O_4_-C_6_H_10_O_5_]^+^) (Figure 7D). The observation of a diagnostic ion at *m/z* 269.1894 from E-ring cleavage of the ions at *m/z* 431.3148 and 413.3048 strongly suggested that T3-3 is a type 3 saponin (Figure 7E). By comparing its spectral data with an authentic standard, T3-3 was unambiguously identified as Chinenoside I. Similarly, the retention behaviors and MS information of other type 1 (Supplementary Figures S9–S18), type 2 (Supplementary Figures S19–S24), and type 3 (Supplementary Figures S25–S41) saponins are provided to support their structural characterization. Detailed information and structures of these steroidal saponins are shown in Table 1 and Figure 8.

**Figure 7:**
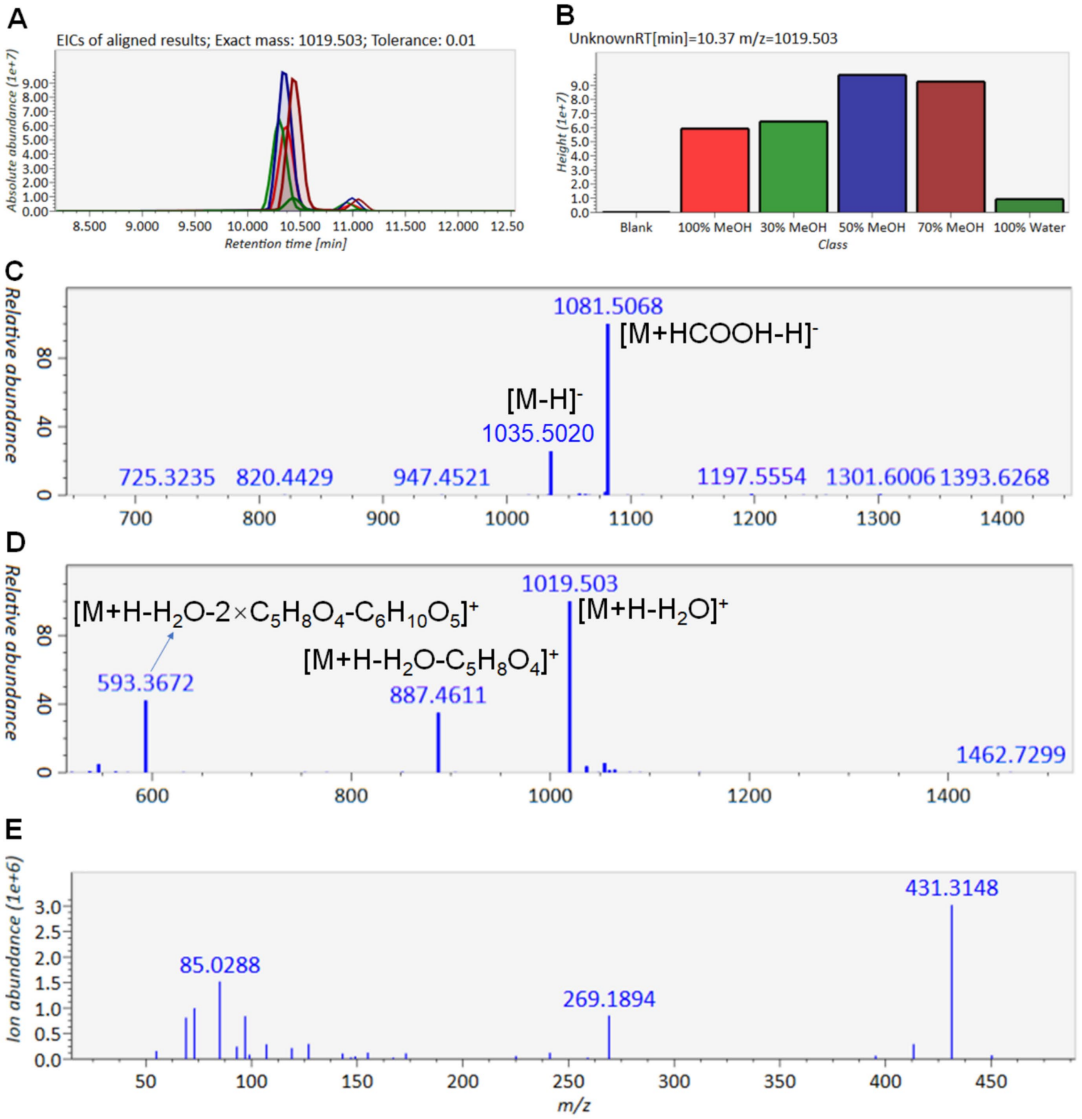
Detailed MS and MS^2^ data for T3-3 saponin. **(A)** Extracted ion chromatogram of *m/z* 1019.5030 at 10.37 min. **(B)** Height analysis of T3-3 in different ACGD extracts. **(C)** (−) ESI-MS chromatogram of T3-3. **(D)** (+) ESI-MS chromatogram of T3-3. **(E)** (+) ESI-MS^2^ chromatogram of T3-3. The proposed fragmentation mode is presented in the annotation section.

**Table 1:** Detailed information on identified or tentatively characterized saponins from ACGD by UPLC–MS.

No.	RT (min)	Ion (*m/z*)	Ion type	(−) ESI-MS/MS	(+) ESI-MS/MS	Identification and characterization
Type 1 saponins						
^#^T1-1	10.72	1227.5980	[M + H-H_2_O]^+^	1243.5956	1267.5940, 1227.5980, 1065.5450, 741.4401, 579.3880, 417.3347, 399.3124 273.2208	Macrostemonoside B
T1-2	10.75	1065.5450	[M + H-H_2_O]^+^	1081.5458	1105.5290, 1065.5460, 903.4937, 741.4407, 579.3886, 417.3356, 255.2103	(25*R*)-26-O-β-D-glucopyranosyl-5*α*-furost-3*β*,26-didyroxy-3-O-{O-β-D-glucopyranosyl-(1 → 2)-O-β-D-glucopyranosyl-(1 → 4)-β-D-galactopyranoside}
^#^T1-3	10.92	903.4927	[M + H-H_2_O]^+^	965.4975, 919.4916	943.4470, 903.4927, 741.4403, 579.3903, 417.3346, 255.2104	(25R)-26-O-β-D-glucopyranosyl-5α-furost-3β,26-didyroxy-3-O-{O-β-D-glucopyranosyl-(1 → 4)-β-D-galactopyranoside}
T1-4	10.99	1269.6080	[M + H-H_2_O]^+^	1331.6127, 1285.6060 1225.5500	1309.6020, 1269.6085, 1107.5559, 741.4398, 579.3878, 417.3355, 255.2104	Acetyl-Macrostemonoside B
T1-5	11.05	1107.5560	[M + H-H_2_O]^+^	1123.5199	1147.5360, 1107.5553, 741.4406, 579.3875, 561.3757, 435.2732, 417.3352, 255.2103	Acetyl-(25R)-26-O-β-D-glucopyranosyl-5α-furost-3β,26-didyroxy-3-O-{O-β-D-glucopyranosyl-(1 → 2)-O-β-D-glucopyranosyl-(1 → 4)-β-D-galactopyranoside}
^#^T1-6	11.49	1227.5990	[M + H]^+^	1225.5862	1227.5990, 1065.5483, 903.4931, 417.3349, 273.2206	Macrostemonoside E
T1-7	11.52	1065.5450	[M + H]^+^	1109.5392, 1063.5394	1087.5280, 1065.5470, 903.4929, 417.3353, 273.2206	Macrostemonoside E-glc
T1-8	11.76	1269.6100	[M + H]^+^	1267.5922	1291.5910, 1269.6100, 1107.5560, 741.4404, 579.3884, 417.3353, 273.2206	Acetyl-Macrostemonoside E
^#^T1-9	14.63	1065.5460	[M + H]^+^	1063.5358	1087.5270, 1065.5437, 741.4399, 579.3881, 417.3352, 255.2101	Macrostemonoside A
^#^T1-10	15.19	903.4940	[M + H]^+^	947.4876, 901.4822	925.4752, 903.4940, 561.3754 417.3350, 399.3165, 255.2102	(25S)-5α-spirostane-3β-ol-3-O-{O-β-D-glucopyranosyl-(1 → 2)-O-β-D-glucopyranosyl-(1 → 4)-β-D-galactopyranoside}
^#^T1-11	15.56	1107.5560	[M + H]^+^	1105.5458, 1063.4946	1129.5380, 1107.5560, 741.4398, 579.3882, 417.3350, 273.2206	Macrostemonoside D
Type 2 saponins						
^#^T2-1	10.33	1243.5930	[M + H-H_2_O]^+^	1259.5910, 1079.4923 449.3271	1283.5830, 1243.5930, 1081.5419, 919.4876, 757.4351, 595.3842, 433.3323, 289.2136, 253.1945	Macrostemonoside R
T2-2	10.52	1285.6040	[M + H-H_2_O]^+^	1301.6058	1325.5990, 1285.6040, 757.4349, 595.3827, 433.3310, 253.1945	Acetyl-Macrostemonoside R
T2-3	11.09	1243.5940	[M + H]^+^	1287.5810, 1241.5813 1223.5693	1265.5680, 1243.5941, 919.4873, 757.4356, 595.3833, 415.3195, 253.1942	20(22)-ene-Macrostemonoside R
T2-4	12.63	919.4885	[M + H]^+^	963.4821, 917.4764	941.4703, 919.4885, 595.3826, 451.2680, 433.3308, 253.1944	Macrostemonoside L
T2-5	13.57	1081.5410	[M + H]^+^	1079.4932	1081.5410, 919.4875, 757.4354, 595.3831, 559.3597, 433.3303, 253.1947	2α-OH-Macrostemonoside A
^#^T2-6	13.84	919.4885	[M + H]^+^	963.4823, 917.4763	919.4885, 757.4343, 595.3832, 415.3198, 397.3092, 271.2048, 253.1959	(25R)-5α-spirostane-2*α*,3β-diol-3-O-{O-β-D-glucopyranosyl-(1 → 2)-O-β-D-glucopyranosyl-(1 → 4)-β-D-galactopyranoside}
^#^T2-7	17.57	433.3214	[M + H]^+^		433.3306, 415.3109, 399.3251, 271.2045, 253.1945	Gitogenin
Type 3 saponins						
T3-1	10.21	1017.4900	[M + H-H_2_O]^+^	1033.489	1057.4780, 1017.4874, 885.4471, 753.4050, 723.3948, 591.3519, 429.2994, 411.2901	25(27)-ene-Chinenoside I
T3-2	10.32	1181.5570	[M + H-H_2_O]^+^	1243.5609, 1197.556 1079.4924, 947.4501	1181.5575, 1049.5146, 887.4608, 755.4199, 593.3669, 575.3583, 431.3149, 413.3052, 287.1999	26-O-β-D-glucopyranosyl-3β,22,26-tridyroxy-25(R)-5α-furostan-6-one-3-O-{O-β-D-glucopyranosyl-(1 → 3)-O-β-D-xylopyranosyl-(1 → 4)-O-[α-L-arabinopyranosyl-(1 → 6)]}-β-D-glucopyranoside
^#^T3-3	10.37	1019.5030	[M + H-H_2_O]^+^	1081.5068, 1035.5020 947.4521	1059.4956, 1019.5030, 887.4611, 857.4506, 755.4194, 725.4091, 593.3669, 431.3148, 413.3048, 269.1894	Chinenoside I
^#^T3-4	10.42	887.4608	[M + H-H_2_O]^+^	949.4657, 903.4598	887.4608, 755.4192, 725.4106, 593.3669, 431.3148, 413.3050, 269.1894	26-O-β-D-glucopyranosyl-3β,22,26-tridyroxy-25(R)-5α-furostan-6-one-3-O-α-L-arabinopyranosyl-(1 → 6)-β-D-glucopyranoside
T3-5	10.48	885.4426	[M + H-H_2_O]^+^	947.4510, 901.4437	885.4459, 753.4018, 723.3939, 591.3518, 429.2998, 411.2885	26-O-β-D-glucopyranosyl 3β,22,26-tridyroxy-25(27)-ene-5α-furostan-6-one-3-O-α-L-arabinopyranosyl-(1 → 6)-β-D-glucopyranoside
T3-6	10.68	1061.5140	[M + H-H_2_O]^+^	1123.5188, 1077.5140 1035.5026	1061.5138, 887.4662, 593.3665, 449.2522, 395.2948, 251.1798	Acetyl-Chinenoside I
T3-7	10.72	929.4725	[M + H-H_2_O]^+^	945.4714, 903.4617	929.4713, 911.4609, 887.4618, 767.4204, 755.4194, 431.3147, 413.3054, 287.2004, 251.1788	Acetyl-26-O-β-D-glucopyranosyl-3β,22,26-tridyroxy-25(R)-5α-furostan-6-one-3-O-α-L-arabinopyranosyl-(1 → 6)-β-D-glucopyranoside
T3-8	10.92	1049.5130	[M + H]^+^	1093.5084, 1047.5038	1049.5129, 887.4612, 755.4195, 593.3671, 431.3145, 287.2000	26-O-β-D-glucopyranosyl-3β,26-dihydroxy-25(*R*)-5α-furostan-20(22)-en-6-one-3-O-{β-D-glucopyranoside-(1 → 4)-O-[α-L-arabinopyranosyl-(1 → 6)]-β-D-glucopyranoside}
^#^T3-9	10.98	1019.5030	[M + H]^+^	1017.492	1019.5034, 887.4612, 725.4089, 593.3672, 413.3041, 287.2001, 269.1892, 251.1790	Chinenoside II
T3-10	11.04	887.4612	[M + H]^+^	885.4509	887.4611, 725.4089, 593.3672, 413.3041, 395.3042, 269.1893, 251.1790	Chinenoside III
T3-11	11.35	929.4720	[M + H]^+^	973.4665, 927.4595	929.4720, 911.469, 887.4609, 413.3042, 395.3052, 251.1795	Acetyl-Chinenoside III
^#^T3-12	13.23	1017.4924	[M-H]^−^	1017.4924	1019.5033, 887.4616, 593.3672, 413.3040, 395.3062, 251.1790	(25R)-3β-hydroxy-5α-spirostan-6-one-3-O-{O-β-D-glucopyranosyl-(1 → 3)-O-β-D-xylopyranosyl-(1 → 4)-O-[α-L-arabinopyranosyl-(1 → 6)]}-β-D-glucopyranoside
T3-13	13.30	887.4616	[M + H]^+^	931.4554, 885.4484	887.4620, 725.4093, 395.2937, 251.1792	Laxogenin-3-O-{β-D-glucopyranoside-(1 → 4)-O-[α-L-arabinopyranosyl-(1 → 6)]-β-D-glucopyranoside}
^#^T3-14	13.48	857.4506	[M + H]^+^	855.438	857.4513, 725.4089, 593.3670, 413.3046, 287.1999, 251.1789	Laxogenin-3-O-{β-D-xylopyranosyl-(1 → 4)-O-[α-L-arabinopyranosyl-(1 → 6)]-β-D-glucopyranoside}
^#^T3-15	13.67	747.3913	[M + Na]^+^	769.4020, 723.3962	747.3913, 725.4087, 431.3146, 395.2930, 251.1786	Laxogenin-3-O-{β-D-xylopyranosyl-(1 → 4)-β-D-glucopyranoside}
^#^T3-16	14.52	723.3970	[M-H]^−^	769.4028, 723.3970	725.4094, 413.3036, 251.1789	Laxogenin-3-O-{α-L-arabinopyranosyl-(1 → 6)-β-D-glucopyranoside}
^#^T3-17	14.98	637.3604	[M + HCOO]^−^	637.3604	615.3492, 593.3671, 431.3147, 413.3029, 287.2000	Laxogenin-3-O-β-D-glucopyranoside
^#^T3-18	19.00	431.3149	[M + H]^+^	/	431.3149, 395.2933, 269.1902	Laxogenin

# Indicates that the compounds were identified using authentic standards. T1, T2, and T3 represent saponins of type 1, type 2, and type 3, respectively.

**Figure 8:**
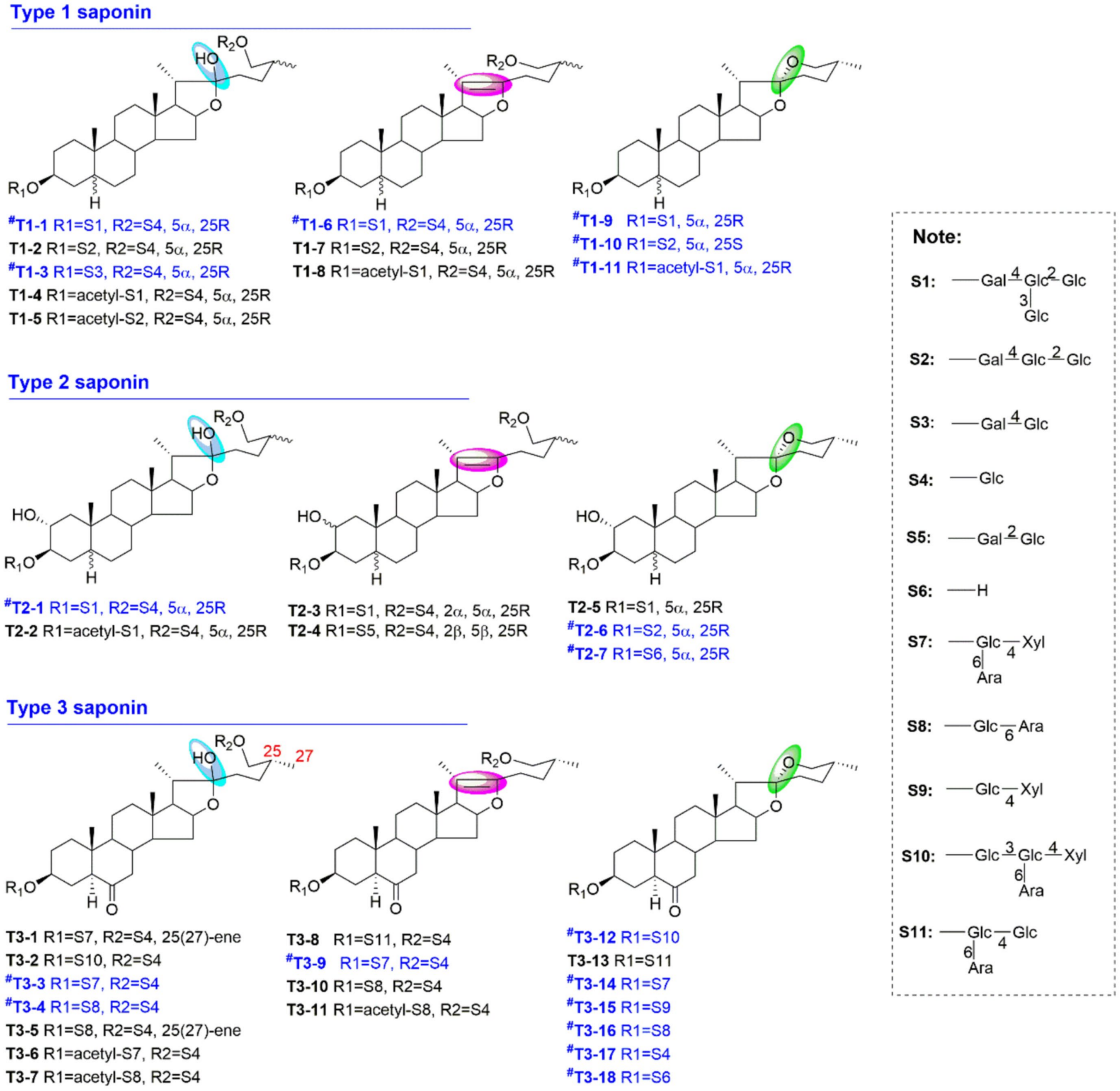
Structures of type 1, type 2, and type 3 saponins identified or tentatively characterized from ACGD samples. Blue annotations indicate that compounds were identified using authentic standards.

### Network pharmacology analysis of representative components

The selection principles for the representative components were as follows. First, these components were reported to have antiplatelet inhibitory activity *in vitro* and/or *in vivo* (4). Second, these components were characteristic and present at high levels in ACGD (20). Third, these components originated from different structural types (19, 20). Fourth, these components can be obtained commercially or isolated from ACGD at >95% purity (19, 20). Therefore, the structures of these components (T1-1, T1-6, T1-9, T1-11, T3-3, T3-4, T3-8, T3-9, T3-10, T3-12, T3-14, T3-15, T3-16, T3-17, and T3-18) were transformed to SMILES format for network pharmacology analysis.

**Figure 9:**
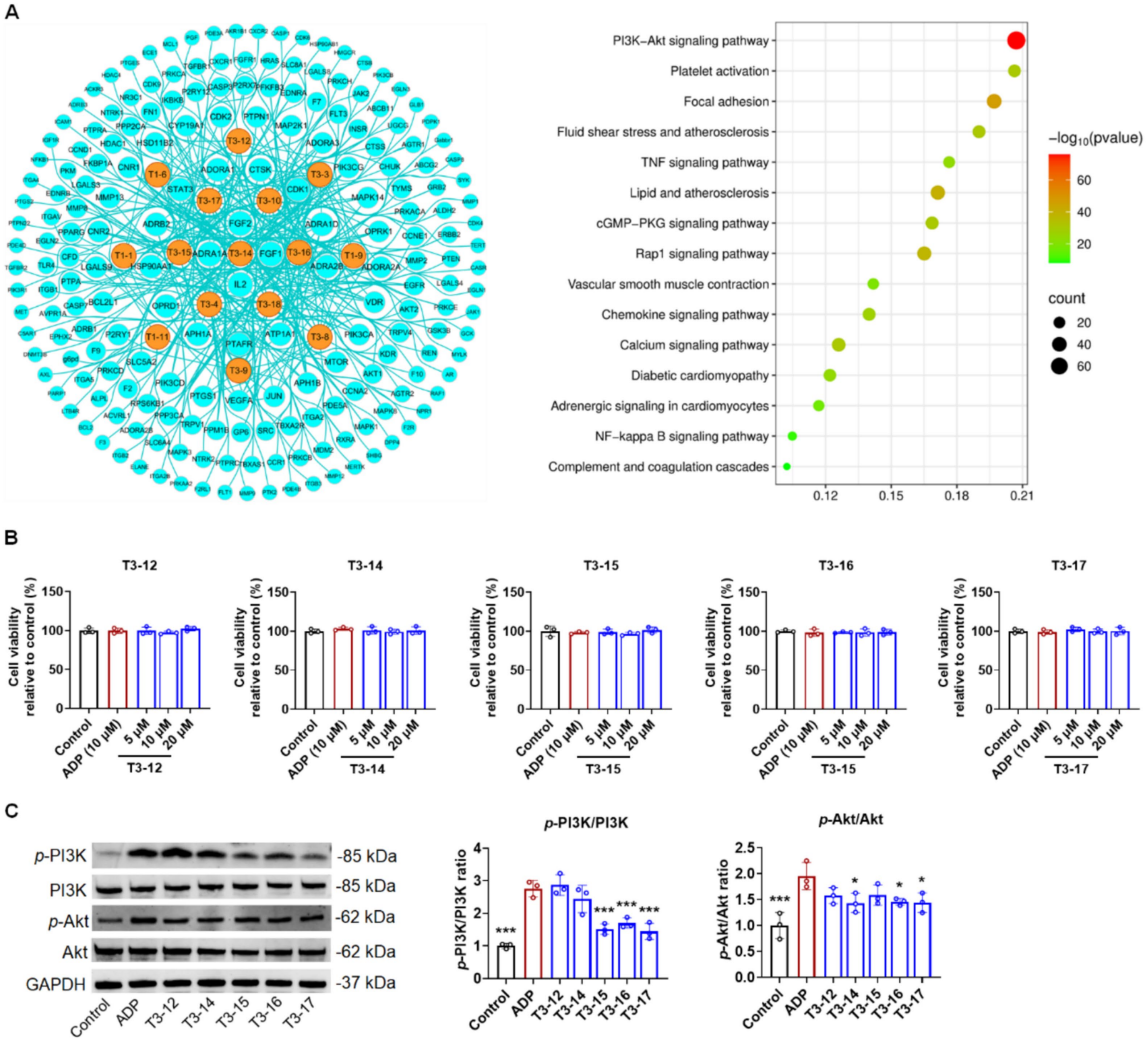
Discovery of antiplatelet aggregation active components from ACGD. **(A)** ACGD-related compound–target network and KEGG pathway enrichment analysis. **(B)** Viability of MEG-01 cells following treatment with different concentrations of T3-12, T3-14, T3-15, T3-16, and T3-17 saponins (5, 10, and 20 μM) was measured using the CCK-8 assay. **(C)** Comparison of relative ratios of *p*-PI3K/PI3K and *p*-AKT/AKT after MEG-01 cells were co-incubated with these five components at 20 μM. (*n* = 3; one-way ANOVA; * compared with those in the control group, *p* < 0.05).

As shown in Figure 9A, many potential targets were associated with these 15 selected components, indicating that they were important potential active components for coronary heart disease. Further KEGG enrichment analysis indicated that some pathways, including platelet activation, fluid shear stress and atherosclerosis, lipid metabolism, and complement and coagulation cascades, were closely associated with coronary heart disease and platelet aggregation (Figure 9A). Among them, the PI3K/Akt signaling pathway was one of the most important (Figure 9A). In this study, we investigated the mechanisms underlying platelet inhibition. Based on our previous results, five main components, including T3-12 (IC_50_ > 100 μM for AA, ADP, and collagen), T3-14 (IC_50_ = 40.5 and 61.4 μM for AA and ADP, respectively), T3-15 (IC_50_ = 53.0, 61.7, and 84.7 μM for AA, ADP, and collagen, respectively), T3-16 (IC_50_ = 25.1 and 60.0 μM for AA and collagen, respectively), and T3-17 (IC_50_ = 35.8, 73.8, and 88.3 μM for AA, ADP, and collagen, respectively), all exhibited significant inhibitory activity on platelet aggregation induced by AA, ADP, and collagen, while the other 10 components demonstrated weak or no antiplatelet aggregation activity (4). Therefore, the effects of these five active components on the PI3K/Akt pathway were further studied.

### Protein expression of the PI3K/Akt pathway in MEG-01 cells

MEG-01 cells were used to evaluate the mechanism of the selected compounds because of their inducible differentiation and partial expression of platelet receptors (23, 24). In this study, the toxicity of T3-12, T3-14, T3-15, T3-16, and T3-17 in MEG-01 cells was investigated at 5–20 μM. The results showed that these components were non-toxic at 5–20 μM and did not induce proliferation of MEG-01 cells within the tested concentration ranges (Figure 9B). ADP was used to induce platelet aggregation as a positive control. ADP significantly increased the elevation of relative expression ratios of *p*-PI3K/PI3K and *p*-AKT/AKT in MEG-01 cells (Figure 9C). It has been reported that the PI3K/Akt pathway is one of the most well-established pathways during platelet aggregation. The active components, including T3-15, T3-16, and T3-17, all dramatically decreased phosphorylation of PI3K, whereas T3-14, T3-16, and T3-17 moderately reduced the *p*-AKT levels (Figure 9C). These findings indicate that T3-16 and T3-17 are the active components in ACGD responsible for inhibiting platelet aggregation through the PI3K/Akt pathway.

## Discussion

Various software platforms, including UNIFI, Progenesis QI, MS-DIAL, and Compound Discoverer, have been widely used to process LC–MS data for the characterization of both targeted and unknown compounds within complex samples (15). However, spectral deconvolution, resolution of overlapping components, and data deduplication remain critical bottlenecks and time-consuming aspects of these conventional approaches (15). To address this, our previously developed targeted molecular networking (TMN) strategy successfully integrated targeted precursor ion scanning, mass defect filtering, neutral loss filtering, and predicted metabolite screening using MS/MS information (18). Furthermore, the visualized annotations and structural relationships of the target compounds within complex matrices were clearly presented using GNPS analysis. This robust TMN strategy has been extensively applied for the characterization of natural products (25–27). In the present study, it was successfully implemented to comprehensively profile the targeted steroidal saponins in ACGD (Table 1). The visualized precursor ion relationship network for these saponins is presented here for the first time (Figure 4B). Notably, our results demonstrated that laxogenin-related saponins (type 3 saponins) are the most abundant steroidal saponins in ACGD (Figure 8).

The saponin fractions from ACGD inhibited the proliferation, migration, and colony formation of B16 and 4 T1 cells in a concentration-dependent manner (28). ACGD saponins also significantly inhibited the growth of melanoma *in vivo* (28). Many purified saponins exhibited significant inhibitory effects on nitric oxide production induced by lipopolysaccharides in RAW264.7 cells and inhibitory activities against the growth of HepG2, A549, CNE-1, MGC80–3, SPC-A-1, and MRC-5 cell lines with IC_50_ values of (3.87 ± 0.22, 10.63 ± 0.96, 6.80 ± 0.56, 4.76 ± 0.29, 15.38 ± 1.06, and 25.17 ± 4.32) μM, respectively, with cisplatin as a positive control (29). In addition, some purified saponins exhibited obvious mitigating effects on hyperlipidemia, hyperglycemia, hypercholesterolemia, and myocardial injury (30, 31). However, few studies have reported the mechanisms underlying the beneficial effects of ACGD. A-24, a saponin from ACGD, induces apoptosis and autophagy via the ROS-PI3K/Akt/mTOR pathway in gastric cancer cells (32, 33). In addition to A-24, there have been no mechanistic studies on other purified saponins, especially those related to cardiovascular diseases.

PI3K is a key survival signaling pathway that can drive the phosphorylation of AKT to modulate cell survival (34). In platelets, activated PI3K can promote the phosphorylation of AKT and further induce platelet activation (24, 35). Many studies have indicated that some natural saponins showed inhibitory effects on platelet aggregation through the PI3K/Akt signaling pathway (36–38). Our network pharmacology analysis results suggested that the mechanism of representative saponins from ACGD mainly focused on the PI3K/Akt pathway (Figure 9A). Further assays confirmed that these saponins exhibited antiplatelet aggregation effects and were non-toxic at 5–20 μM in MEG-01 cells (Figure 9B). ADP, a platelet aggregation agonist, induced the phosphorylation of *p*-PI3K and *p*-Akt (Figure 9C). T3-15, T3-16, and T3-17 decreased the *p*-PI3K/PI3K ratio, whereas T3-14, T3-16, and T3-17 reduced *p*-Akt protein expression (Figure 9C). In addition, T3-14 did not change the ratio of *p*-PI3K/PI3K but lowered the ratio of *p*-AKT/AKT (Figure 9C), suggesting that the downregulation of the PI3K pathway by T3-14 may be independent of PI3K phosphorylation. These findings indicate that the antiplatelet aggregation activity of these saponins increased as the sugar chains at the C-3 position shortened.

One important issue is that these active saponins are spirostanol saponins, which have potential hemolytic characteristics that severely limit their application (3, 39). In this study, the hemolytic activity of these five components was not examined, which represents one limitation of this study. Based on our previous study, spirostanol saponins have a certain level of exposure in the body (13). Addressing the potential hemolytic effects of these components remains a challenge for future research. Prodrug strategies and precise structural modifications are chemical strategies to address the limitations associated with their molecular structure of spirostanol saponins (40). In addition, enteric-coated formulations could ensure that spirostanol saponins are not released in the stomach’s acidic environment but are instead released and absorbed after entering the intestine (41). A new drug delivery system allows these components to avoid direct contact with the red blood cell membrane during blood circulation and to be released after reaching the target site (42). For example, the prepared dioscin-cholesterol nano-complex could significantly decrease hemolysis and organ toxicity and retain antitumor activity in mice (43).

## Conclusion

In summary, the 70% methanol extract of ACGD exhibited the most potent inhibitory effects on platelet aggregation induced by AA, ADP, and collagen compared to the other extraction fractions. Furthermore, our established TMN strategy was successfully applied to identify and visualize the characteristic steroidal saponins within ACGD. Based on retention time and fragmentation patterns, these saponins were identified or tentatively characterized. Guided by specific selection criteria, network pharmacology analysis was performed on 15 representative steroidal saponins, identifying the PI3K/Akt signaling pathway as a core underlying mechanism. Supported by the *in vitro* validation of *p*-PI3K and *p*-AKT expression levels, compounds T3-16 and T3-17 were confirmed to be the key bioactive components in ACGD responsible for antiplatelet aggregation effects via the PI3K/Akt pathway. Taken together, these findings provide solid scientific evidence for the effective substances in ACGD against platelet aggregation.

## Data Availability

The original contributions presented in the study are included in the article/Supplementary material, further inquiries can be directed to the corresponding authors.
