# Supplementary material for: An activity labelled molecular networking strategy-assisted discovery of antiplatelet aggregation-active components from Allium chinense G. Don
Source: Front Nutr. 2026 May 7;13:1815132. doi: 10.3389/fnut.2026.1815132 (PMC13190571; doi:10.3389/fnut.2026.1815132)
Supplement: Supplementary file 1 [file Supplementary_File_1.docx]

**An activity labelled molecular networking strategy assists the discovery of anti-platelet aggregation active components from *Allium chinense* G. Don**

Yan Xu ^1, †^, Beibei Zhang ^2, †^, Liangliang He ^3^, Qiwen Zhang ^2^, Yongjie Yang ^2^, Zhihong Yao ^3^, Jing Yang ^2,^ *, Qi Wang ^3,^ *, Zifei Qin ^2,^ *

^1^ Department of Hematology, the First Affiliated Hospital of Zhengzhou University, Zhengzhou 450052, China;

^2^ Department of Pharmacy, Henan Province Engineering Research Center of Application & Translation of Precision Clinical Pharmacy, the First Affiliated Hospital of Zhengzhou University, Zhengzhou, 450052, China;

^3^ State Key Laboratory of Bioactive Molecules and Druggability Assessment, International Cooperative Laboratory of Traditional Chinese Medicine Modernization and Innovative Drug Development of Ministry of Education (MOE) of China, Guangdong Basic Research Center of Excellence for Natural Bioactive Molecules and Discovery of Innovative Drugs, Guangdong Province Key Laboratory of Pharmacodynamic Constituents of TCM and New Drugs Research, College of Pharmacy, Jinan University, Guangzhou 510632, China;

^†^ These authors contributed equally to this work.

***Corresponding Authors:**

Dr. Zifei Qin, qzf1989@163.com;

Dr. Qi Wang, gatsby0604@163.com;

Dr. Jing Yang, jingyang_0101@163.com;

**Table list**

**Table S1** The purity of authentic standards used in this study.

**Figure caption**

**Figure S1** The platelet aggregation activity induced by AA (A), ADP (B) and collagen (C) in the absence of tested ACGD extracts (0 μM, control) at room temperature.

**Figure S2** The platelet aggregation activity induced by AA (A), ADP (B) and collagen (C) in the absence of tested ACGD extracts extracted by pure methanol at room temperature.

**Figure S3** The platelet aggregation activity induced by AA (A), ADP (B) and collagen (C) in the absence of tested ACGD extracts extracted by 30% methanol at room temperature.

**Figure S4** The platelet aggregation activity induced by AA (A), ADP (B) and collagen (C) in the absence of tested ACGD extracts extracted by 50% methanol at room temperature.

**Figure S5** The platelet aggregation activity induced by AA (A), ADP (B) and collagen (C) in the absence of tested ACGD extracts extracted by 70% methanol at room temperature.

**Figure S6** The platelet aggregation activity induced by AA (A), ADP (B) and collagen (C) in the absence of tested ACGD extracts extracted by pure water at room temperature.

**Figure S7** Comprehensive chemical overview of ACGD samples extracted by 70% methanol. The feature-based molecular networking of ACGD samples in positive (A) and negative (B) ion modes.

**Figure S8** The EIC image, MS and MS^2^ information of Macrostemonoside B. (A) the extracted ion chromatogram of *m/z* 1227.5975 at 10.73 min. (B) the MS chromatogram in positive ion mode. (C) the MS^2^ chromatogram in positive ion mode. The annotation suggested the proposed fragmentation mode.

**Figure S9** The MS and MS^2^ information for T1-2 saponin in ACGD sample. (A) extracted ion chromatogram of *m/z* 1065.5450 at 10.70 min. (B) comparison of the height of T1-2 in different ACGD extracts. (C) the MS chromatogram of T1-2 in negative ion mode. (D) the MS chromatogram of T1-2 in positive ion mode. (E) the MS^2^ chromatogram of T1-2 in positive ion mode. The annotation suggested the proposed fragmentation mode.

**Figure S10** The MS and MS^2^ information for T1-3 saponin in ACGD sample. (A) extracted ion chromatogram of *m/z* 903.4927 at 10.85 min. (B) comparison of the height of T1-3 in different ACGD extracts. (C) the MS chromatogram of T1-3 in negative ion mode. (D) the MS chromatogram of T1-3 in positive ion mode. (E) the MS^2^ chromatogram of T1-3 in positive ion mode. The annotation suggested the proposed fragmentation mode.

**Figure S11** The MS and MS^2^ information for T1-4 saponin in ACGD sample. (A) extracted ion chromatogram of *m/z* 1269.6080 at 10.94 min. (B) comparison of the height of T1-4 in different ACGD extracts. (C) the MS chromatogram of T1-4 in negative ion mode. (D) the MS chromatogram of T1-4 in positive ion mode. (E) the MS^2^ chromatogram of T1-4 in positive ion mode. The annotation suggested the proposed fragmentation mode.

**Figure S12** The MS and MS^2^ information for T1-5 saponin in ACGD sample. (A) extracted ion chromatogram of *m/z* 1107.5560 at 11.00 min. (B) comparison of the height of T1-5 in different ACGD extracts. (C) the MS chromatogram of T1-5 in negative ion mode. (D) the MS chromatogram of T1-5 in positive ion mode. (E) the MS^2^ chromatogram of T1-5 in positive ion mode. The annotation suggested the proposed fragmentation mode.

**Figure S13** The MS and MS^2^ information for T1-6 saponin in ACGD sample. (A) extracted ion chromatogram of *m/z* 1227.5990 at 11.44 min. (B) comparison of the height of T1-6 in different ACGD extracts. (C) the MS chromatogram of T1-6 in negative ion mode. (D) the MS chromatogram of T1-6 in positive ion mode. (E) the MS^2^ chromatogram of T1-6 in positive ion mode. The annotation suggested the proposed fragmentation mode.

**Figure S14** The MS and MS^2^ information for T1-7 saponin in ACGD sample. (A) extracted ion chromatogram of *m/z* 1065.5450 at 11.51 min. (B) comparison of the height of T1-7 in different ACGD extracts. (C) the MS chromatogram of T1-7 in negative ion mode. (D) the MS chromatogram of T1-7 in positive ion mode. (E) the MS^2^ chromatogram of T1-7 in positive ion mode. The annotation suggested the proposed fragmentation mode.

**Figure S15** The MS and MS^2^ information for T1-8 saponin in ACGD sample. (A) extracted ion chromatogram of *m/z* 1269.6100 at 11.73 min. (B) comparison of the height of T1-8 in different ACGD extracts. (C) the MS chromatogram of T1-8 in negative ion mode. (D) the MS chromatogram of T1-8 in positive ion mode. (E) the MS^2^ chromatogram of T1-8 in positive ion mode. The annotation suggested the proposed fragmentation mode.

**Figure S16** The MS and MS^2^ information for T1-9 saponin in ACGD sample. (A) extracted ion chromatogram of *m/z* 1065.5460 at 14.67 min. (B) comparison of the height of T1-9 in different ACGD extracts. (C) the MS chromatogram of T1-9 in negative ion mode. (D) the MS chromatogram of T1-9 in positive ion mode. (E) the MS^2^ chromatogram of T1-9 in positive ion mode. The annotation suggested the proposed fragmentation mode.

**Figure S17** The MS and MS^2^ information for T1-10 saponin in ACGD sample. (A) extracted ion chromatogram of *m/z* 925.4750 at 15.14 min. (B) comparison of the height of T1-10 in different ACGD extracts. (C) the MS chromatogram of T1-10 in negative ion mode. (D) the MS chromatogram of T1-10 in positive ion mode. (E) the MS^2^ chromatogram of T1-10 in positive ion mode. The annotation suggested the proposed fragmentation mode.

**Figure S18** The MS and MS^2^ information for T1-6 saponin in ACGD sample. (A) extracted ion chromatogram of *m/z* 1107.5560 at 15.52 min. (B) comparison of the height of T1-6 in different ACGD extracts. (C) the MS chromatogram of T1-6 in negative ion mode. (D) the MS chromatogram of T1-6 in positive ion mode. (E) the MS^2^ chromatogram of T1-6 in positive ion mode. The annotation suggested the proposed fragmentation mode.

**Figure S19** The MS and MS^2^ information of T2-2 saponin. (A) extracted ion chromatogram of *m/z* 1285.6040 at 10.50 min. (B) the height of T2-2 in different ACGD extracts. (C) the MS chromatogram of T2-2 in negative ion mode. (D) the MS chromatogram of T2-2 in positive ion mode. (E) the MS^2^ chromatogram of T2-2 in positive ion mode. The annotation explained the proposed fragmentation forms of T2-2.

**Figure S20** The MS and MS^2^ information of T2-3 saponin. (A) extracted ion chromatogram of *m/z* 1243.5940 at 11.05 min. (B) the height of T2-3 in different ACGD extracts. (C) the MS chromatogram of T2-3 in negative ion mode. (D) the MS chromatogram of T2-3 in positive ion mode. (E) the MS^2^ chromatogram of T2-3 in positive ion mode. The annotation explained the proposed fragmentation forms of T2-3.

**Figure S21** The MS and MS^2^ information of T2-4 saponin. (A) extracted ion chromatogram of *m/z* 919.4885 at 12.65 min. (B) the height of T2-4 in different ACGD extracts. (C) the MS chromatogram of T2-4 in negative ion mode. (D) the MS chromatogram of T2-4 in positive ion mode. (E) the MS^2^ chromatogram of T2-4 in positive ion mode. The annotation explained the proposed fragmentation forms of T2-4.

**Figure S22** The MS and MS^2^ information of T2-5 saponin. (A) extracted ion chromatogram of *m/z* 1081.5410 at 13.52 min. (B) the height of T2-5 in different ACGD extracts. (C) the MS chromatogram of T2-5 in negative ion mode. (D) the MS chromatogram of T2-5 in positive ion mode. (E) the MS^2^ chromatogram of T2-5 in positive ion mode. The annotation explained the proposed fragmentation forms of T2-5.

**Figure S23** The MS and MS^2^ information of T2-6 saponin. (A) extracted ion chromatogram of *m/z* 919.4885 at 13.82 min. (B) the height of T2-6 in different ACGD extracts. (C) the MS chromatogram of T2-6 in negative ion mode. (D) the MS chromatogram of T2-6 in positive ion mode. (E) the MS^2^ chromatogram of T2-6 in positive ion mode. The annotation explained the proposed fragmentation forms of T2-6.

**Figure S24** The MS and MS^2^ information of T2-7 saponin. (A) extracted ion chromatogram of *m/z* 433.3306 at 17.57 min. (B) the height of T2-7 in different ACGD extracts. (C) the MS chromatogram of T2-7 in positive ion mode. (D) the MS^2^ chromatogram of T2-7 in positive ion mode. The annotation explained the proposed fragmentation forms of T2-7.

**Figure S25** The detailed MS and MS^2^ information of T3-1 saponin. (A) extracted ion chromatogram of *m/z* 1017.4870 at 10.21 min. (B) the height analysis of T3-1 in different ACGD extracts. (C) the (-) ESI-MS chromatogram of T3-1. (D) the (+) ESI-MS chromatogram of T3-1. (E) the (+) ESI-MS^2^ chromatogram of T3-1. The proposed fragmentation mode was presented in the annotation.

**Figure S26** The detailed MS and MS^2^ information of T3-2 saponin. (A) extracted ion chromatogram of *m/z* 1181.5570 at 10.32 min. (B) the height analysis of T3-2 in different ACGD extracts. (C) the (-) ESI-MS chromatogram of T3-2. (D) the (+) ESI-MS chromatogram of T3-2. (E) the (+) ESI-MS^2^ chromatogram of T3-2. The proposed fragmentation mode was presented in the annotation.

**Figure S27** The detailed MS and MS^2^ information of T3-4 saponin. (A) extracted ion chromatogram of *m/z* 887.4610 at 10.42 min. (B) the height analysis of T3-4 in different ACGD extracts. (C) the (-) ESI-MS chromatogram of T3-4. (D) the (+) ESI-MS chromatogram of T3-4. (E) the (+) ESI-MS^2^ chromatogram of T3-4. The proposed fragmentation mode was presented in the annotation.

**Figure S28** The detailed MS and MS^2^ information of T3-5 saponin. (A) extracted ion chromatogram of *m/z* 885.4426 at 10.48 min. (B) the height analysis of T3-5 in different ACGD extracts. (C) the (-) ESI-MS chromatogram of T3-5. (D) the (+) ESI-MS chromatogram of T3-5. (E) the (+) ESI-MS^2^ chromatogram of T3-5. The proposed fragmentation mode was presented in the annotation.

**Figure S29** The detailed MS and MS^2^ information of T3-6 saponin. (A) extracted ion chromatogram of *m/z* 1061.5140 at 10.68 min. (B) the height analysis of T3-6 in different ACGD extracts. (C) the (-) ESI-MS chromatogram of T3-6. (D) the (+) ESI-MS chromatogram of T3-6. (E) the (+) ESI-MS^2^ chromatogram of T3-6. The proposed fragmentation mode was presented in the annotation.

**Figure S30** The detailed MS and MS^2^ information of T3-7 saponin. (A) extracted ion chromatogram of *m/z* 929.4725 at 10.72 min. (B) the height analysis of T3-7 in different ACGD extracts. (C) the (-) ESI-MS chromatogram of T3-7. (D) the (+) ESI-MS chromatogram of T3-7. (E) the (+) ESI-MS^2^ chromatogram of T3-7. The proposed fragmentation mode was presented in the annotation.

**Figure S31** The detailed MS and MS^2^ information of T3-8 saponin. (A) extracted ion chromatogram of *m/z* 1049.5130 at 10.94 min. (B) the height analysis of T3-8 in different ACGD extracts. (C) the (-) ESI-MS chromatogram of T3-8. (D) the (+) ESI-MS chromatogram of T3-8. (E) the (+) ESI-MS^2^ chromatogram of T3-8. The proposed fragmentation mode was presented in the annotation.

**Figure S32** The detailed MS and MS^2^ information of T3-9 saponin. (A) extracted ion chromatogram of *m/z* 1019.5030 at 11.00 min. (B) the height analysis of T3-9 in different ACGD extracts. (C) the (-) ESI-MS chromatogram of T3-9. (D) the (+) ESI-MS chromatogram of T3-9. (E) the (+) ESI-MS^2^ chromatogram of T3-9. The proposed fragmentation mode was presented in the annotation.

**Figure S33** The detailed MS and MS^2^ information of T3-10 saponin. (A) extracted ion chromatogram of *m/z* 887.4614 at 11.00 min. (B) the height analysis of T3-10 in different ACGD extracts. (C) the (-) ESI-MS chromatogram of T3-10. (D) the (+) ESI-MS chromatogram of T3-10. (E) the (+) ESI-MS^2^ chromatogram of T3-10. The proposed fragmentation mode was presented in the annotation.

**Figure S34** The detailed MS and MS^2^ information of T3-11 saponin. (A) extracted ion chromatogram of *m/z* 929.4720 at 11.35 min. (B) the height analysis of T3-11 in different ACGD extracts. (C) the (-) ESI-MS chromatogram of T3-11. (D) the (+) ESI-MS chromatogram of T3-11. (E) the (+) ESI-MS^2^ chromatogram of T3-11. The proposed fragmentation mode was presented in the annotation.

**Figure S35** The detailed MS and MS^2^ information of T3-12 saponin. (A) extracted ion chromatogram of *m/z* 1017.4920 at 13.23 min. (B) the height analysis of T3-12 in different ACGD extracts. (C) the (-) ESI-MS chromatogram of T3-12. (D) the (+) ESI-MS chromatogram of T3-12. (E) the (+) ESI-MS^2^ chromatogram of T3-12. The proposed fragmentation mode was presented in the annotation.

**Figure S36** The detailed MS and MS^2^ information of T3-13 saponin. (A) extracted ion chromatogram of *m/z* 887.4620 at 13.30 min. (B) the height analysis of T3-13 in different ACGD extracts. (C) the (-) ESI-MS chromatogram of T3-13. (D) the (+) ESI-MS chromatogram of T3-13. (E) the (+) ESI-MS^2^ chromatogram of T3-13. The proposed fragmentation mode was presented in the annotation.

**Figure S37** The detailed MS and MS^2^ information of T3-14 saponin. (A) extracted ion chromatogram of *m/z* 857.4506 at 13.48 min. (B) the height analysis of T3-14 in different ACGD extracts. (C) the (-) ESI-MS chromatogram of T3-14. (D) the (+) ESI-MS chromatogram of T3-14. (E) the (+) ESI-MS^2^ chromatogram of T3-14. The proposed fragmentation mode was presented in the annotation.

**Figure S38** The detailed MS and MS^2^ information of T3-15 saponin. (A) extracted ion chromatogram of *m/z* 747.3909 at 13.67 min. (B) the height analysis of T3-15 in different ACGD extracts. (C) the (-) ESI-MS chromatogram of T3-15. (D) the (+) ESI-MS chromatogram of T3-15. (E) the (+) ESI-MS^2^ chromatogram of T3-15. The proposed fragmentation mode was presented in the annotation.

**Figure S39** The detailed MS and MS^2^ information of T3-16 saponin. (A) extracted ion chromatogram of *m/z* 723.3970 at 14.52 min. (B) the height analysis of T3-16 in different ACGD extracts. (C) the (-) ESI-MS chromatogram of T3-16. (D) the (+) ESI-MS chromatogram of T3-16. (E) the (+) ESI-MS^2^ chromatogram of T3-16. The proposed fragmentation mode was presented in the annotation.

**Figure S40** The detailed MS and MS^2^ information of T3-17 saponin. (A) extracted ion chromatogram of *m/z* 637.3604 at 15.00 min. (B) the height analysis of T3-17 in different ACGD extracts. (C) the (-) ESI-MS chromatogram of T3-17. (D) the (+) ESI-MS chromatogram of T3-17. (E) the (+) ESI-MS^2^ chromatogram of T3-17. The proposed fragmentation mode was presented in the annotation.

**Figure S41** The detailed MS and MS^2^ information of T3-18 saponin. (A) extracted ion chromatogram of *m/z* 431.3149 at 19.00 min. (B) the height analysis of T3-18 in different ACGD extracts. (C) the (+) ESI-MS chromatogram of T3-18. (D) the (+) ESI-MS^2^ chromatogram of T3-18. The proposed fragmentation mode was presented in the annotation.

**Table S1** The purity of authentic standards used in this study.

| **Compound name** | **No.** | **Purity** |
| --- | --- | --- |
| Macrostemonoside B | T1-1 | 97.38% |
| (25R)-26-O-β-D-glucopyranosyl-5α-furost-3β,26-didyroxy-3-O-{O-β-D-glucopyranosyl-(1→4)-β-D-galactopyranoside} | T1-3 | 95.38% |
| Macrostemonoside E | T1-6 | 89.57% |
| Macrostemonoside A | T1-9 | 98.06% |
| (25S)-5α-spirostane-3β-ol-3-O-{O-β-D-glucopyranosyl-(1→2)-O-β-D-glucopyranosyl-(1→4)-β-D-galactopyranoside} | T1-10 | 99.20% |
| Macrostemonoside D | T1-11 | 82.98% |
| Macrostemonoside R | T2-1 | 89.96% |
| (25R)-5α-spirostane-2α,3β-diol-3-O-{O-β-D-glucopyranosyl-(1→2)-O-β-D-glucopyranosyl-(1→4)-β-D-galactopyranoside} | T2-6 | 99.63% |
| Gitogenin | T2-7 | 98.65% |
| Chinenoside I | T3-3 | 97.87% |
| 26-O-β-D-glucopyranosyl-3β,22,26-tridyroxy-25(R)-5α-furostan-6-one-3-O-α-L-arabinopyranosyl-(1→6)-β-D-glucopyranoside | T3-4 | 70.99% |
| Chinenoside II | T3-9 | 95.64% |
| (25R)-3β-hydroxy-5α-spirostan-6-one-3-O-{O-β-D-glucopyranosyl-(1→3)-O-β-D-xylopyranosyl-(1→4)-O-[α-L-arabinopyranosyl-(1→6)]}-β-D-glucopyranoside | T3-12 | 72.35% |
| Laxogenin-3-O-{β-D-xylopyranosyl-(1→4)-O-[α-L-arabinopyranosyl-(1→6)]-β-D-glucopyranoside} | T3-14 | 98.15% |
| Laxogenin-3-O-{β-D-xylopyranosyl-(1→4)-β-D-glucopyranoside} | T3-15 | 99.09% |
| Laxogenin-3-O-{α-L-arabinopyranosyl-(1→6)-β-D-glucopyranoside} | T3-16 | 98.90% |
| Laxogenin-3-O-β-D-glucopyranoside | T3-17 | 98.67% |
| Laxogenin | T3-18 | 99.69% |


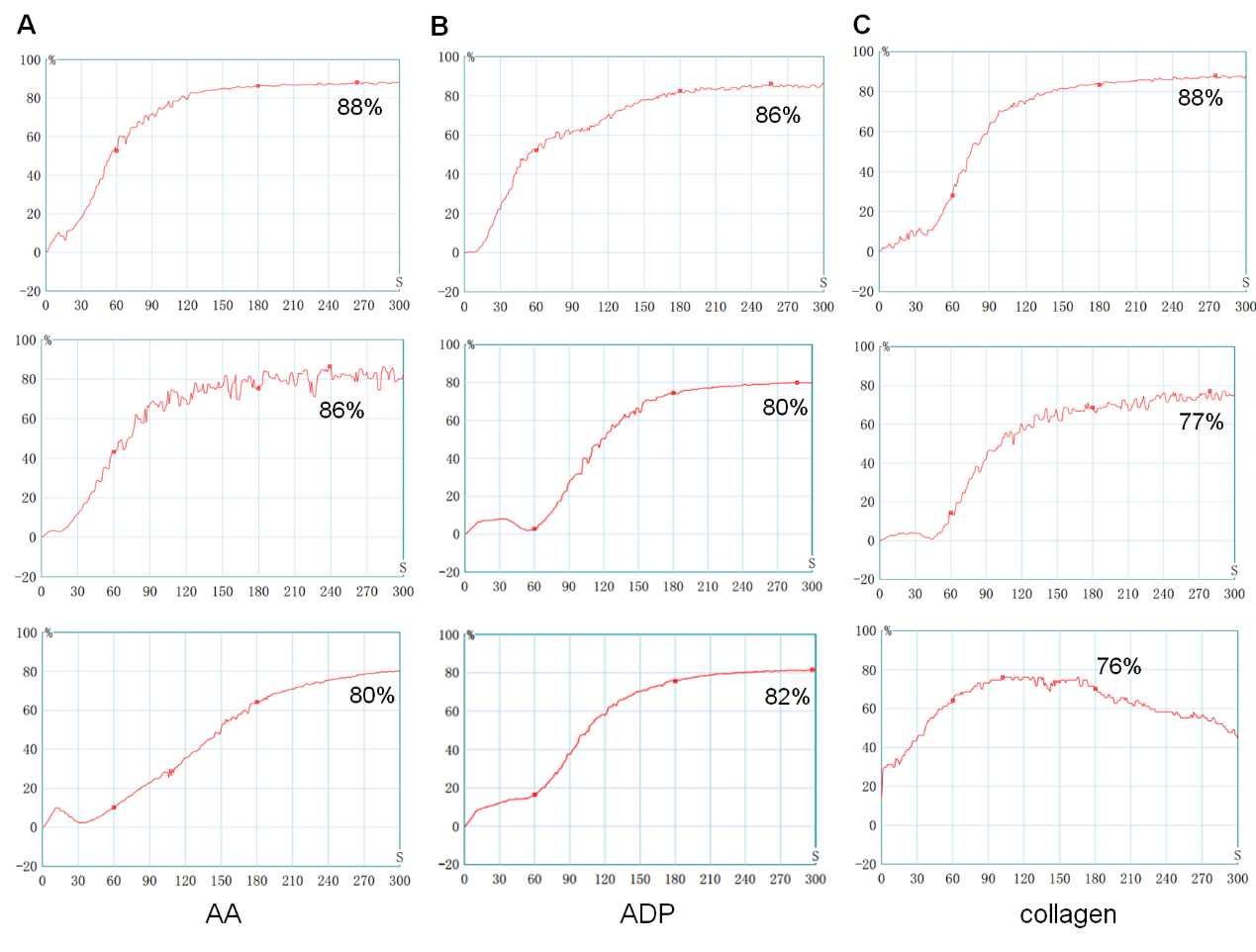


**Figure S1**


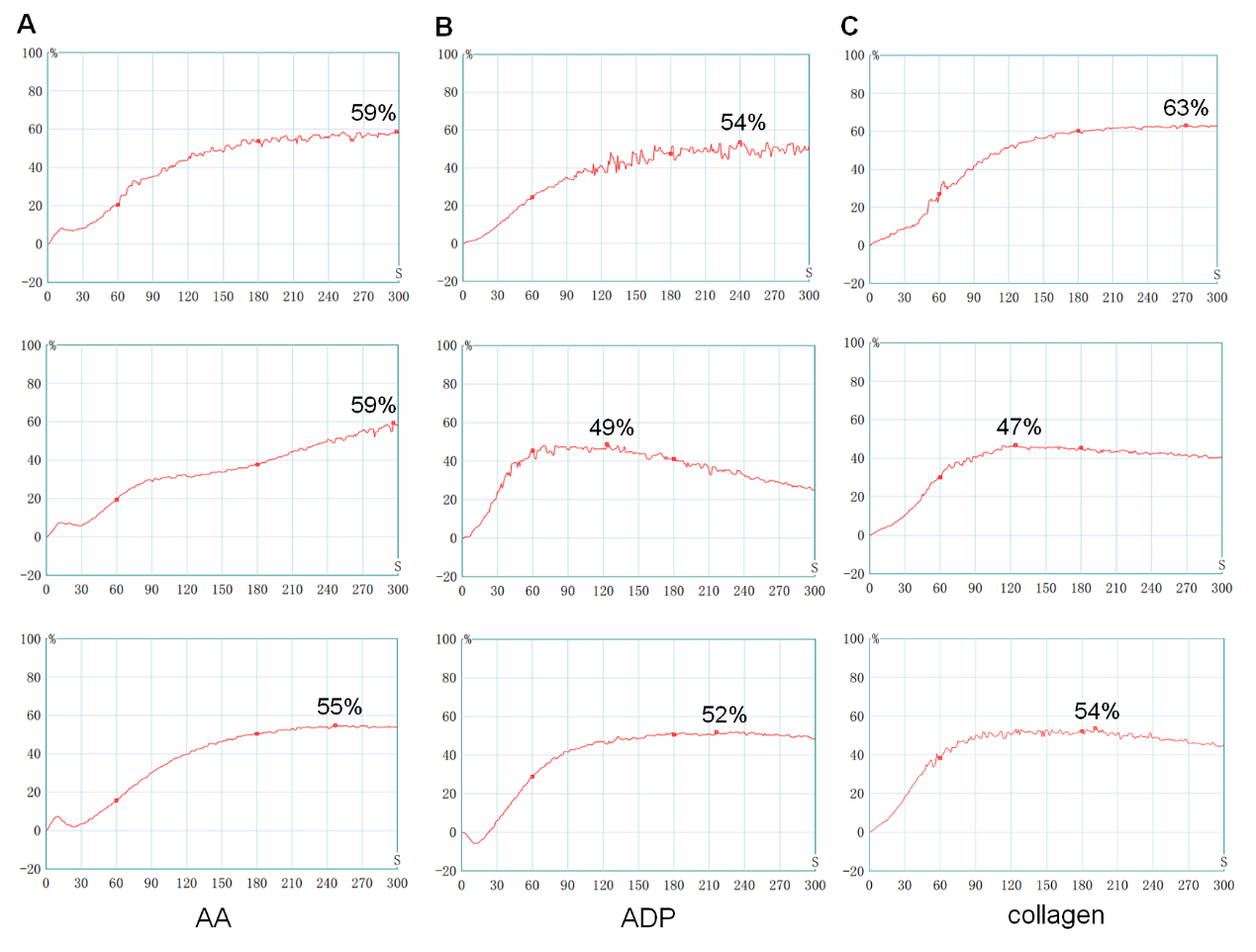


**Figure S2**


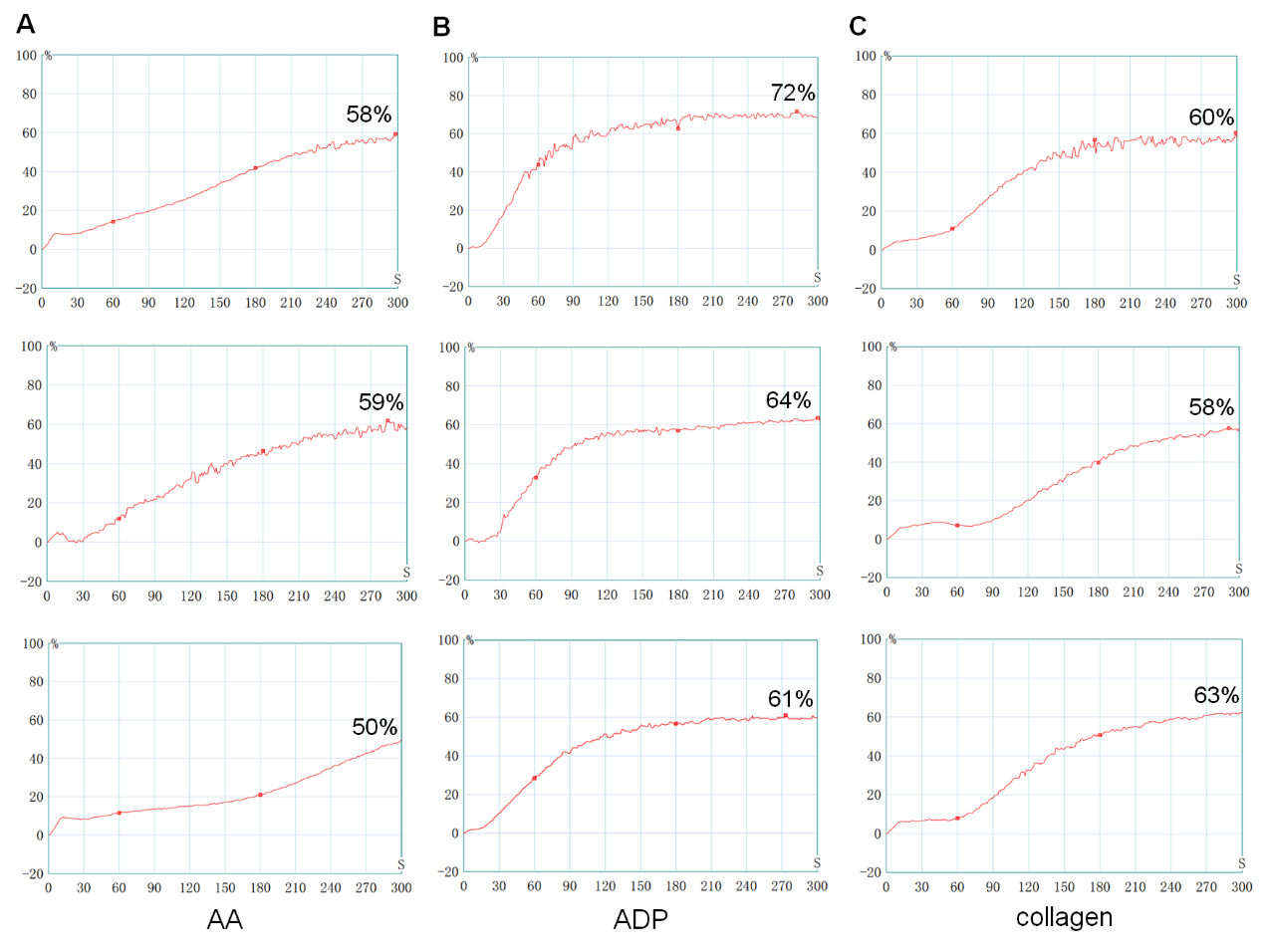


**Figure S3**


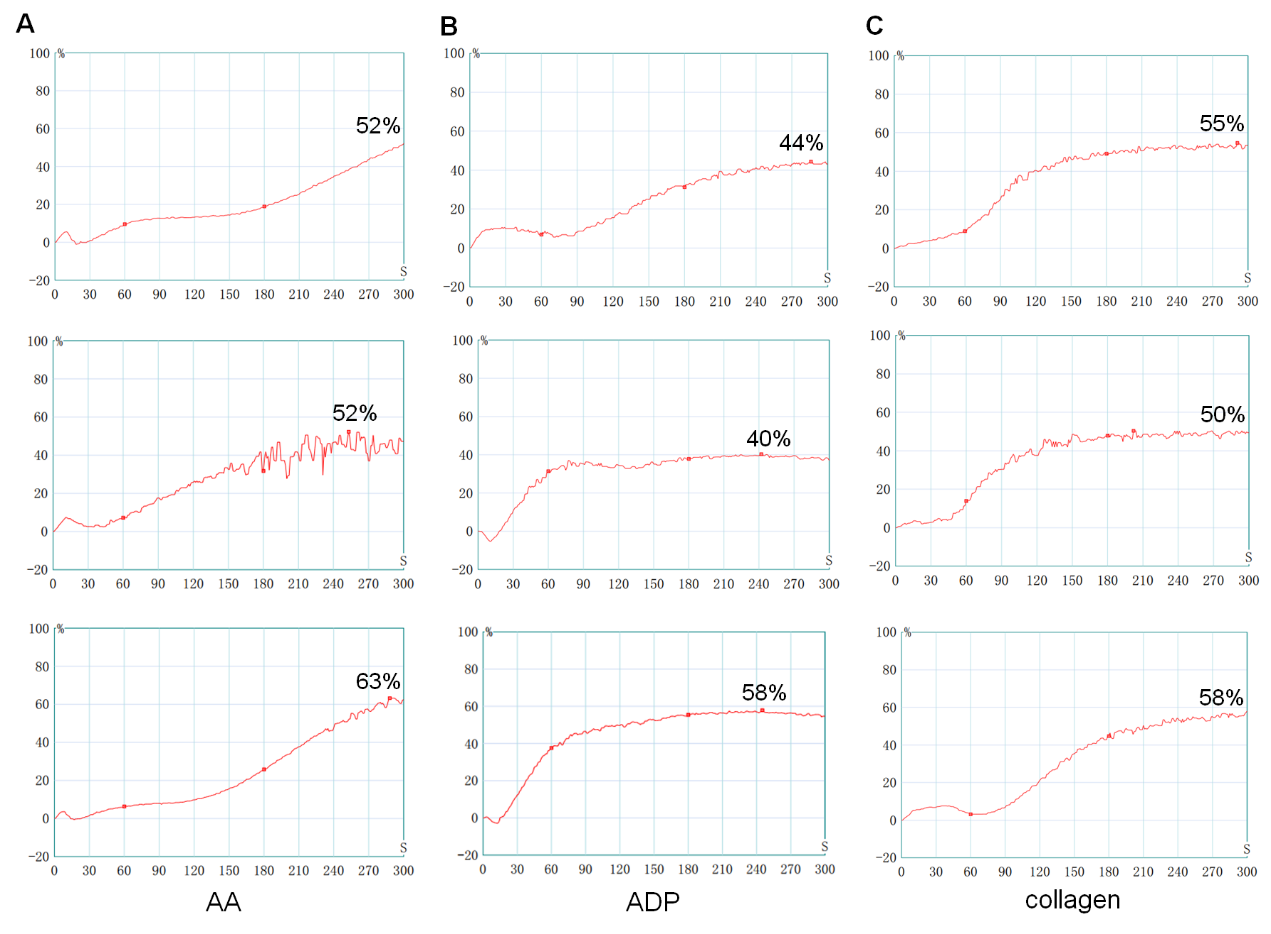


**Figure S4**


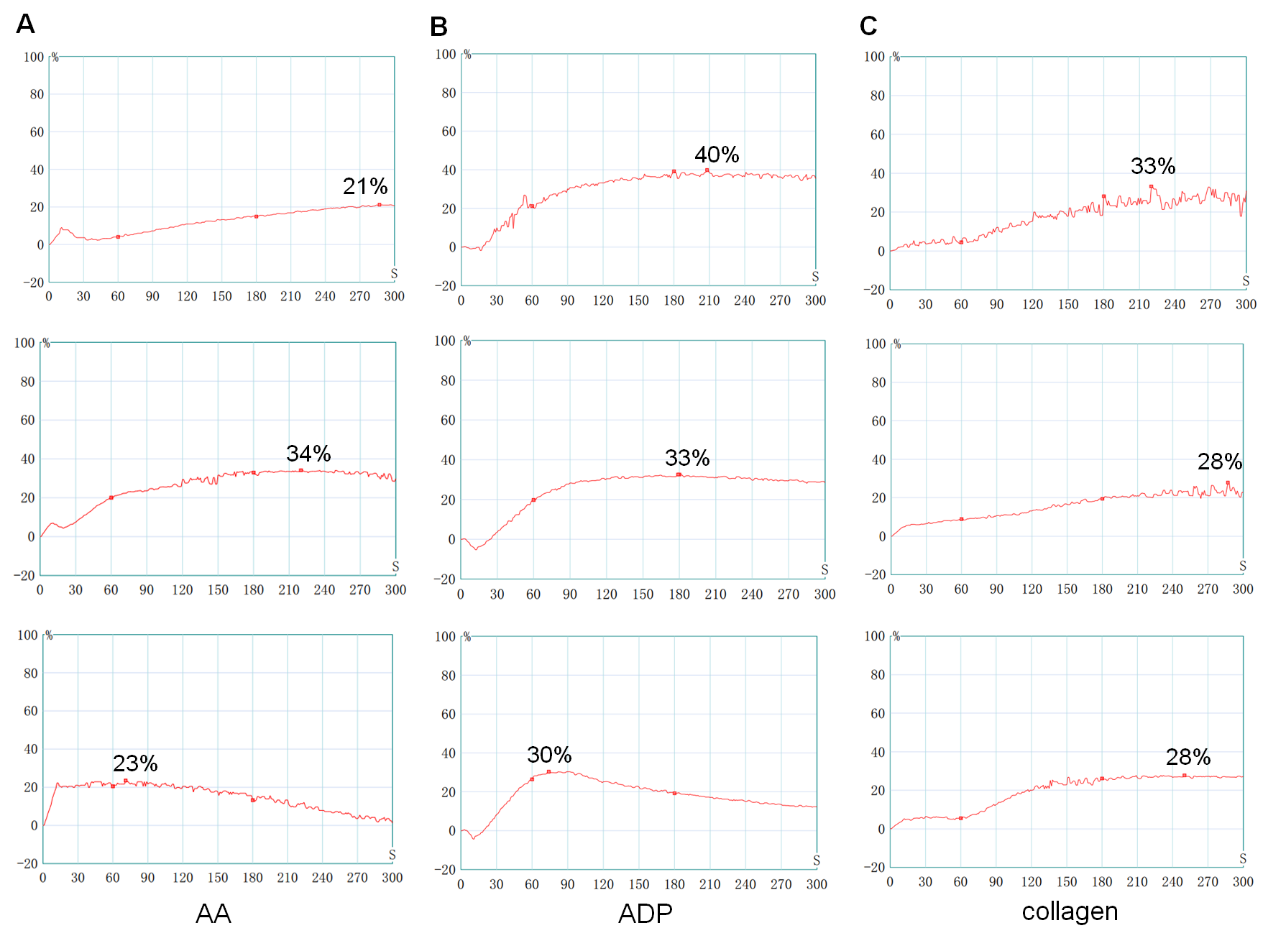


**Figure S5**


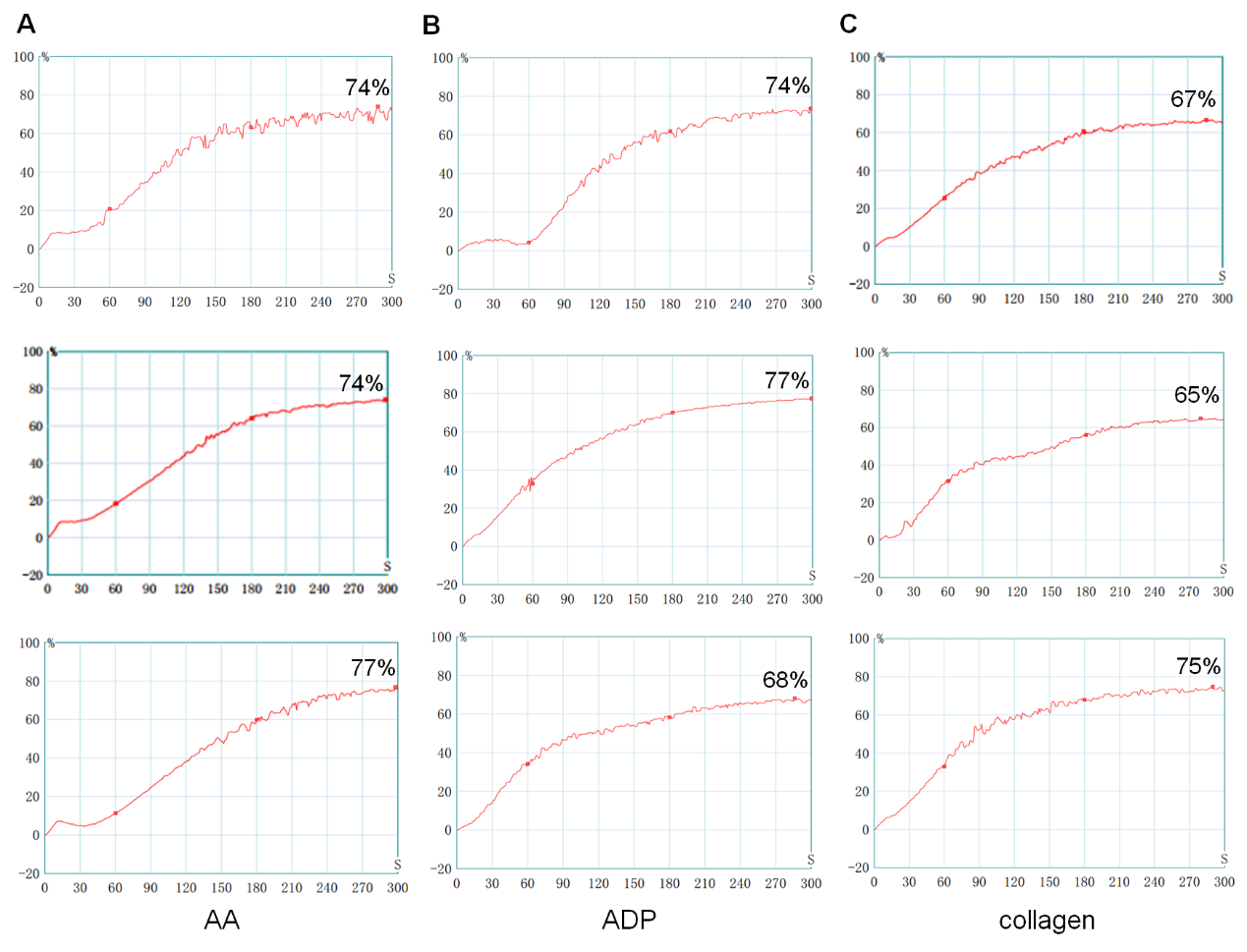


**Figure S6**


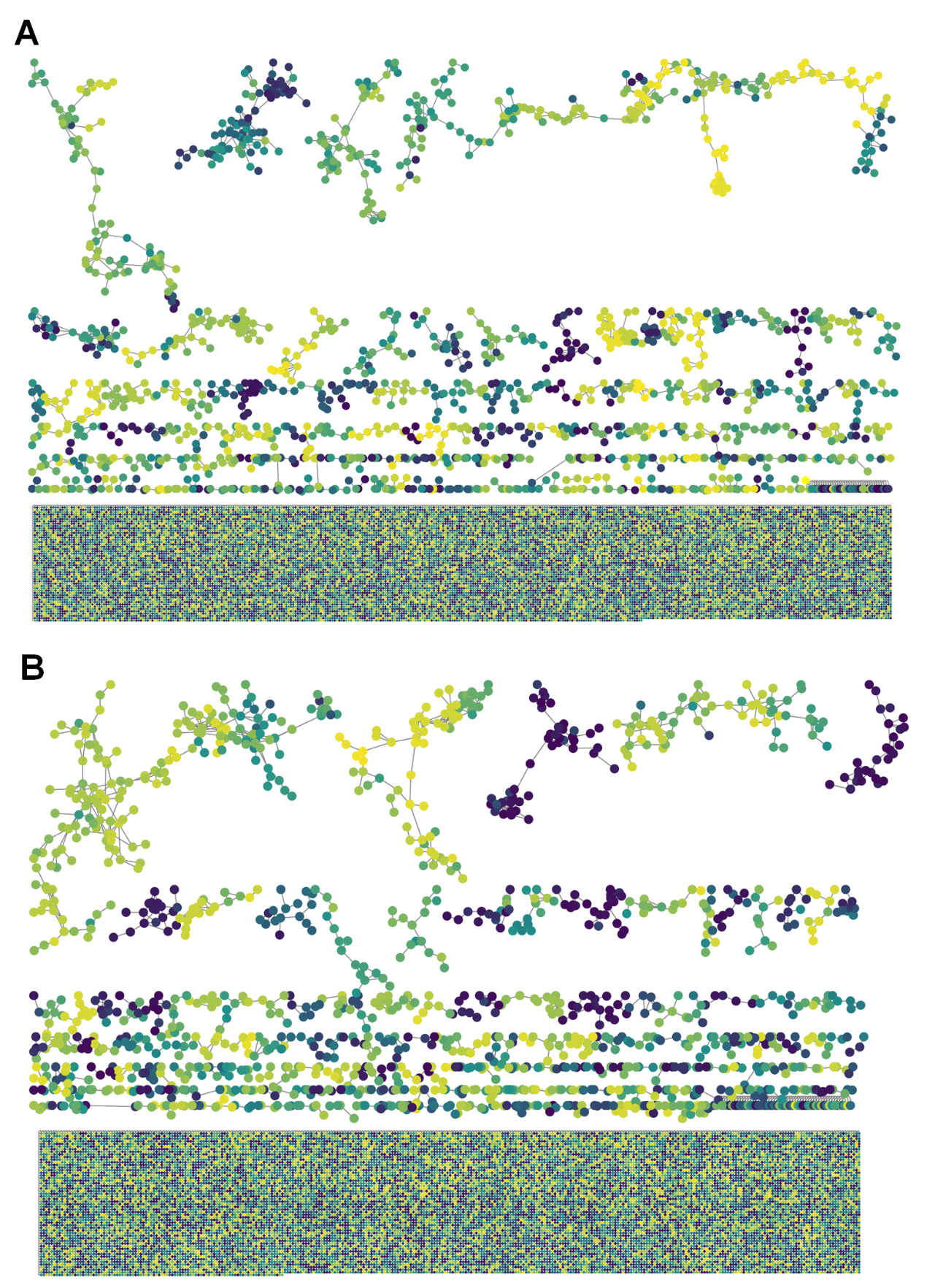


**Figure S7**


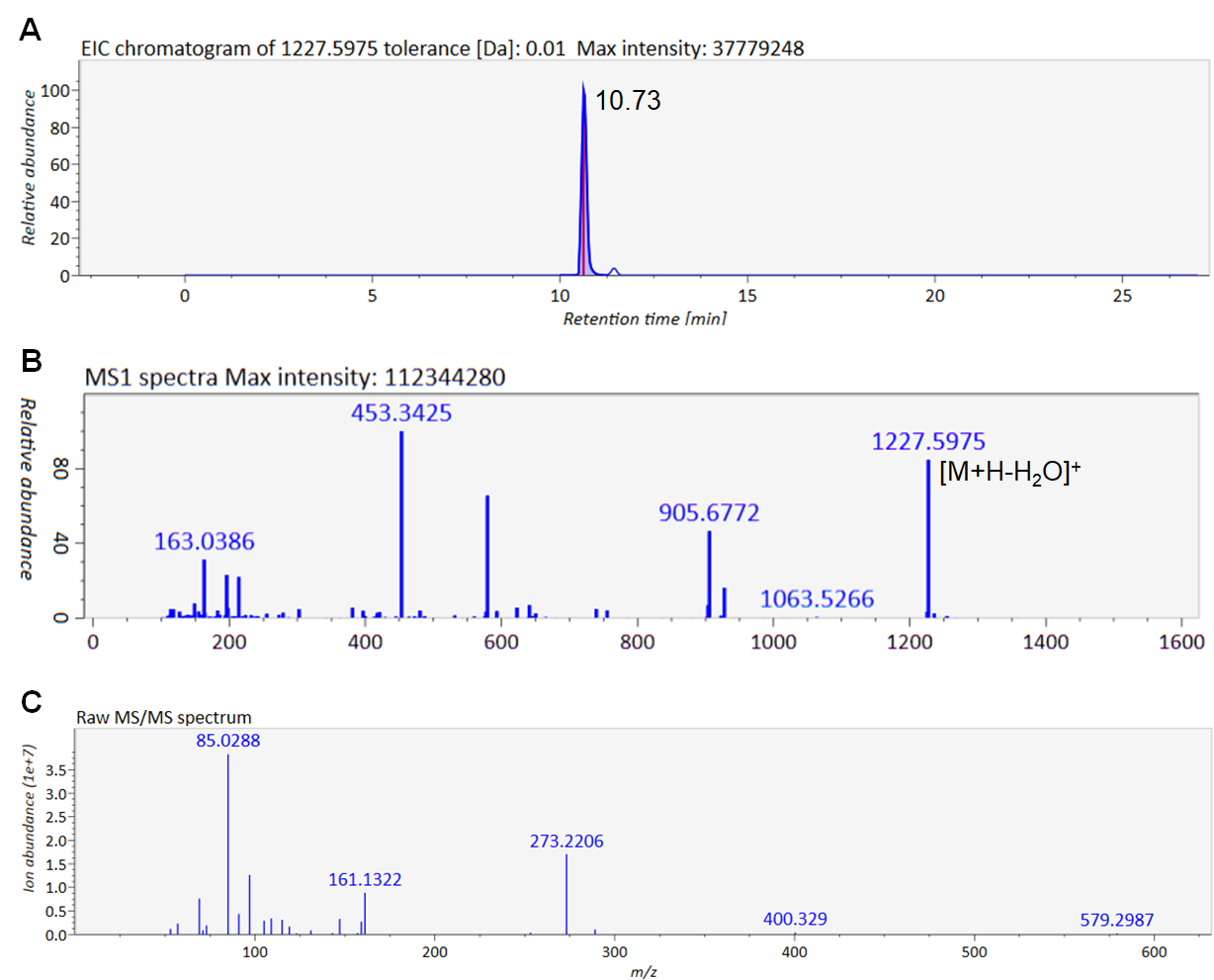


**Figure S8**


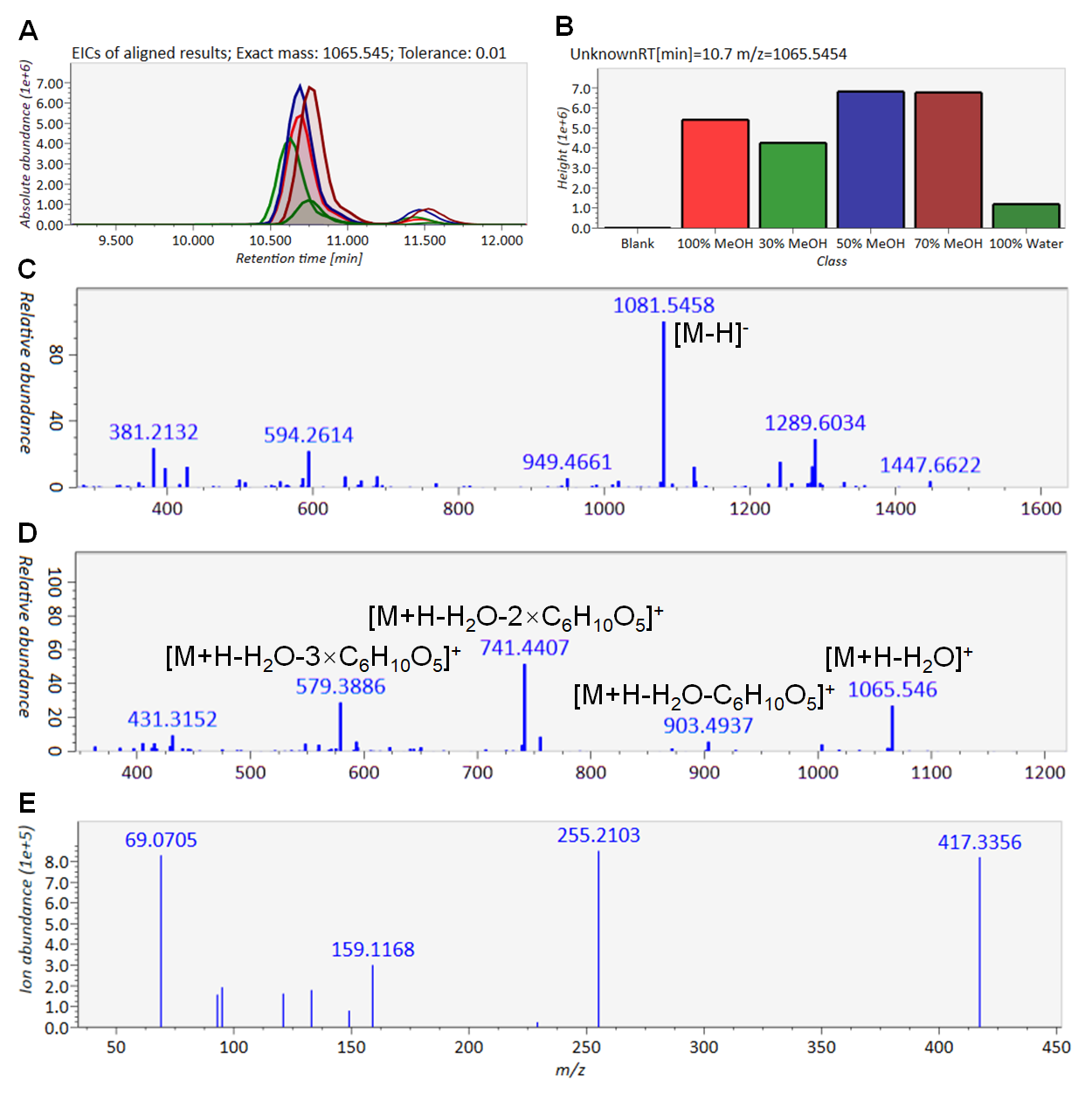


**Figure S9**


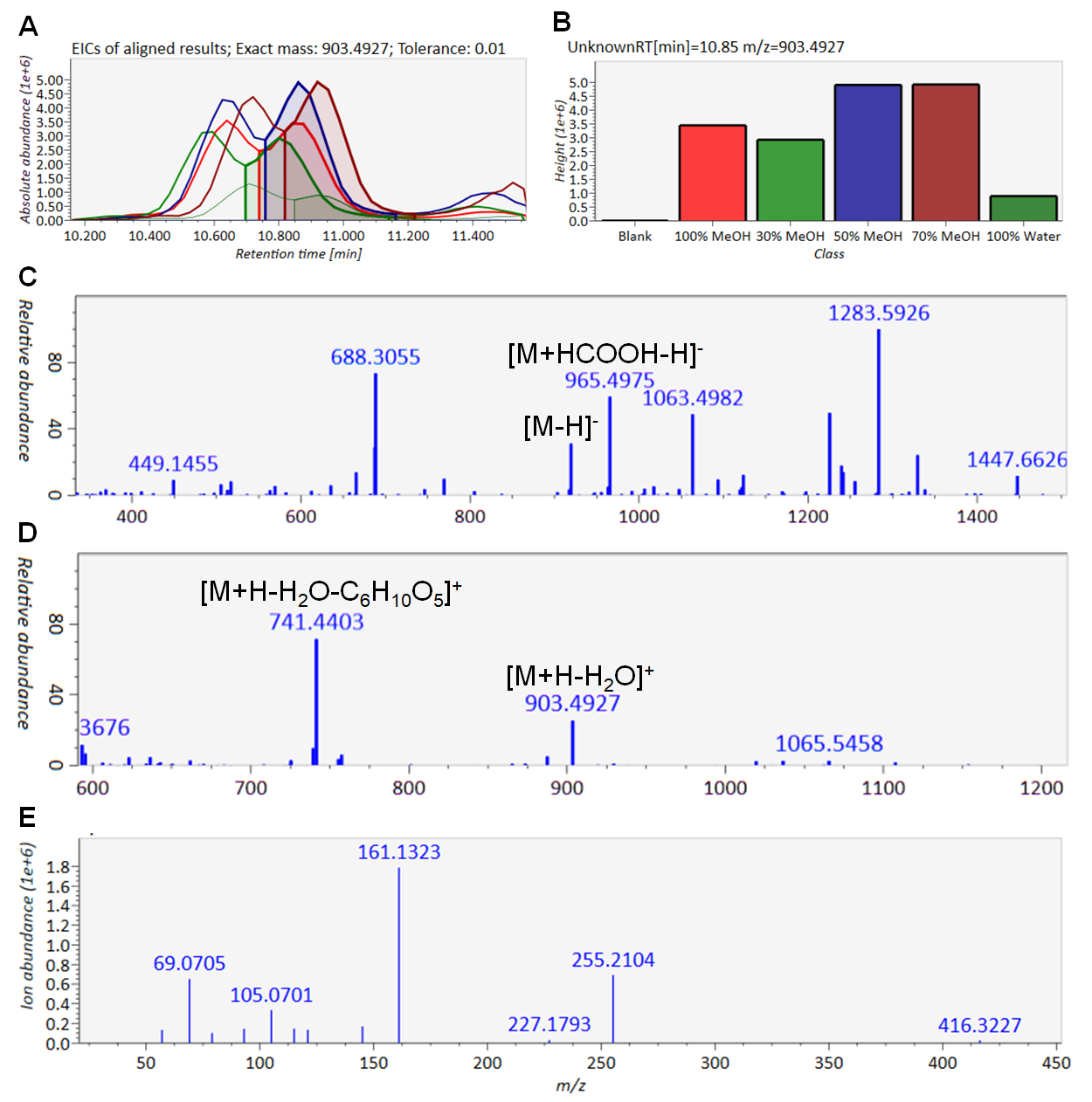


**Figure S10**


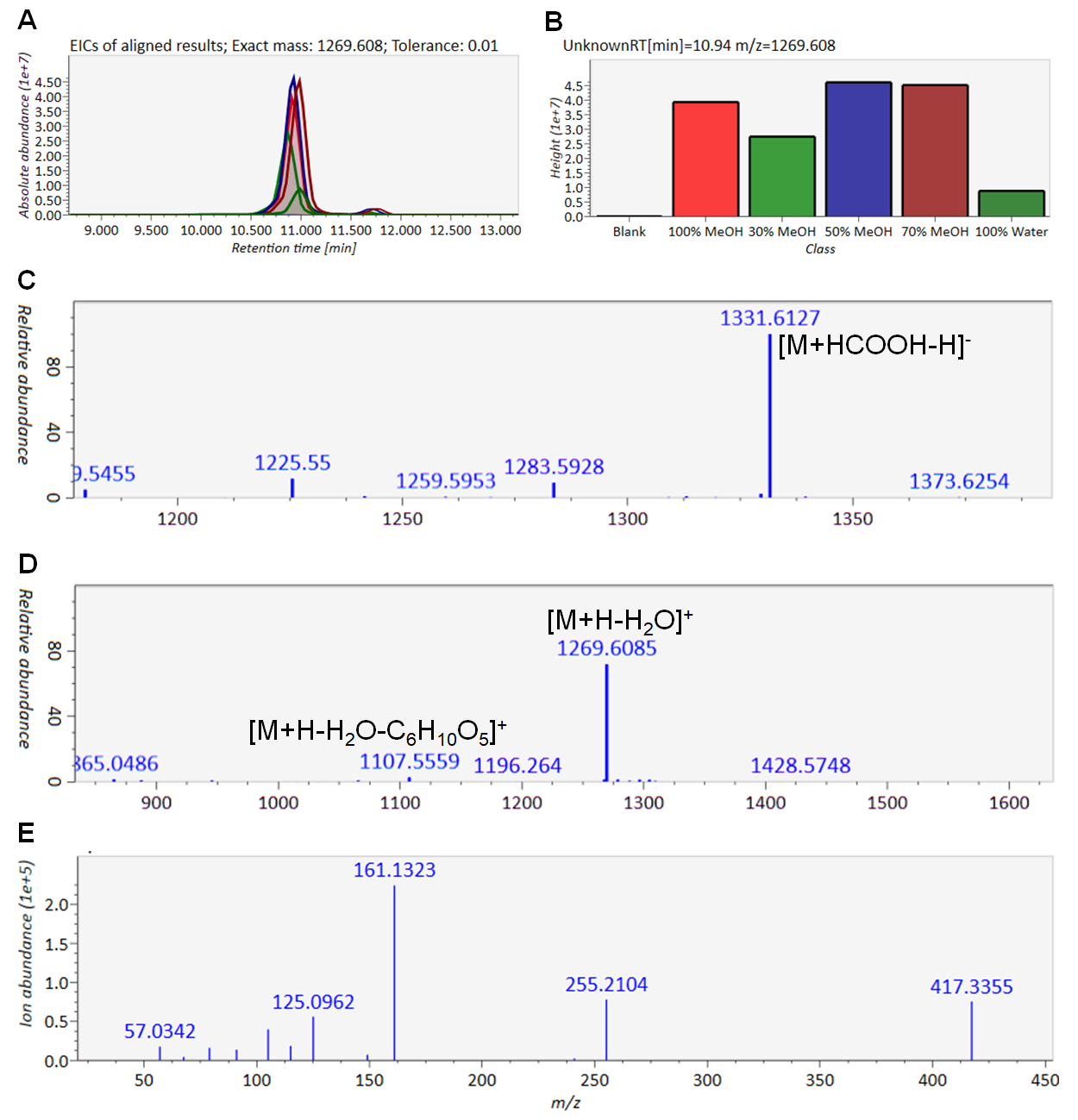


**Figure S11**


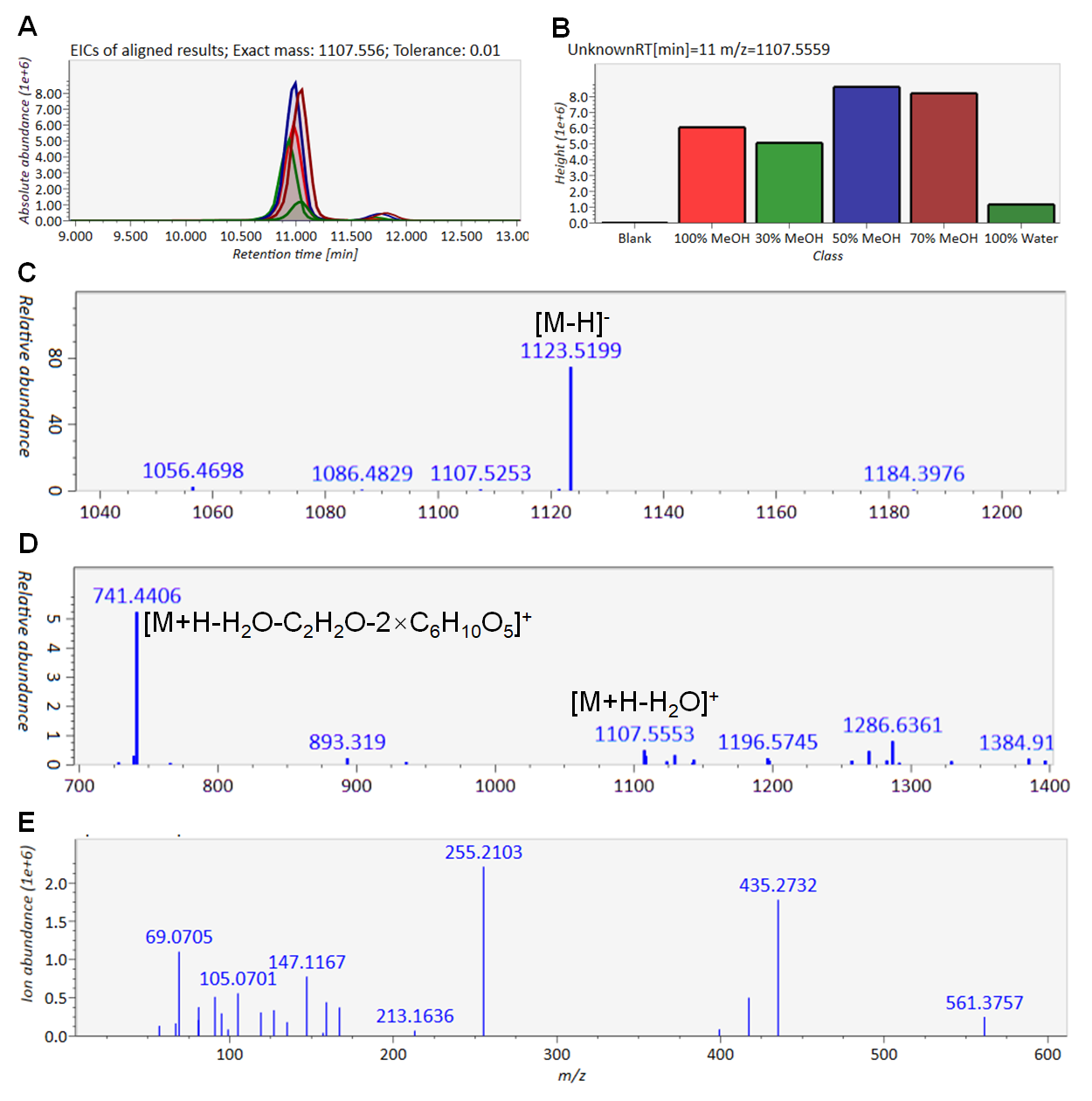


**Figure S12**


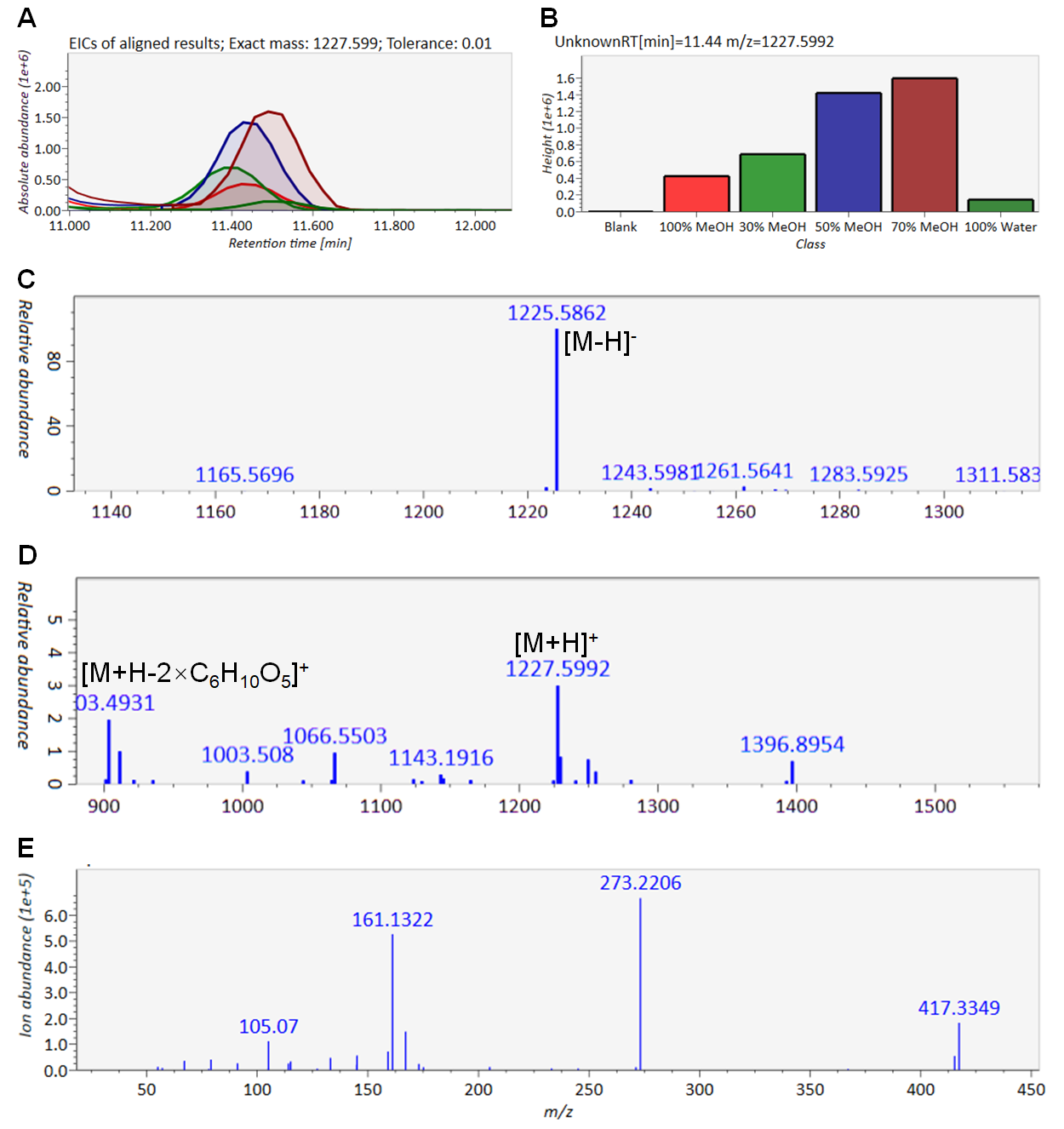


**Figure S13**


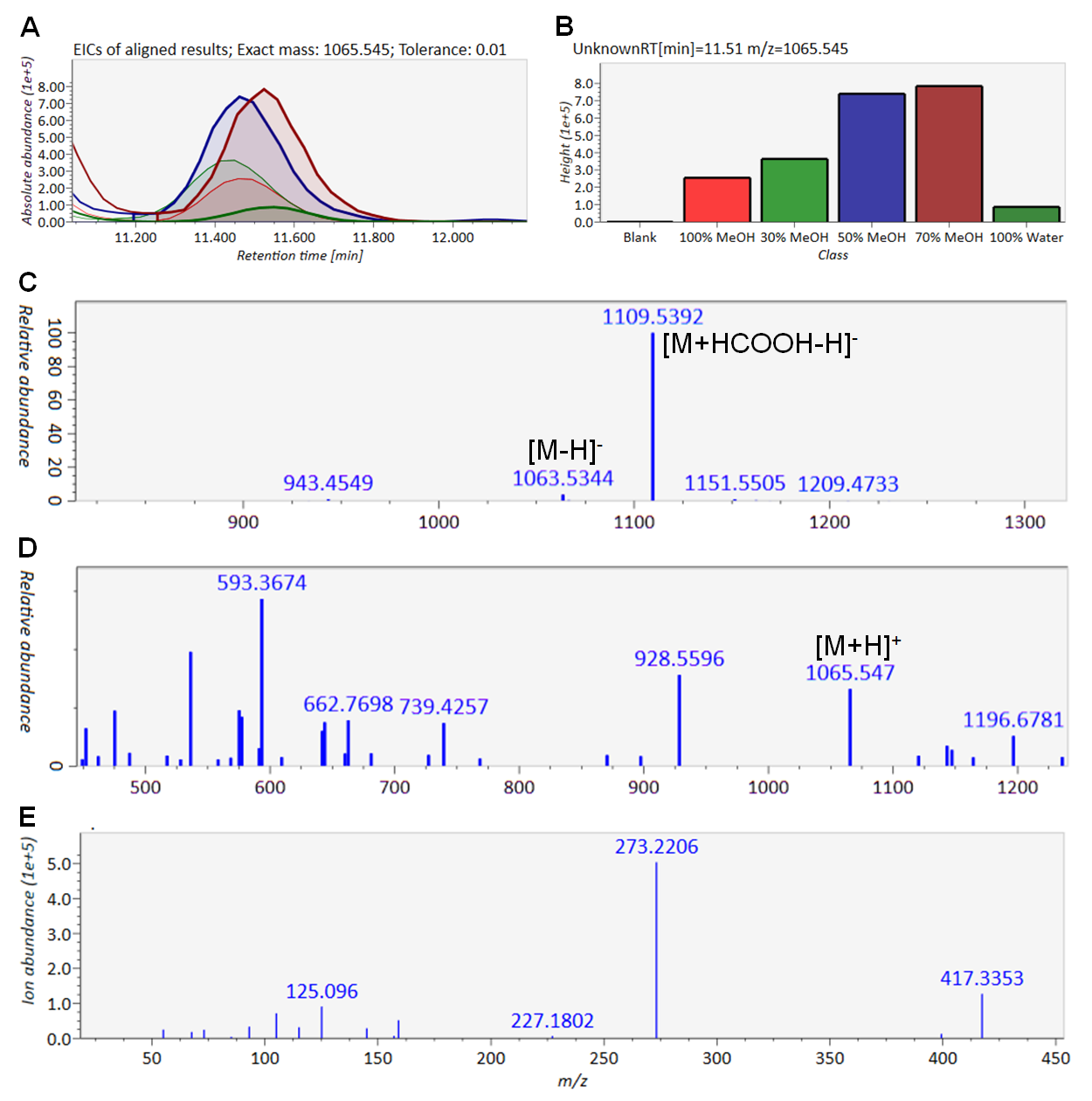


**Figure S14**


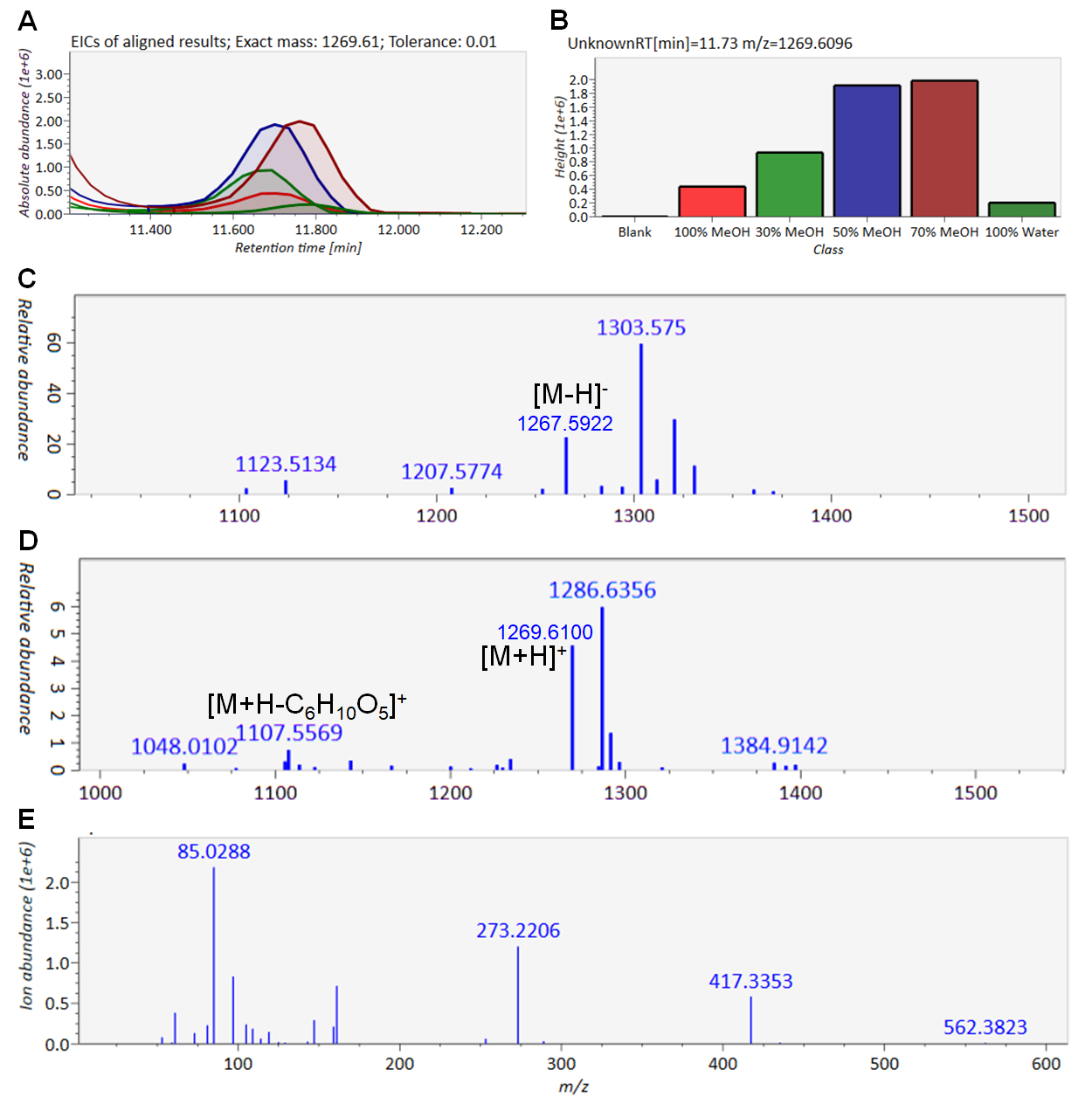


**Figure S15**


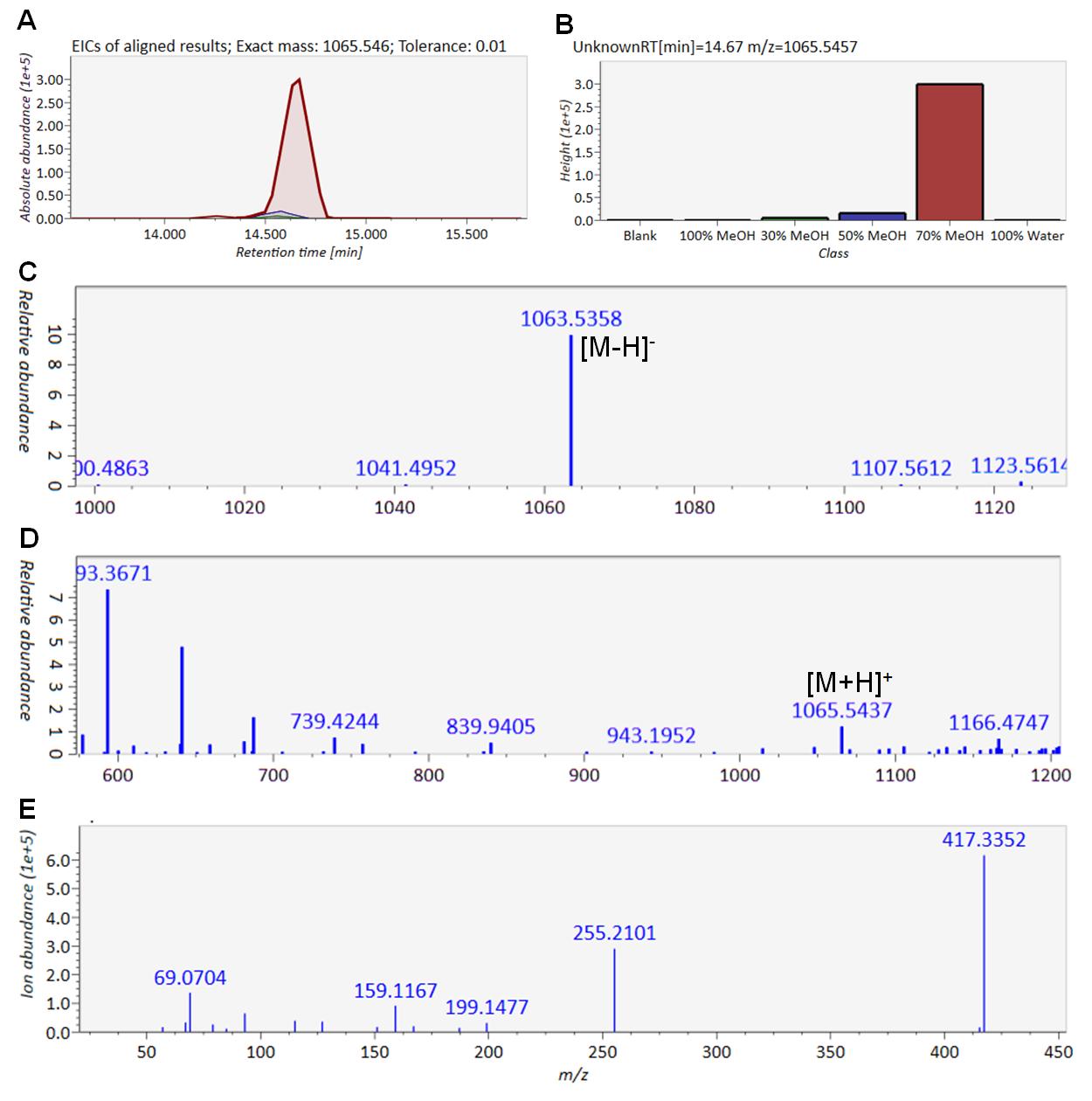


**Figure S16**


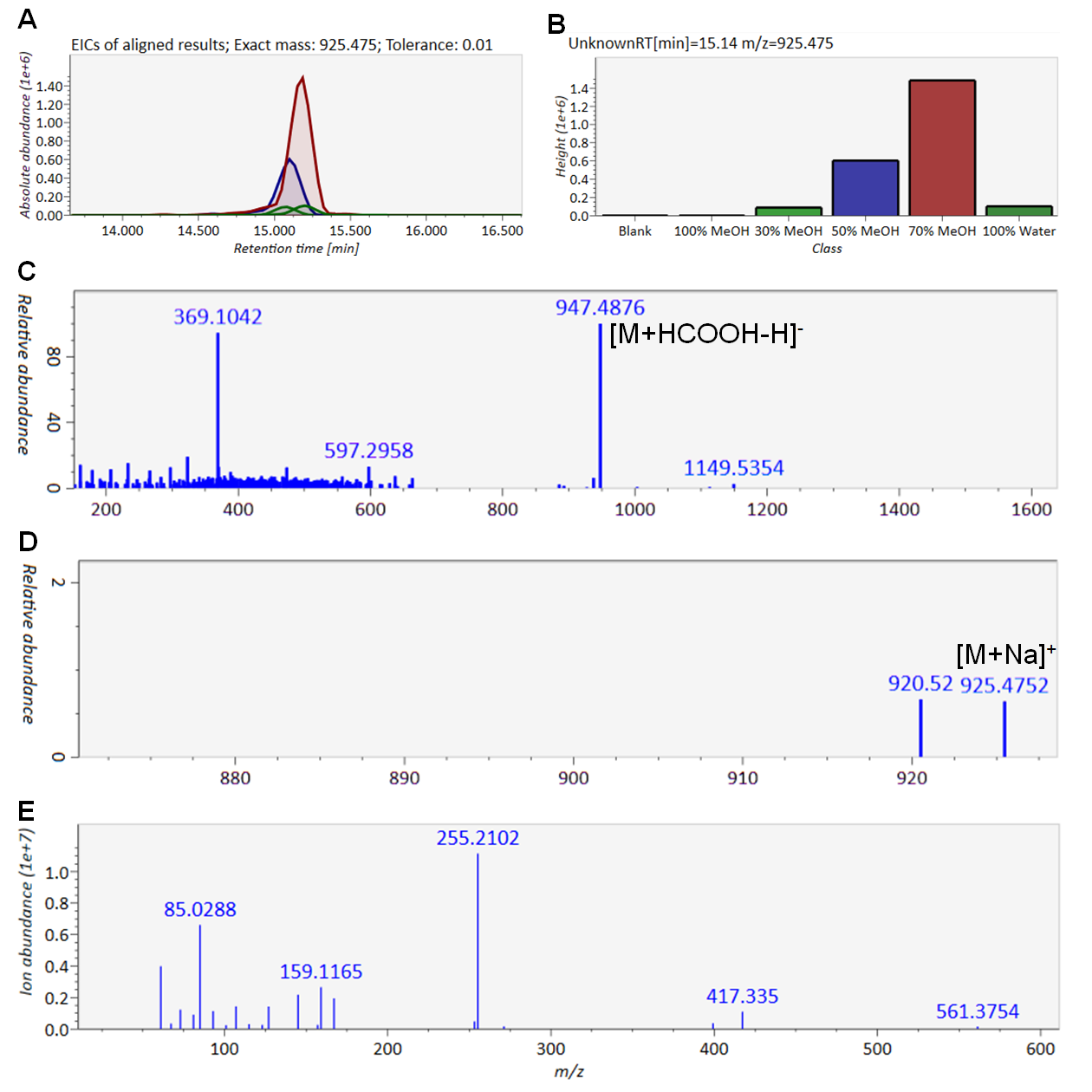


**Figure S17**


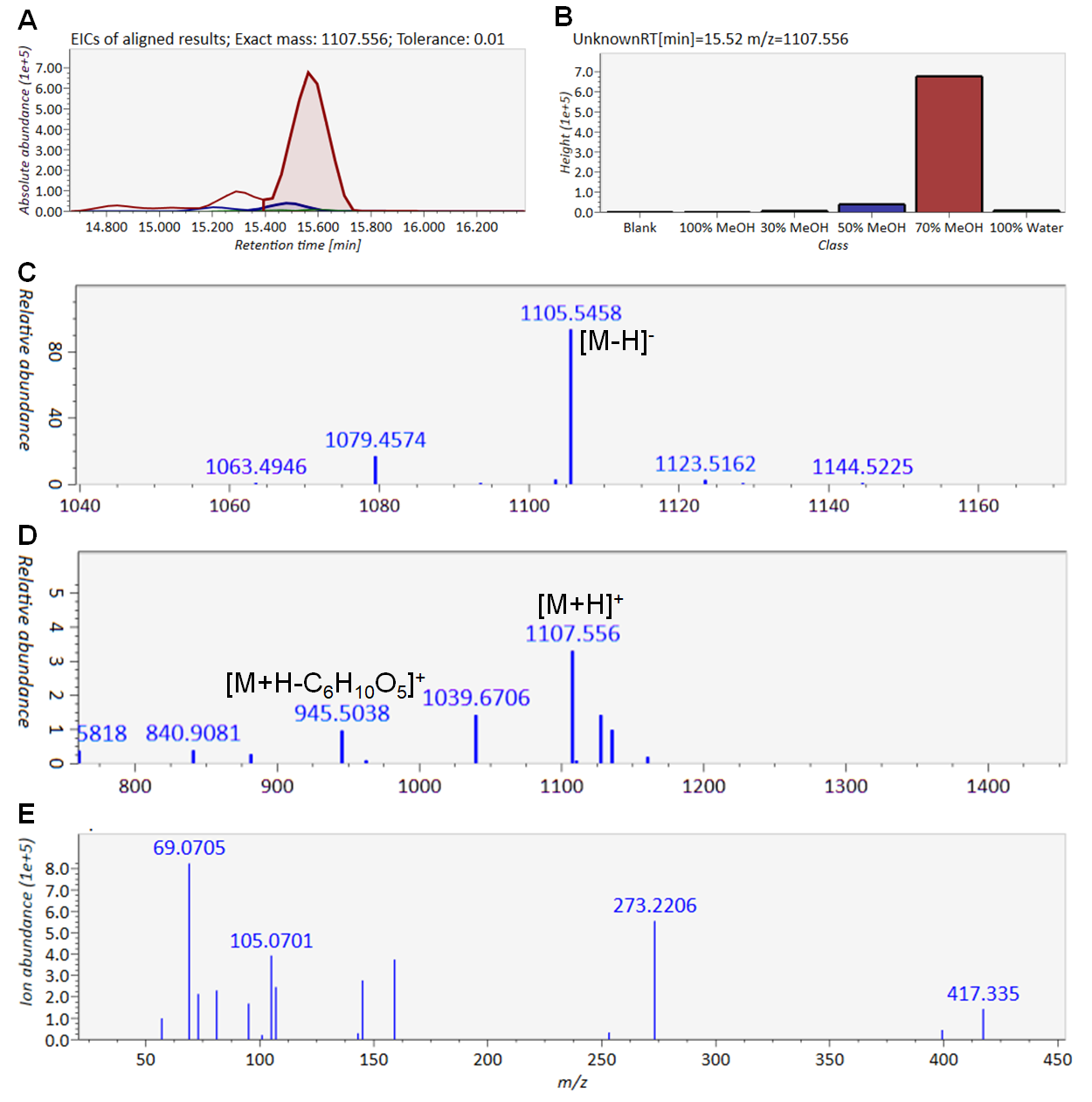


**Figure S18**


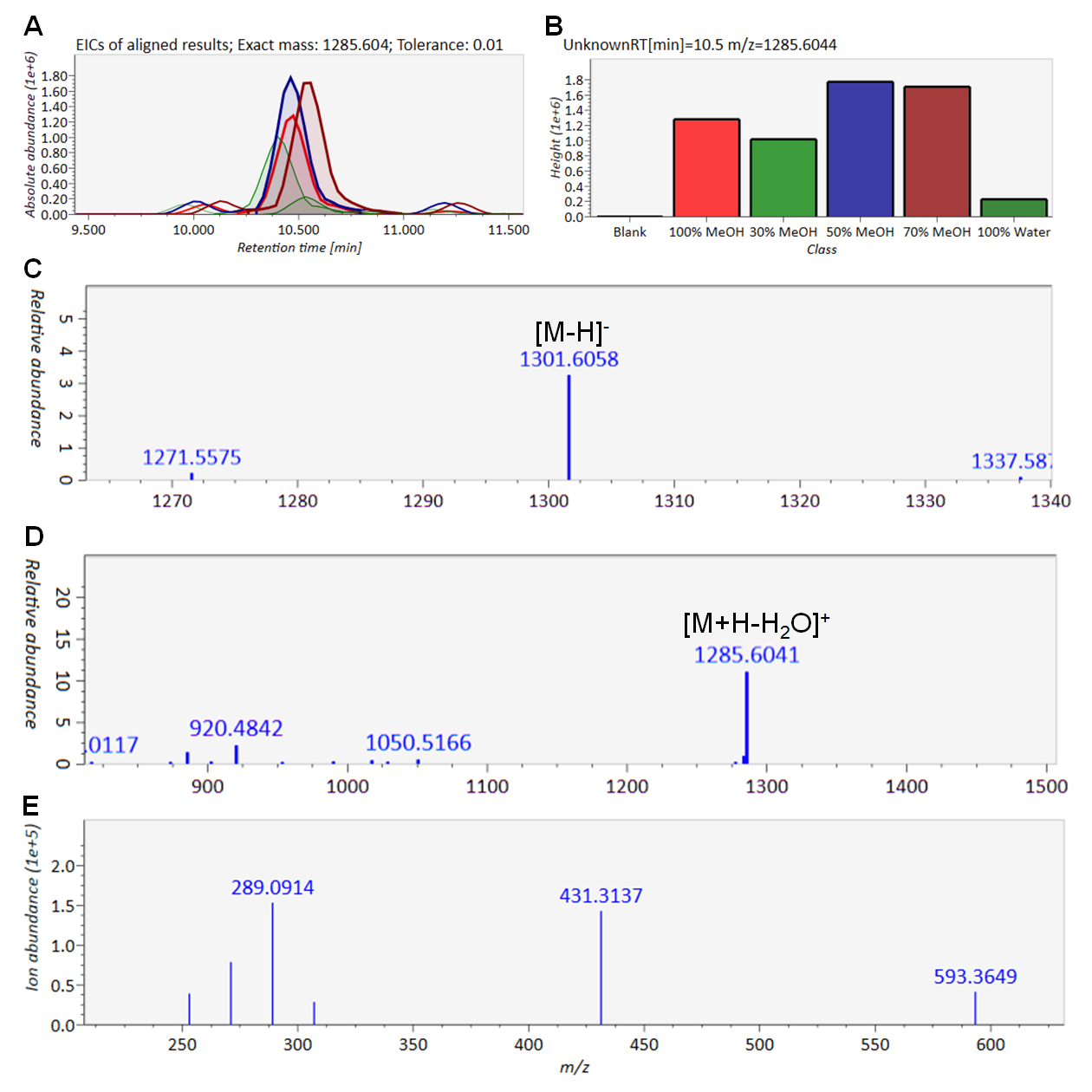


**Figure S19**


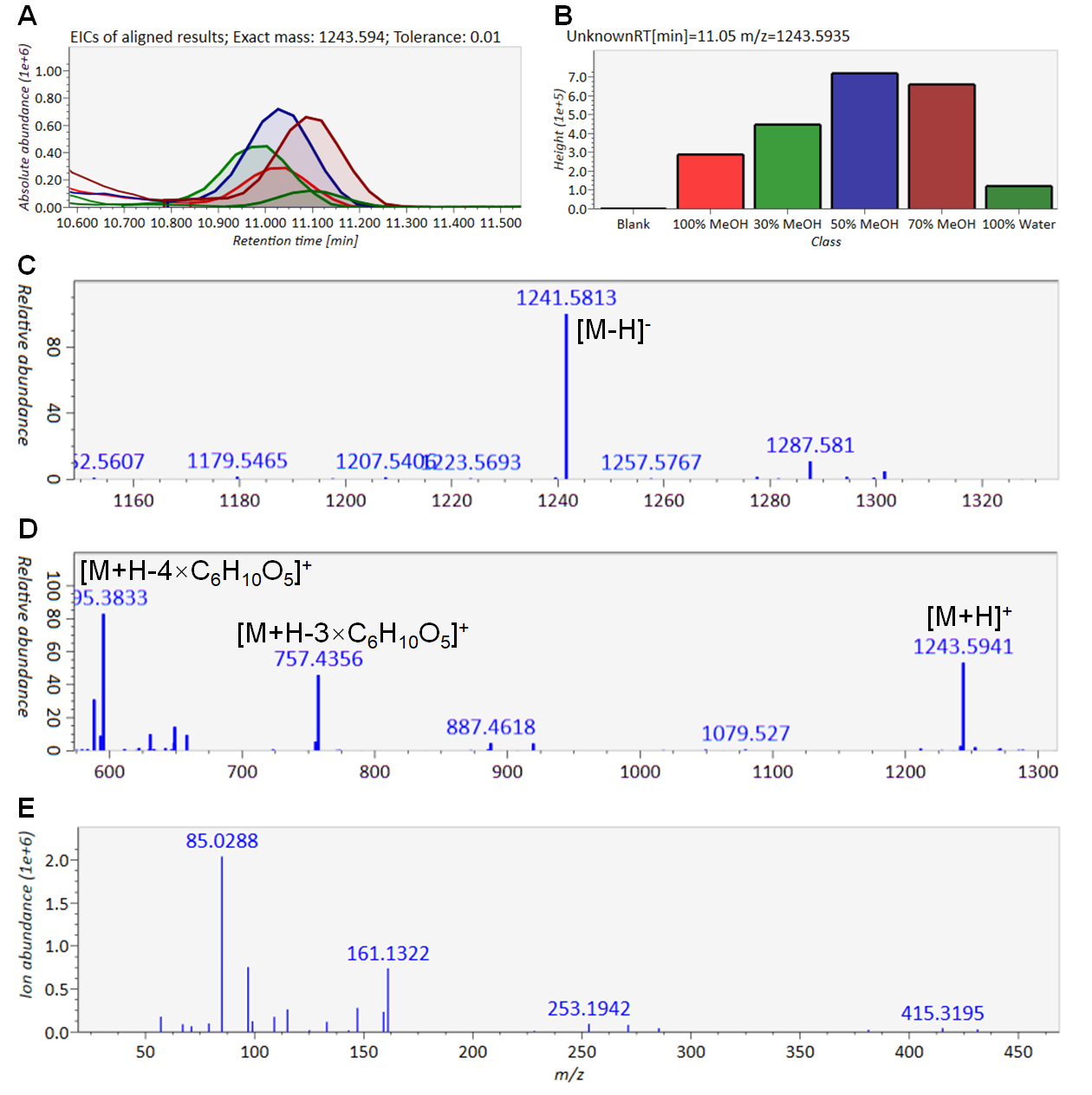


**Figure S20**


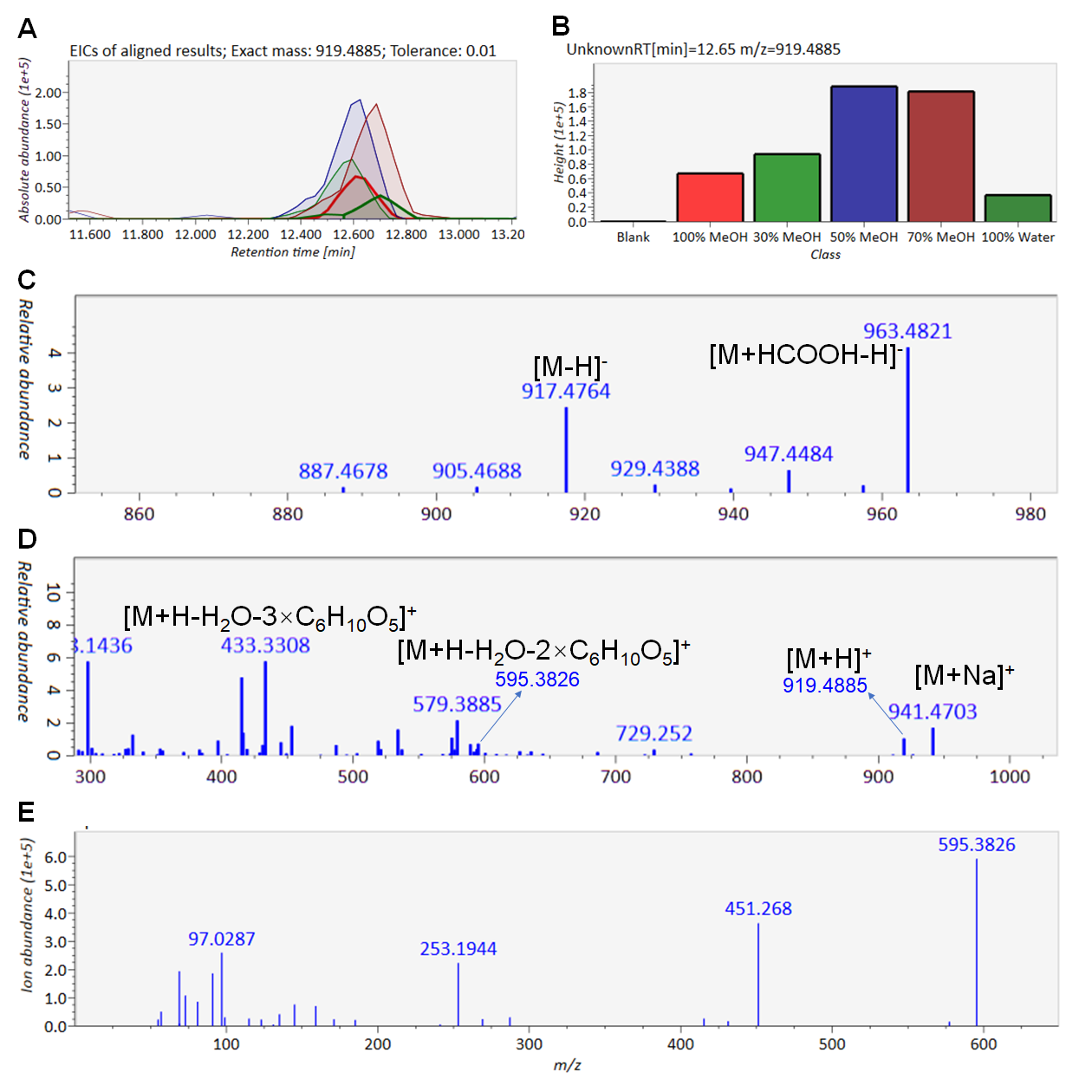


**Figure S21**


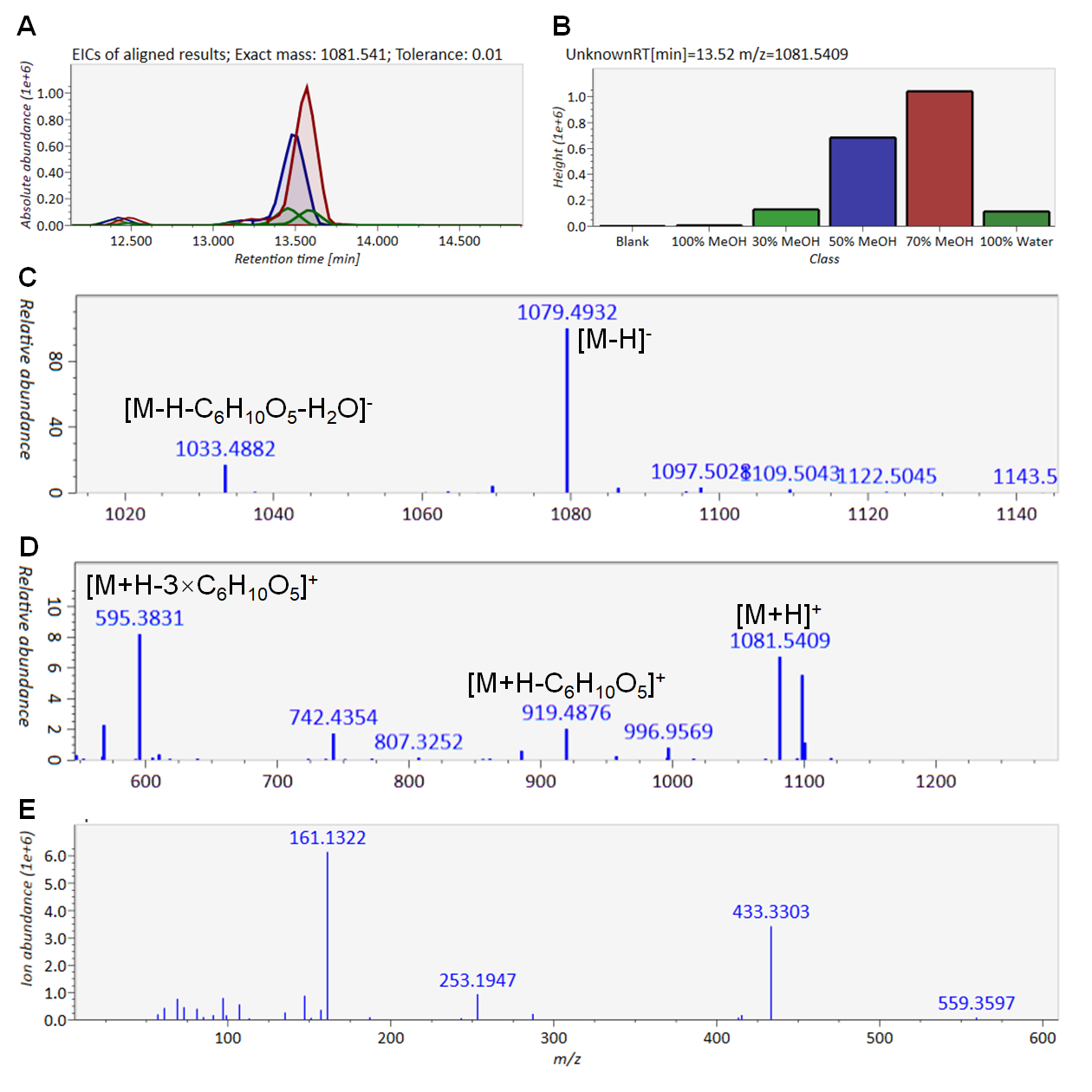


**Figure S22**


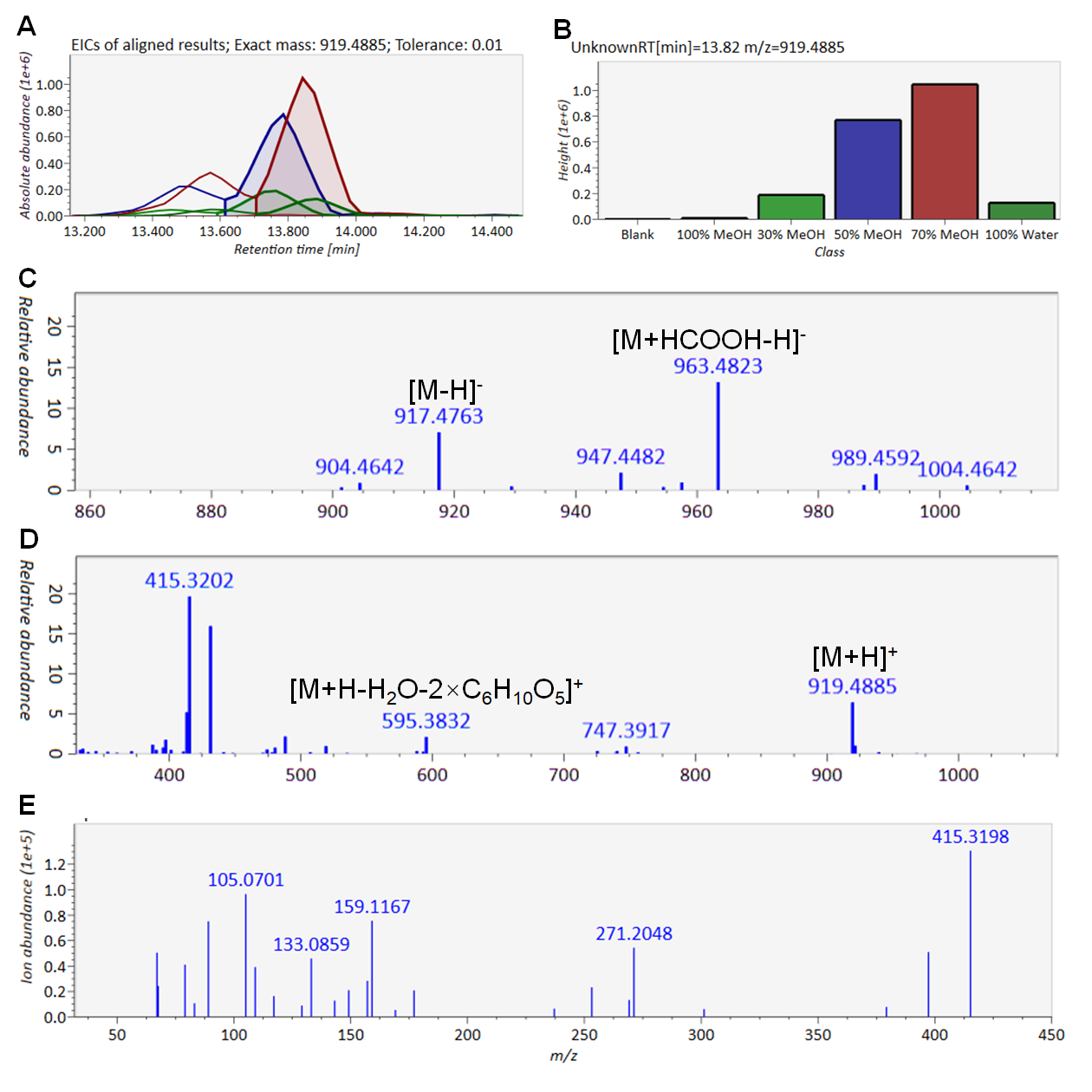


**Figure S23**


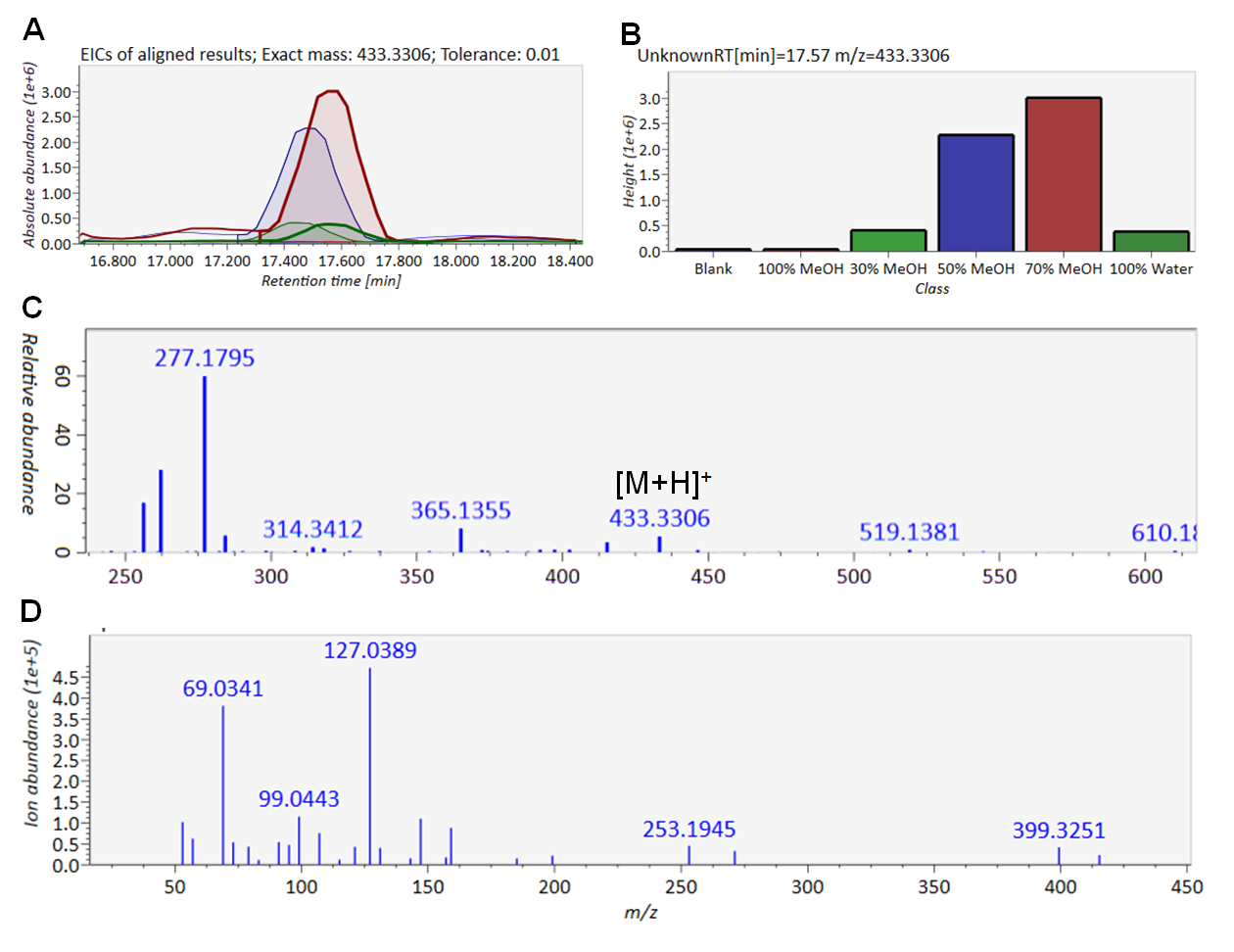


**Figure S24**


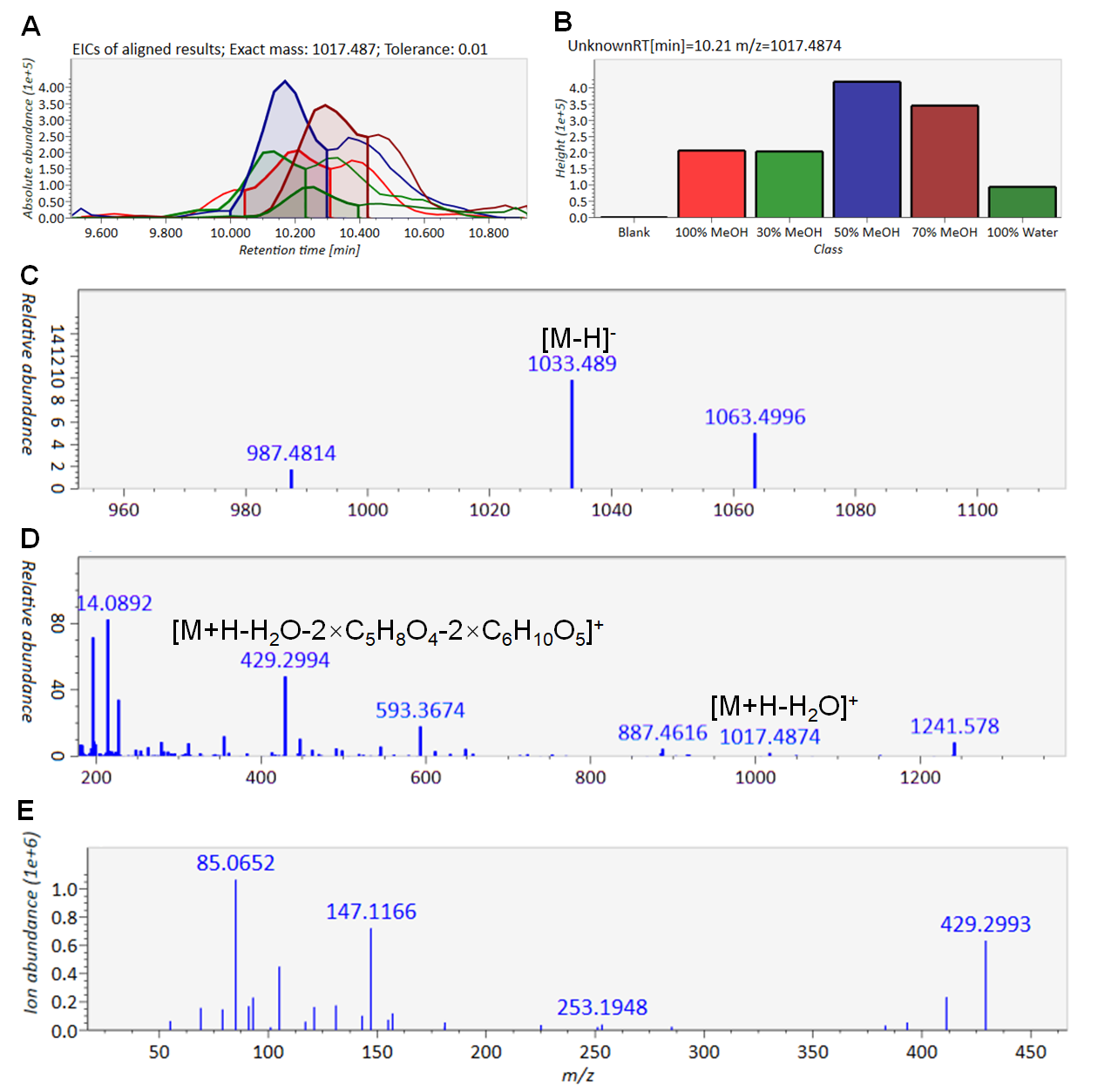


**Figure S25**


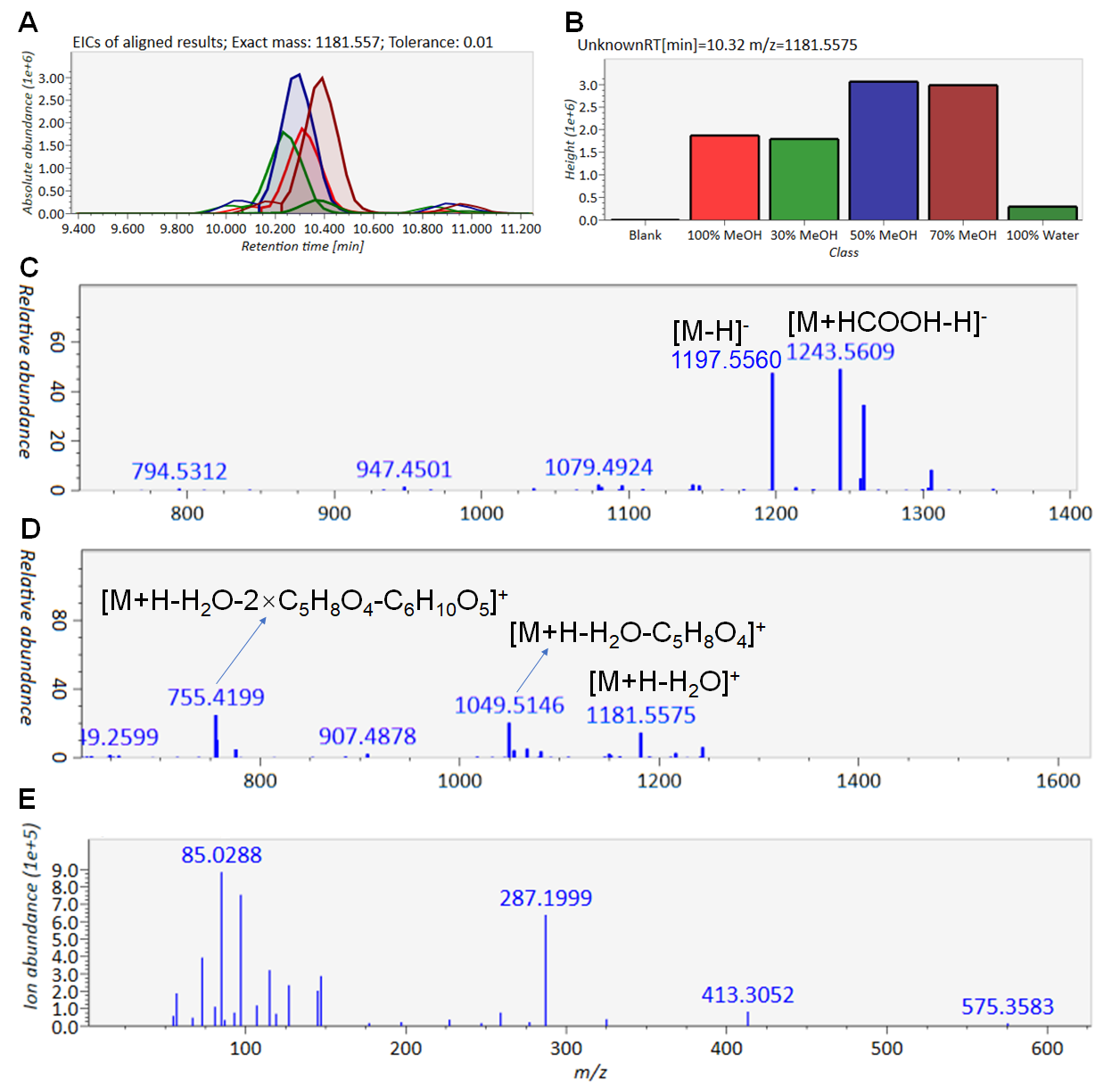


**Figure S26**


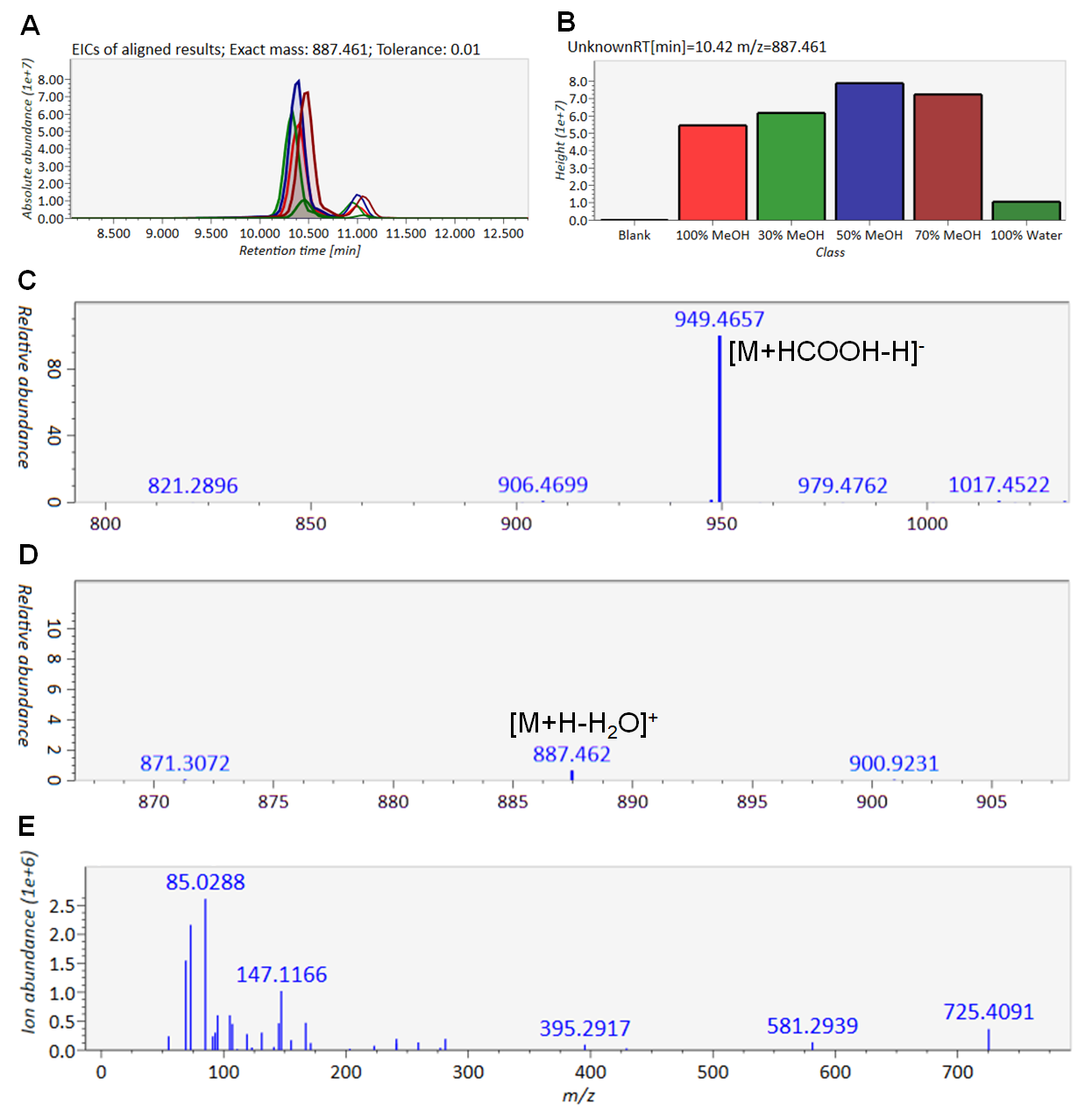


**Figure S27**


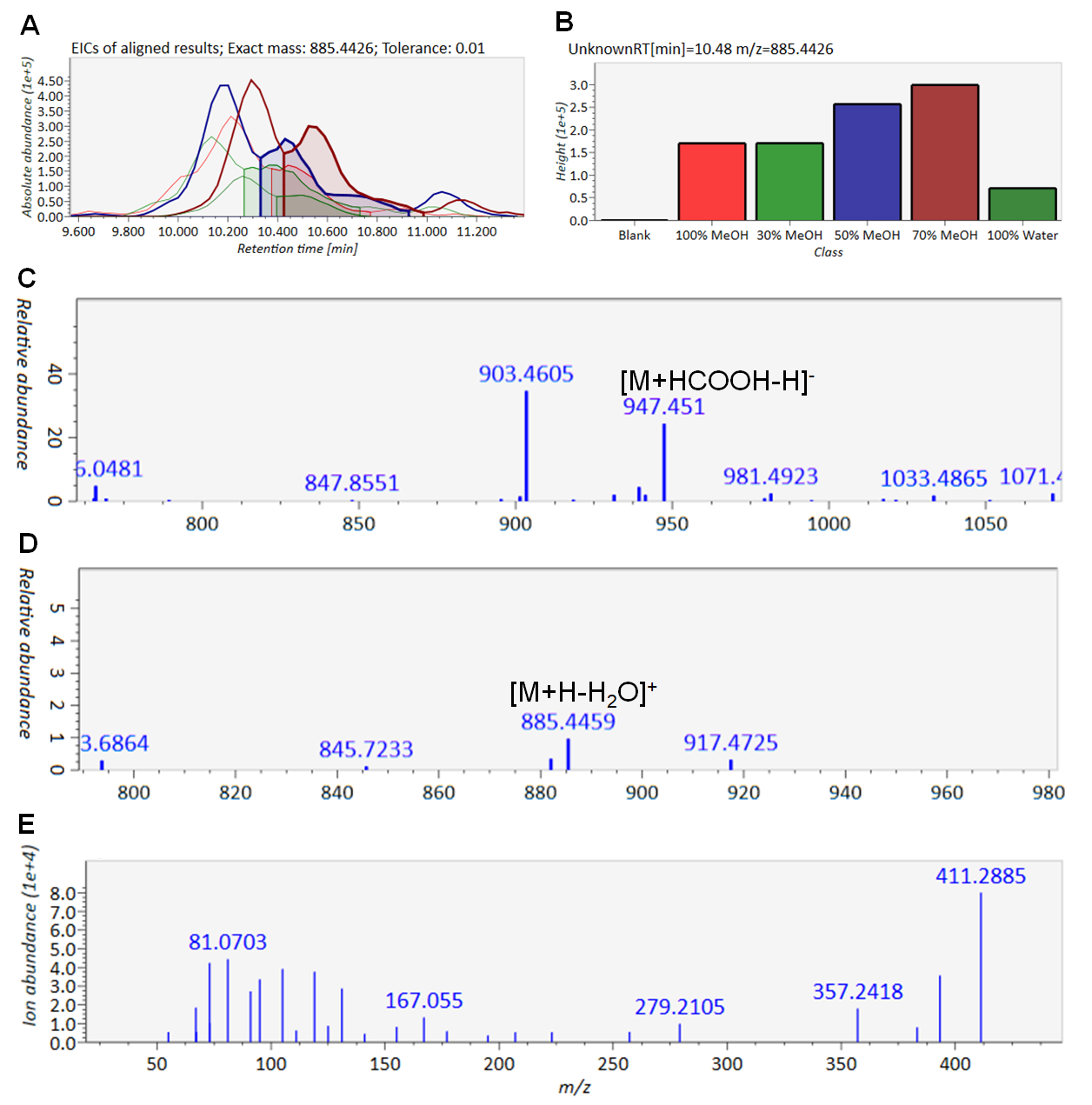


**Figure S28**


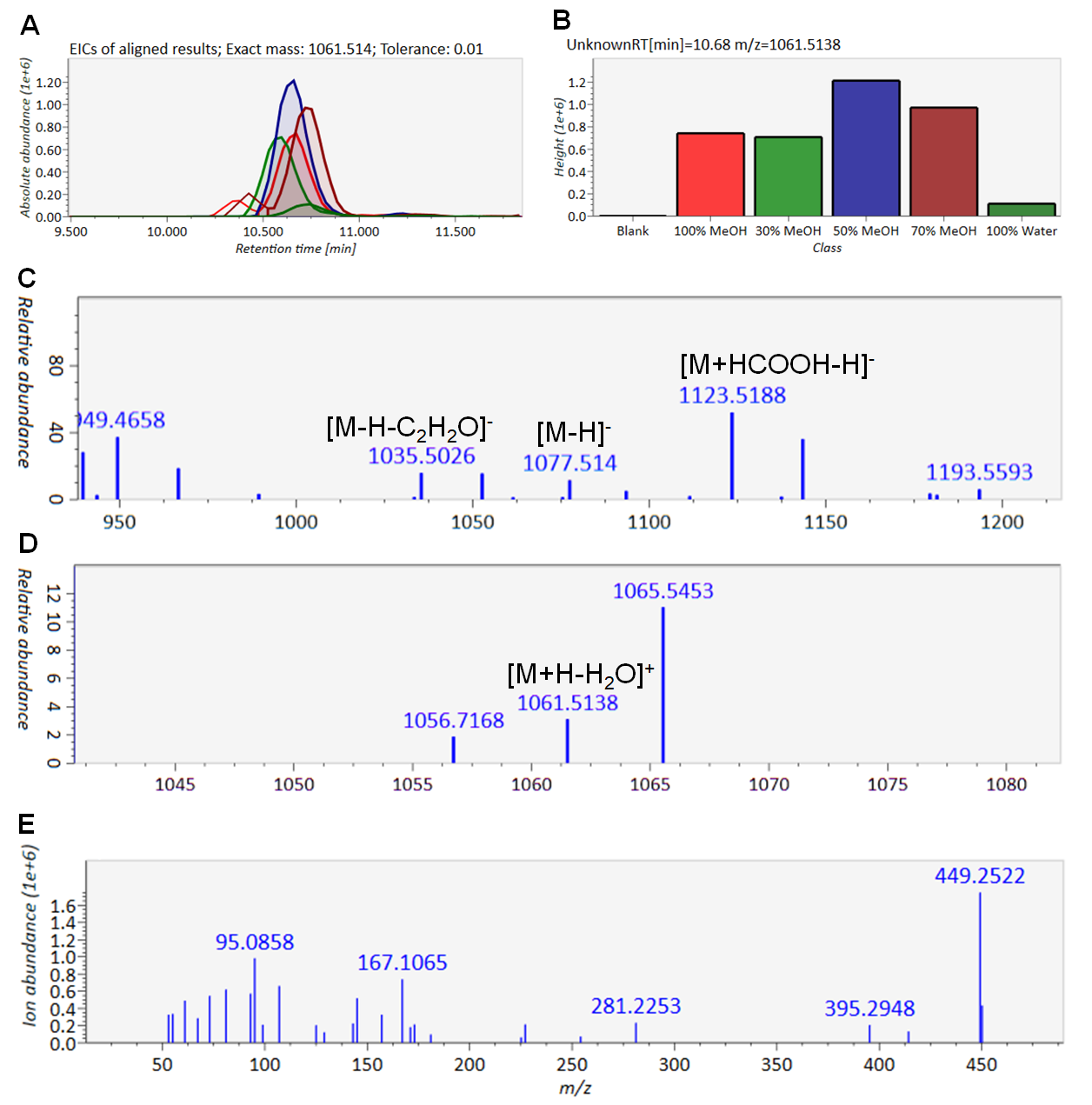


**Figure S29**


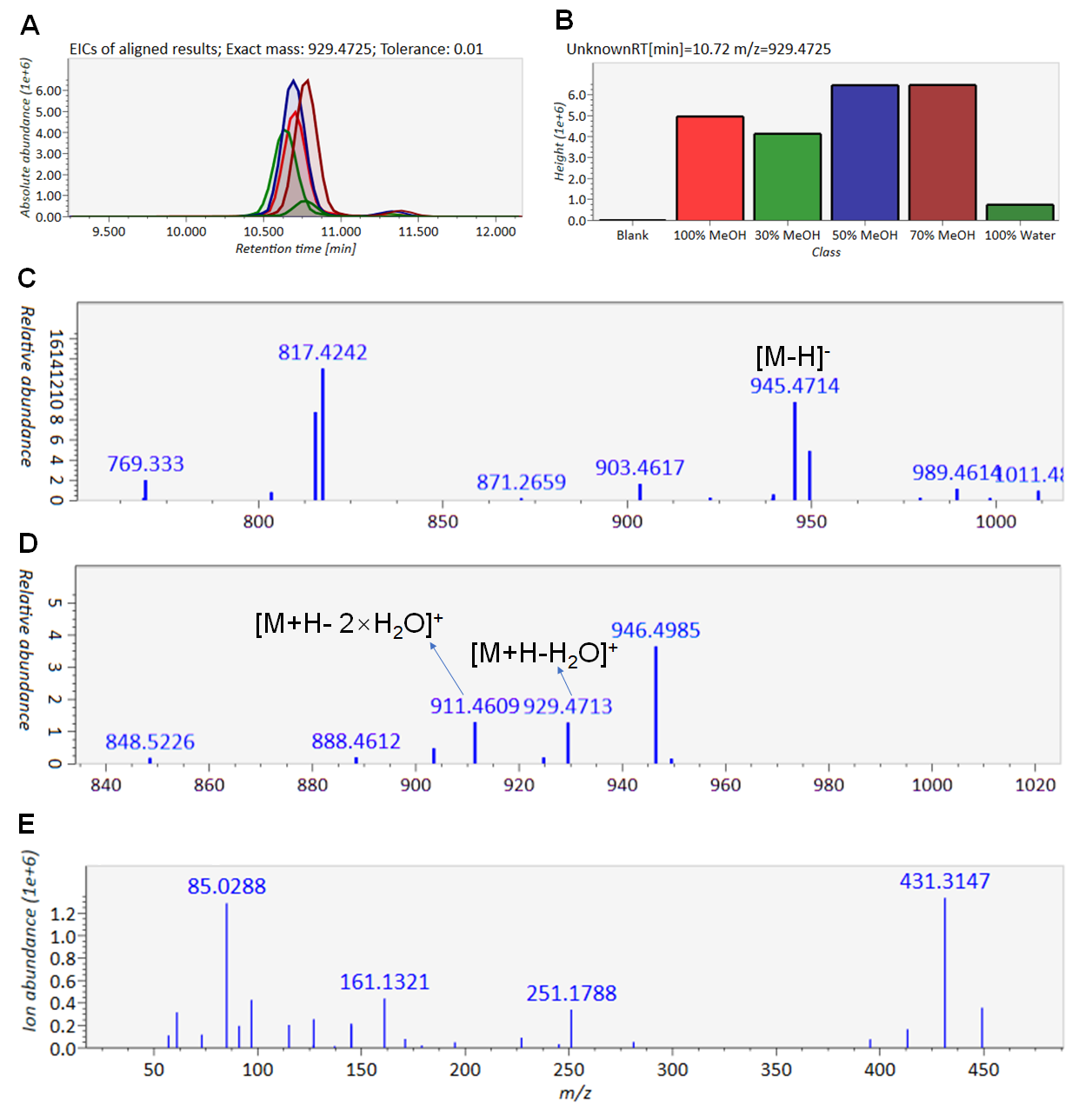


**Figure S30**


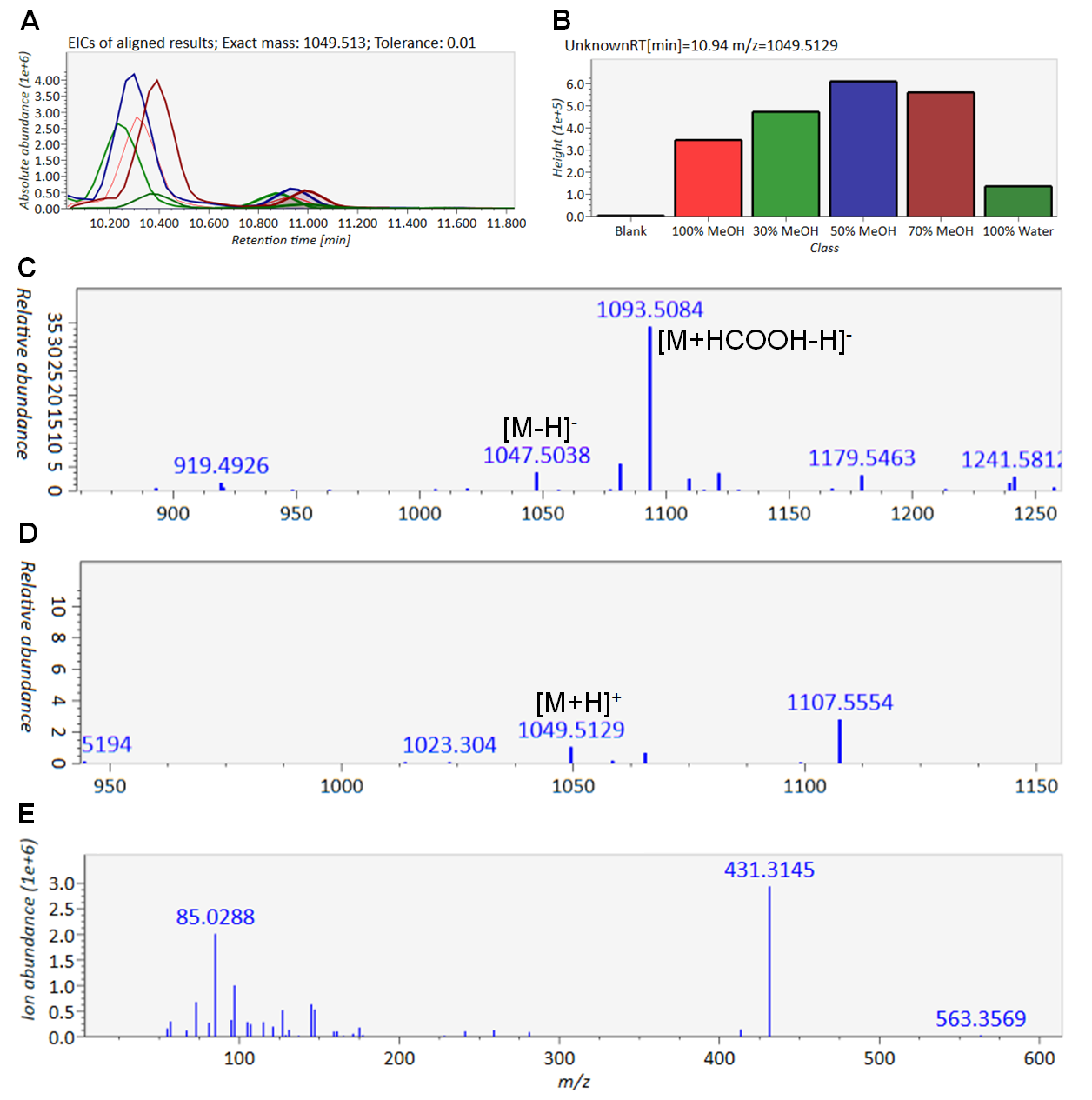


**Figure S31**


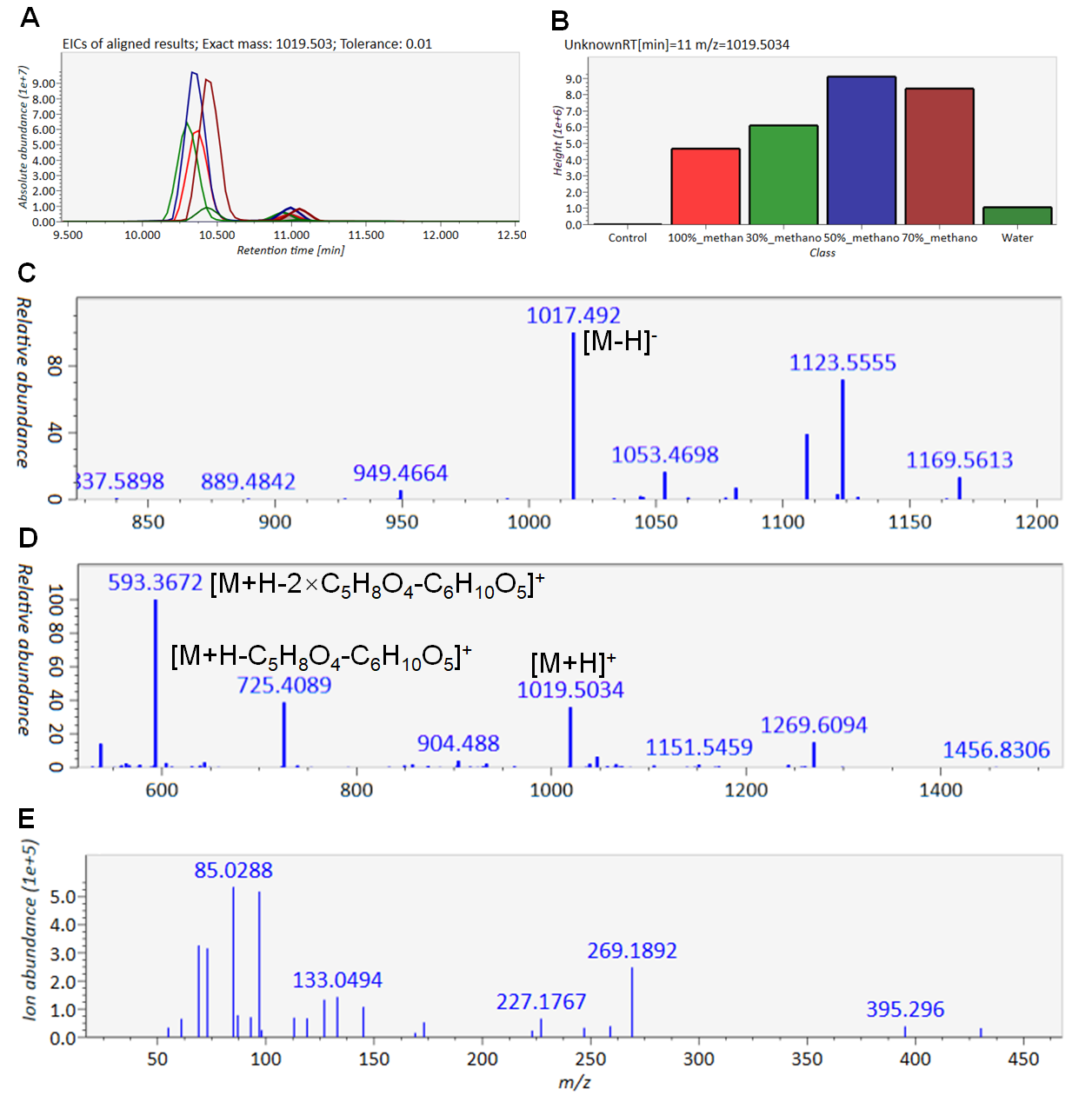


**Figure S32**


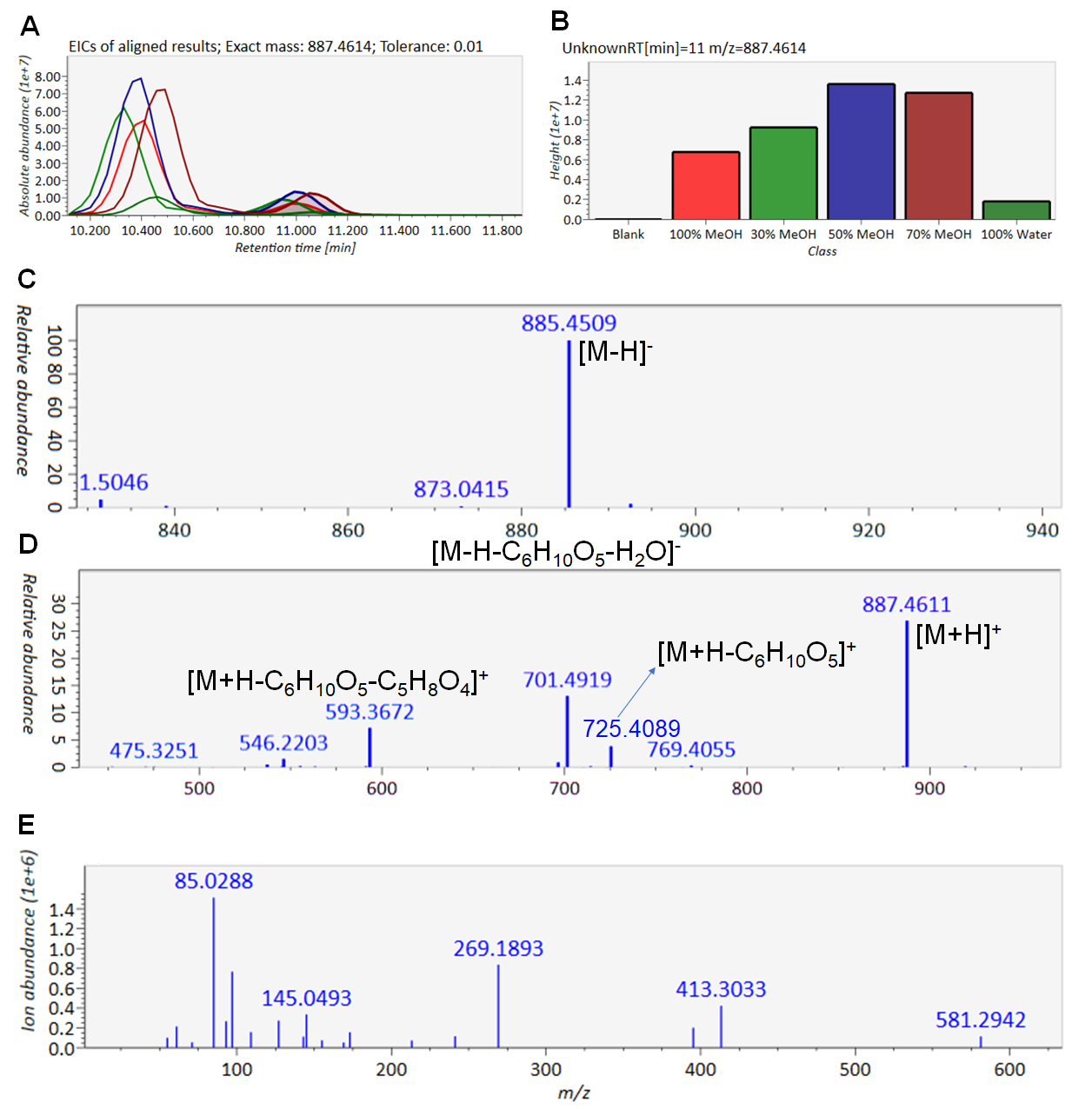


**Figure S33**


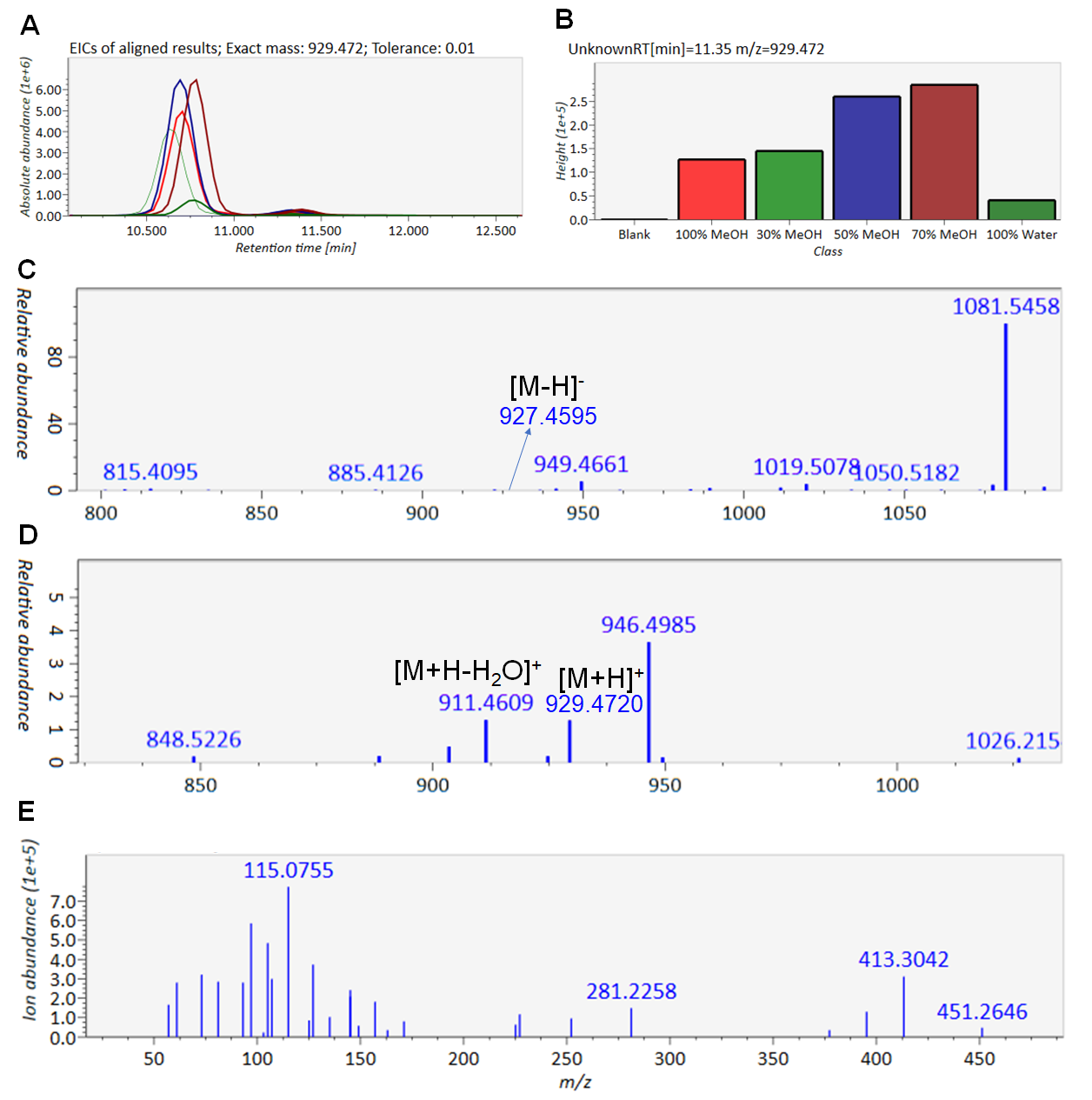


**Figure S34**


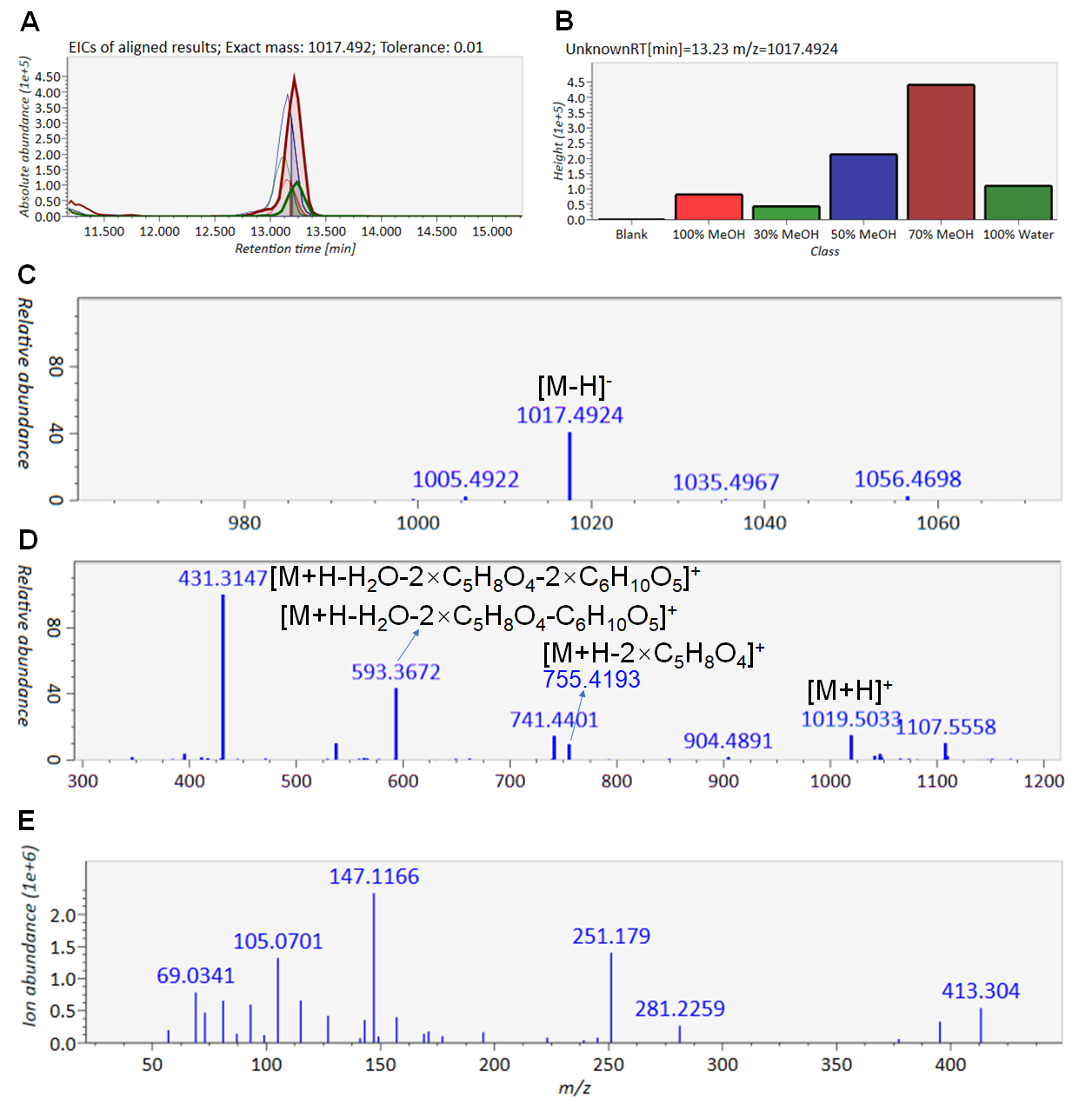


**Figure S35**


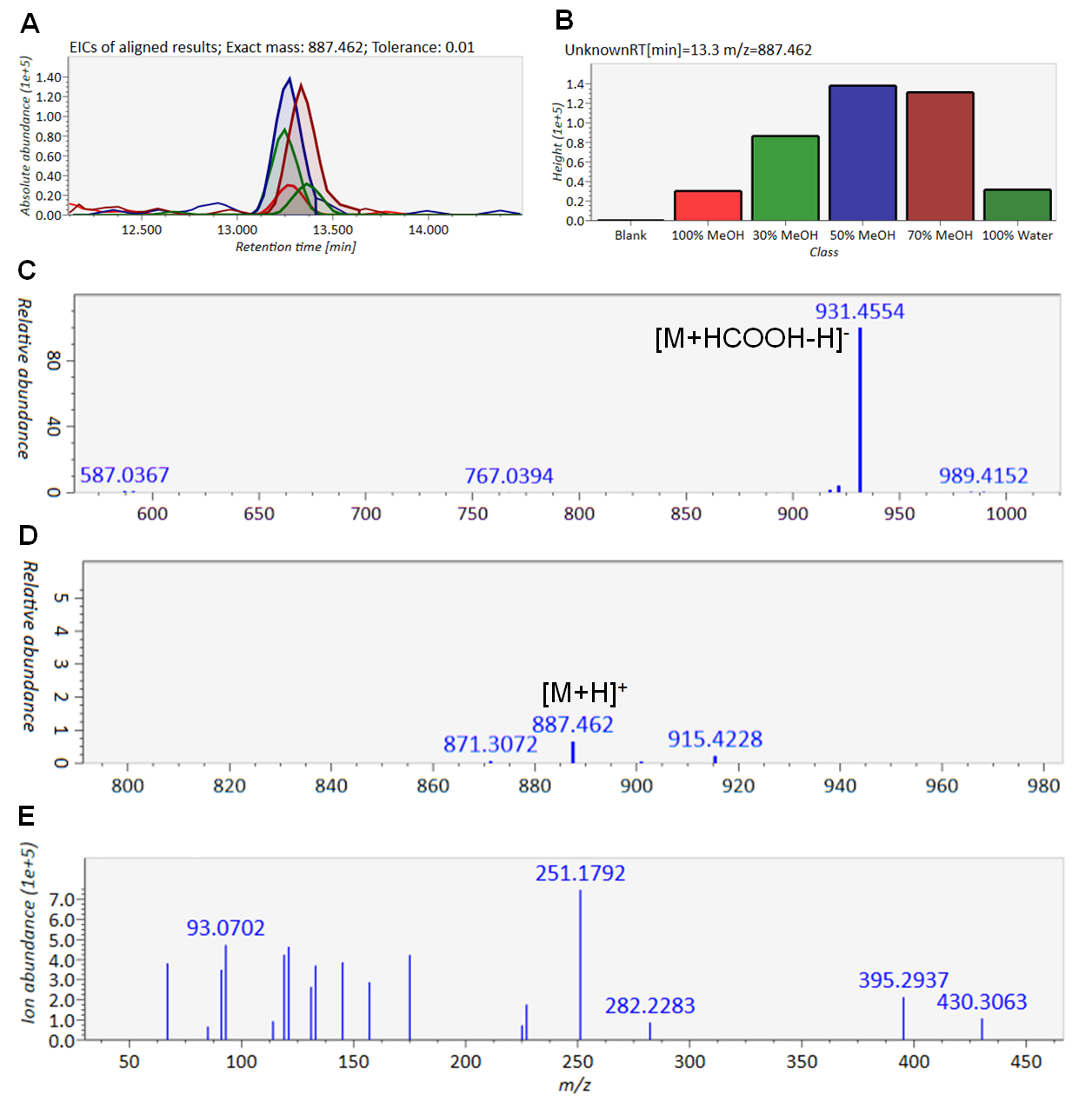


**Figure S36**


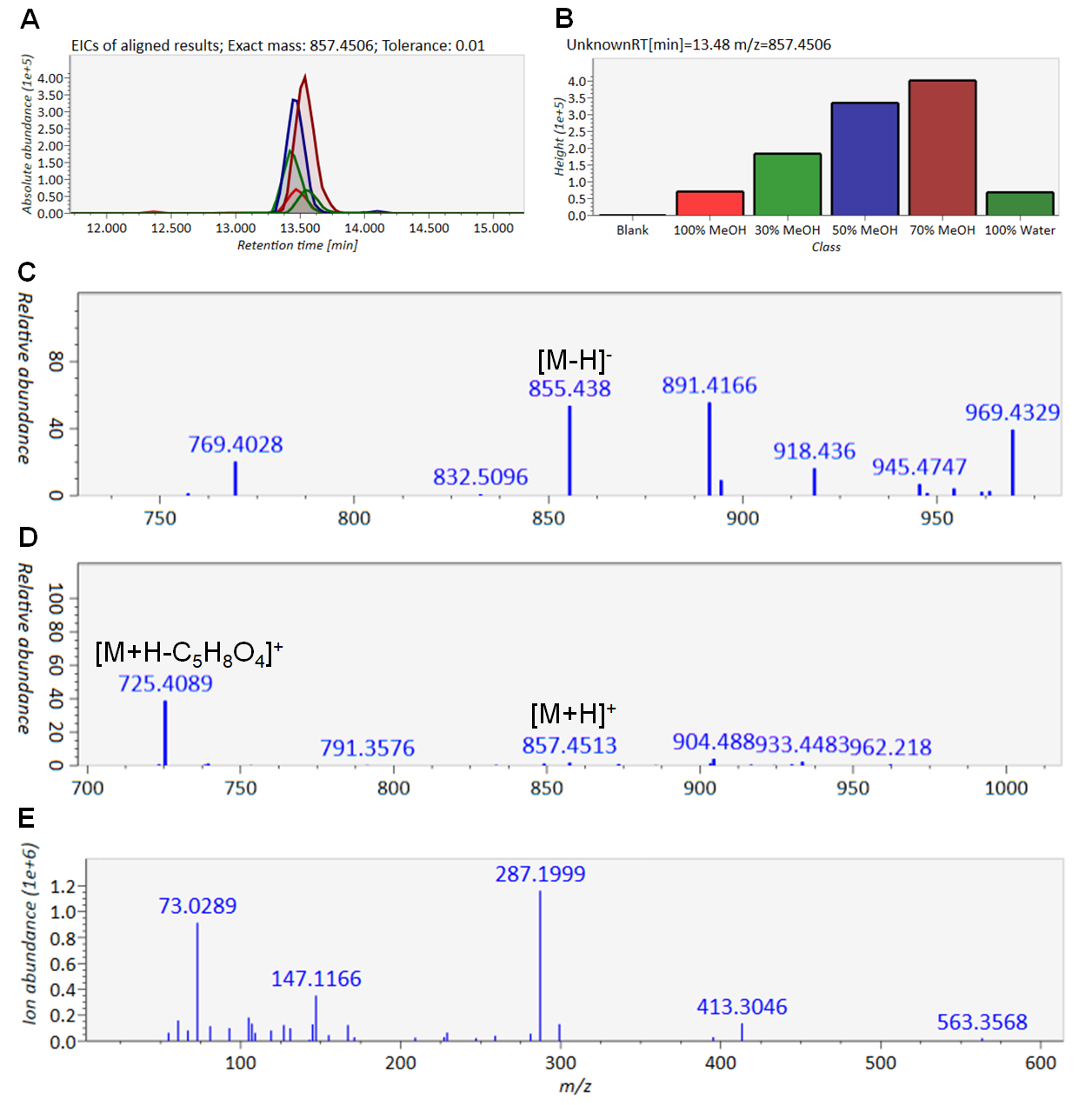


**Figure S37**


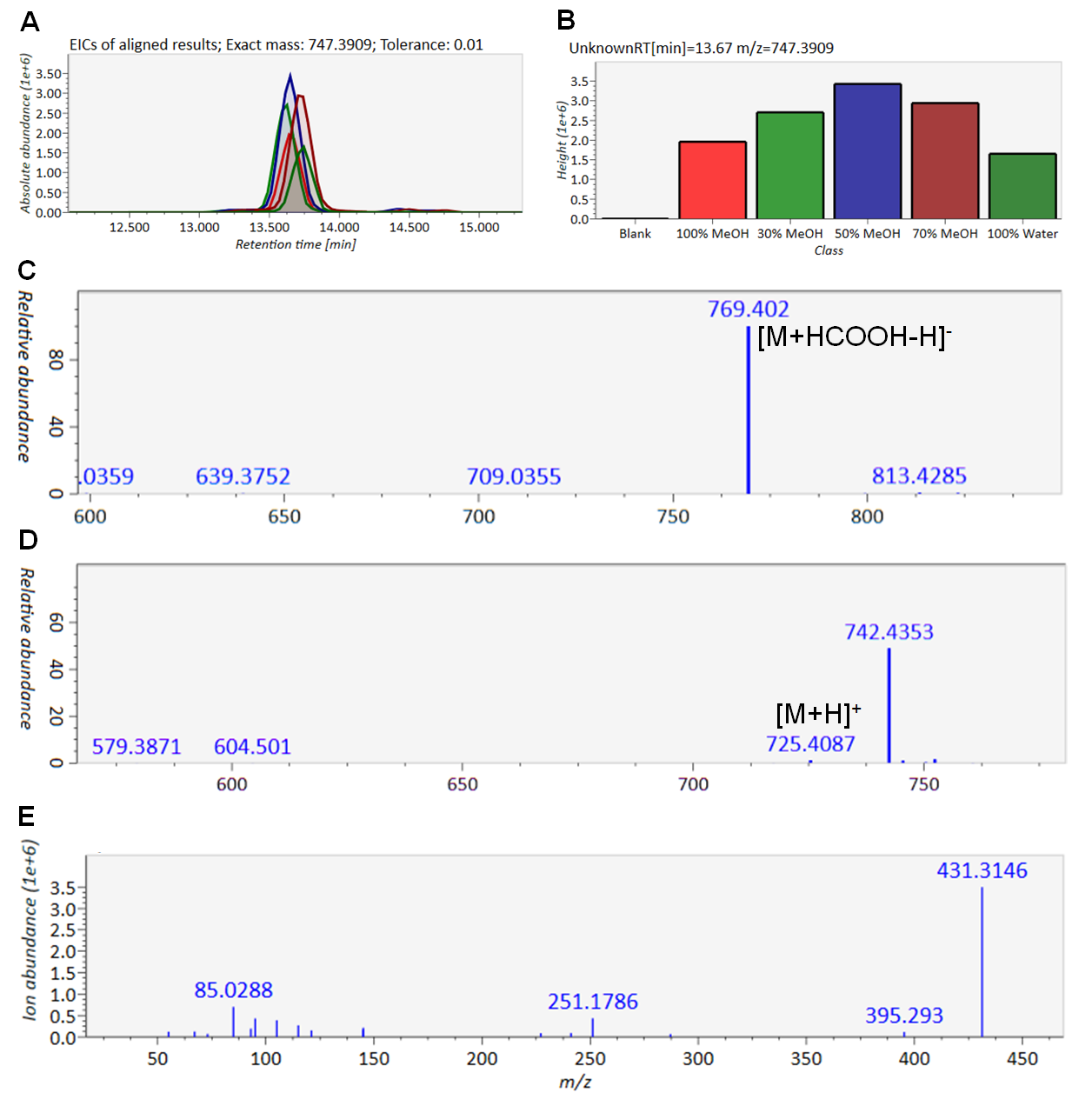


**Figure S38**


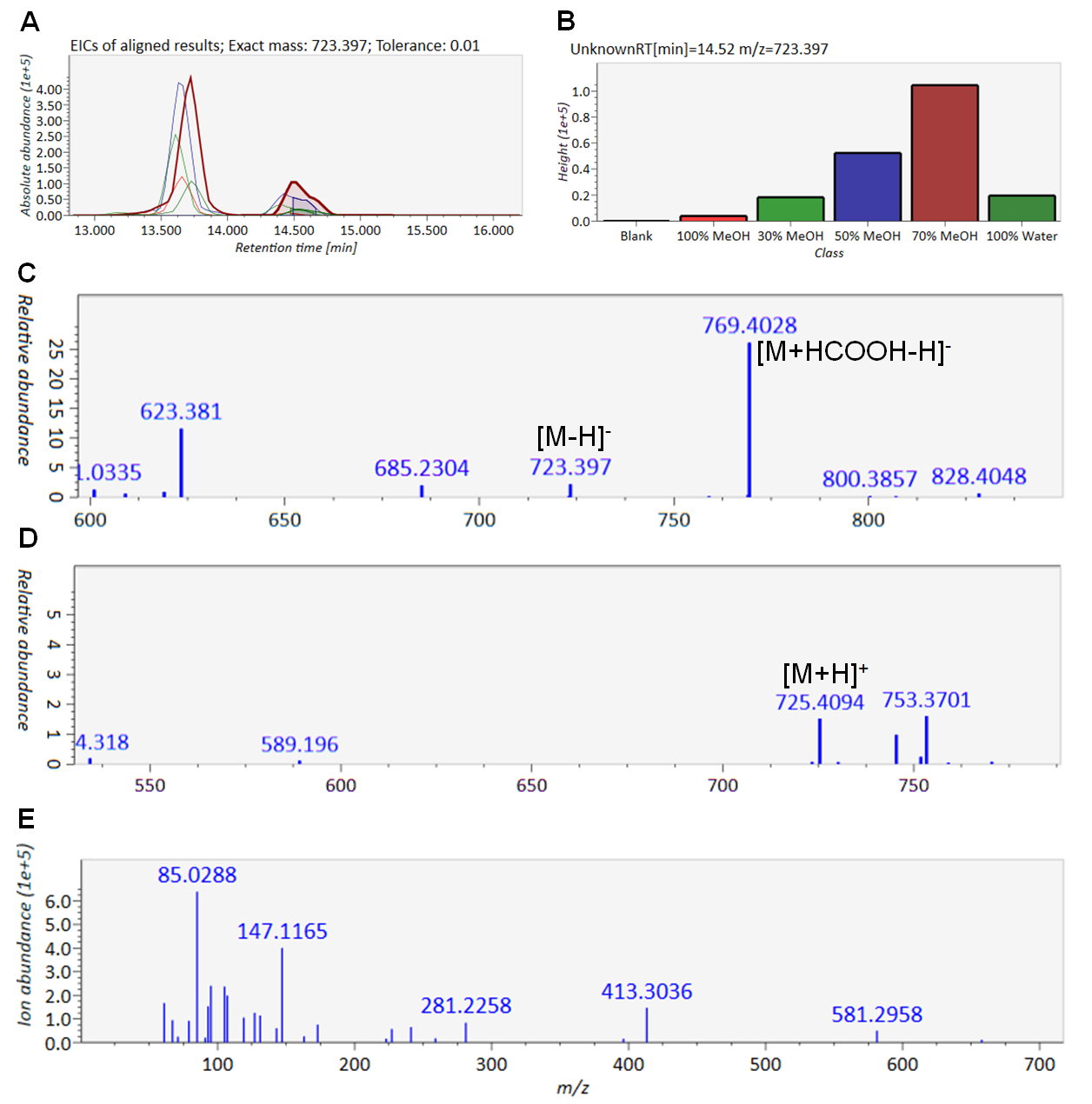


**Figure S39**


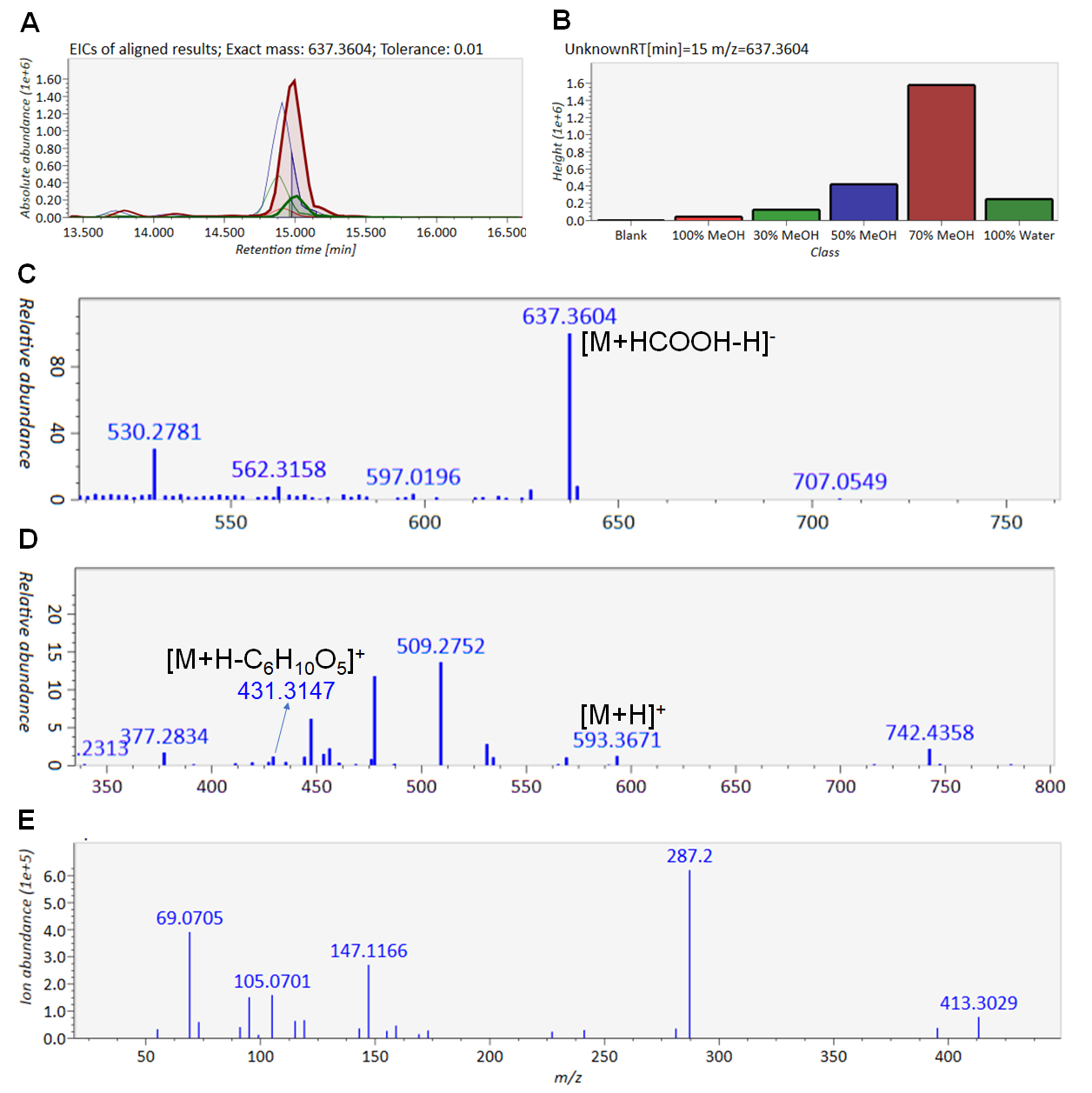


**Figure S40**


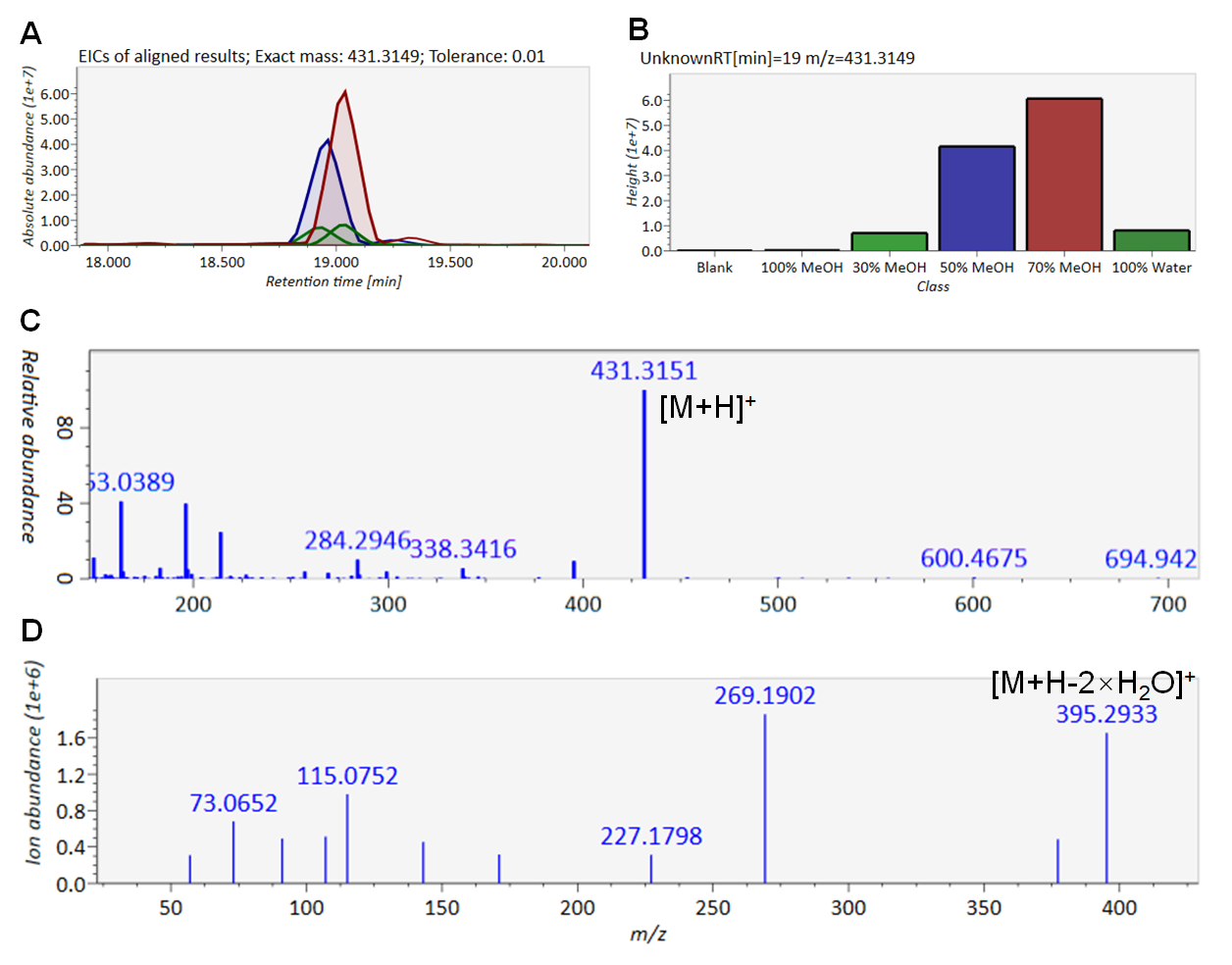


**Figure S41**
